# TPepRet: a deep learning model for characterizing T-cell receptors–antigen binding patterns

**DOI:** 10.1093/bioinformatics/btaf022

**Published:** 2025-01-28

**Authors:** Meng Wang, Wei Fan, Tianrui Wu, Min Li

**Affiliations:** School of Computer Science and Engineering, Central South University, Changsha 410083, China; Nuffield Department of Women’s and Reproductive Health, University of Oxford, Oxford OX39DU, United Kingdom; School of Computer Science and Engineering, Central South University, Changsha 410083, China; School of Computer Science and Engineering, Central South University, Changsha 410083, China

## Abstract

**Motivation:**

T-cell receptors (TCRs) elicit and mediate the adaptive immune response by recognizing antigenic peptides, a process pivotal for cancer immunotherapy, vaccine design, and autoimmune disease management. Understanding the intricate binding patterns between TCRs and peptides is critical for advancing these clinical applications. While several computational tools have been developed, they neglect the directional semantics inherent in sequence data, which are essential for accurately characterizing TCR-peptide interactions.

**Results:**

To address this gap, we develop TPepRet, an innovative model that integrates subsequence mining with semantic integration capabilities. TPepRet combines the strengths of the Bidirectional Gated Recurrent Unit (BiGRU) network for capturing bidirectional sequence dependencies with the Large Language Model framework to analyze subsequences and global sequences comprehensively, which enables TPepRet to accurately decipher the semantic binding relationship between TCRs and peptides. We have evaluated TPepRet to a range of challenging scenarios, including performance benchmarking against other tools using diverse datasets, analysis of peptide binding preferences, characterization of T cells clonal expansion, identification of true binder in complex environments, assessment of key binding sites through alanine scanning, validation against expression rates from large-scale datasets, and ability to screen SARS-CoV-2 TCRs. The comprehensive results suggest that TPepRet outperforms existing tools. We believe TPepRet will become an effective tool for understanding TCR-peptide binding in clinical treatment.

**Availability and implementation:**

The source code can be obtained from https://github.com/CSUBioGroup/TPepRet.git.

## 1 Introduction

T-cell receptors (TCRs), situated on the surface of T lymphocytes, serve as the critical interface eliciting the adaptive immune response through the recognition of antigenic peptides presented by Major Histocompatibility Complex molecules (Siegel *et al.* 2019, Finck *et al.* 2020, Wang *et al.* 2023). They play crucial roles in the clinical treatment of cancer, vaccine development, and the treatment of autoimmune diseases. Investigating the intricate binding patterns between TCRs and peptides can advance personalized immunotherapy and innovative vaccine design, offering fresh prospects for treating certain refractory diseases. TCR is encoded by multiple gene fragments, and for most T cells, these include the α chain and β chain (Davis and Bjorkman 1988, Krogsgaard and Davis 2005). These gene fragments undergo combination during T-cell development through the process of gene recombination, leading to the formation of a complete TCR. This process results in substantial genetic diversity. Utilizing deep learning methods for predicting the binding of TCRs and peptides can aid in screening targets for cancer treatment, offering support for diagnosis, clinical immunotherapy, and the development of new vaccines (Shah *et al.* 2023). Additionally, it can significantly reduce the treatment cycle, opening up possibilities for cancer patient immunotherapy. This surge in data volume presents both opportunities and challenges for improving the accuracy and reliability of TCR-peptide binding predictions.

Currently, some efforts have been made for predicting TCR-peptide binding (Altman *et al.* 1996, Doytchinova *et al.* 2006, Desai and Kulkarni-Kale 2014, Glanville *et al.* 2017, Zhang *et al.* 2018, Kula *et al.* 2019, Huang *et al.* 2020, Peters *et al.* 2020, Montemurro *et al.* 2021, Moris *et al.* 2021, Schmidt *et al.* 2021, Cai *et al.* 2022). Based on the input data types, these tools can be categorized as specific tools or pan tools. Specific tools can only build separate prediction models for peptides with existing binding data and cannot predict the binding of new peptides, including TCRdist (Dash *et al.* 2017), DeepTCR (Sidhom *et al.* 2021), TCRGP (Jokinen *et al.* 2021), TCRex (Gielis *et al.* 2019), NetTCR-2.1 (Montemurro *et al.* 2022), etc. Pan tools can predict the binding of new peptides not present in training data, such as pMTnet (Lu *et al.* 2021), TITAN (Weber *et al.* 2021), TEIM (Peng *et al.* 2023), TEINet (Jiang *et al.* 2023), PanPep (Gao *et al.* 2023), etc. These tools characterize the binding of common and uncommon peptides to TCR by modeling the TCR binding to each peptide separately or all available peptides together (Moris *et al.* 2021, Zhang *et al.* 2021, Chen *et al.* 2024). However, the structure of these modules significantly undermines the integrity and correlation of the sequence direction for two input sequences, resulting in poor prediction performance. Moreover, the current tools have not mined and visualized the binding preferences of each peptide, potentially causing confusion for users in clinical applications (such as T-cell therapy, tumor vaccine, etc.). Consequently, there is a need for new modeling tools to enhance the prediction of TCR-peptide binding and provide support for immunotherapy.

To address these problems, we developed TPepRet. Specifically, TPepRet has two main modules: the BiGRU (Cho *et al.* 2014) module and the RetNet (Sun *et al.* 2023) module. (i) The BiGRU module comprehensively learns information from different subsequences along the sequence direction, ensuring the integrity and sequential information during model training. (ii) The RetNet, a foundational architecture for Large Language Models, introduces the “D” matrix. This matrix is more aligned with sequence processing in terms of position perception compared to traditional Transformer position encoding. We divide the test set into a seen set and an unseen set, representing whether the peptides in each entry of the set have appeared in the training set, respectively. The two test sets also reflect the tool’s ability to learn and reason from known data and its ability to generalize to unknown data. We conduct various tests to assess the superiority of TPepRet. Firstly, the performance on the seen set far exceeds that of the reference tool. Secondly, we perform a more rigorous comparison and analysis of the tool results, which includes extracting publicly available peptides from the training sets of reference tools. We also compare the results of applying TPepRet’s architecture to the training data of the reference tool. The findings suggest that TPepRet is currently the most effective tool for predicting TCR-peptide binding. Furthermore, we visualize the binding preferences of each peptide at various sites along the CDR3 fragments on TCRs. Additionally, a comparative analysis is conducted between the predicted outcomes of TPepRet and the reference tools, showcasing the robust recovery ability of TPepRet for peptide prediction preferences. Two single-cell prediction tasks demonstrate the 0–1 classification ability of TPepRet in T-cell clonal expansion rate. Moreover, an analysis of positive peptide mixing predictions and an assessment of the importance of binding sites are carried out. Lastly, a significant positive correlation between TPepRet score and the antigen expansion rate is demonstrated, employing an additional extensive TCR-peptide binding library. All these aspects indicate that TPepRet is a robust tool for predicting TCR-peptide binding, and it can provide valuable assistance for clinical diagnosis and the development of new immunotherapy methods.

## 2 Materials and methods

### 2.1 Overview of TPepRet’s framework

**Figure 1.**
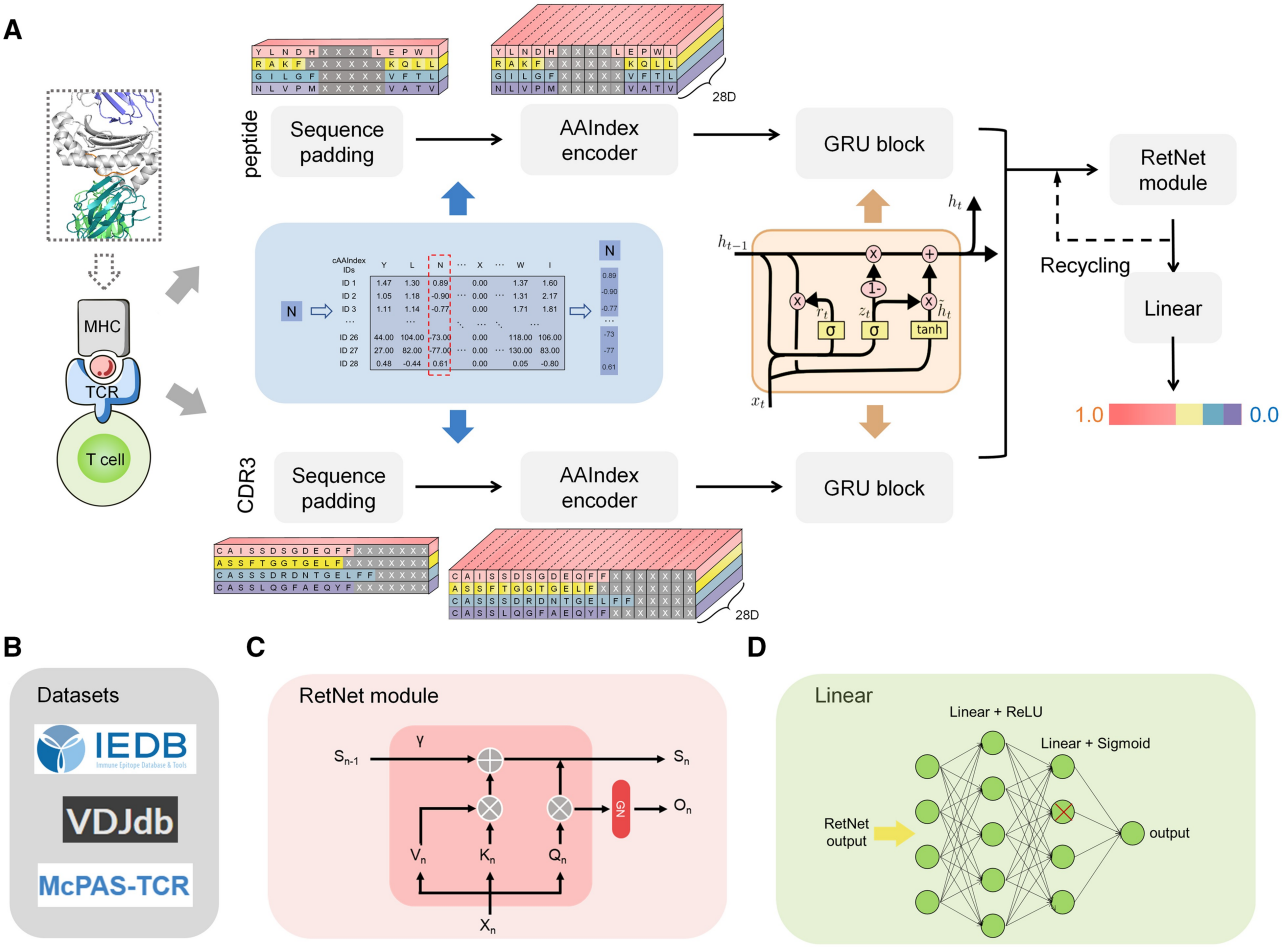
(A) TPepRet framework. The RetNet module goes through 24 layers of recycling. (B) The main training datasets of TPepRet. (C) The main process in RetNet. (D) Details of the final linear layer of TPepRet. The cross line indicates unit dropout.

TPepRet is built upon the BiGRU and RetNet modules, and its model architecture is illustrated in Fig. 1A. Initially, the model’s input is divided into two components: peptides and CDR3s. After sequence padding, each amino acid in these sequences is encoded using 28 relevant amino acid physicochemical features extracted from AAIndex (Kawashima and Kanehisa 2000). The main training datasets of TPepRet are shown in Fig. 1B. The selected entries in AAIndex are shown in Supplementary Table S1. Subsequently, the encoded sequences are separately input into the BiGRU blocks. These modules independently learn contextual information about the sequences, encompassing the context of the subsequences. After concatenating the two outputs from the BiGRU blocks, they are fed into the RetNet module, which is connected in a series at 24 layers. The RetNet module is a variant of self-attention, as depicted in Fig. 1C. This module introduces the D matrix and Group Normalization (GN) module. The exponential decay factor in the D matrix, γ, enables RetNet to retain the semantic impact of sequence proximity during the learning process. Additionally, the GN module introduces nonlinearity, facilitating parallel training of the module. In essence, the incorporation of RetNet modules has significantly enhanced the model’s understanding of sequences and improved training speed. After exiting the RetNet modules, the model undergoes two linear + ReLU layers and ultimately passes through Sigmoid to map the output between 0 and 1 (Fig. 1D). This final output represents the predicted binding possibility of the model. TPepRet’s results are cross-validated using 5-fold cross-validation, and the output is the average of the five model outputs.

Building upon thorough research on existing tools, we have assimilated their experiences and analytical conclusions to enhance prediction performance. For instance, in Nielsen’ (Montemurro *et al.* 2022) and Li’ (Jiang *et al.* 2023) studies, various methods for generating negative samples are compared, revealing that random pairing to obtain negative samples is the optimal approach. In the research conducted by Yao (Zhao *et al.* 2023), a pre-trained model is employed to encode sequences, but the impact on performance is not substantial. The studies by Nielsen (Montemurro *et al.* 2022) and Liu (Gao *et al.* 2023) observe that the key binding site on CDR3 is situated in the middle of the sequence. Consequently, we do not adopt the strategy of filling “X” amino acids in the middle of the CDR3 sequence during the training and testing processes. Instead, we fill the “X”s at the tail end of the CDR3 sequence.

Moreover, our structural observations, such as in the binding complex where peptides directly interact with the CDR3 portion of TCRs (Nowak *et al.* 2016), indicate that this interaction is the crucial factor influencing their binding. Therefore, we select the CDR3 fragments of TCRs only. In addition to the advantages of the model framework, these experiences and analytical conclusions are paramount in informing our modeling approach.

### 2.2 Datasets

We integrated TCR-peptide binding data from the IEDB (Vita *et al.* 2019), VDJdb (Shugay *et al.* 2018), and McPAS (Tickotsky *et al.* 2017) databases, resulting in a total of 83 765 positive samples. These samples were obtained by selecting the CDR3 on the TCR β chain, which directly interacts with peptide binding and is commonly used for modeling. Subsequently, we purified the training data based on the binding data characteristics. We standardized the length of peptides to [8,12] and CDR3s to [, 20]. Employing the commonly used strategy of random pairing (Montemurro *et al.* 2022), we obtained negative samples, maintaining a 1:1 ratio of positive and negative samples. Additionally, we extracted peptides with fewer than 10 positive CDR3 samples into the unseen set to evaluate the model’s generalization ability. The remaining data were divided into groups based on the peptide and allocated to the training set (4/5 of the data) and the seen set (1/5 of the data). Finally, the training set comprised a total of 1 29 278 entries, and the seen set included 32 116 entries. The unseen set preserved 6134 entries that had not been seen in any of the reference tools’ training sets. More details about datasets can be found in Supplementary Materials and in Supplementary Fig. S1.

### 2.3 BiGRU module

Recurrent neural network (RNN) is a deep learning model capable of processing time series information. It has found extensive applications in natural language and biological information processing. Long short-term memory (LSTM) and Gate Recurrent Unit (GRU) represent the two most common variants of RNN designed to mitigate the issues of gradient disappearance and gradient explosion. In comparison to LSTM, GRU, a simplified version, exhibits superior performance in numerous tasks. The GRU (Cho *et al.* 2014) updates the current output by adjusting the weights associated with the update gate, reset gate, and current state. BiGRU consists of unidirectional and opposing GRUs, and its output is determined by the states of the two GRUs. The primary calculation formulas are as follows:


$$z_{t}=\sigma \left(W_{z}\cdot \left[h_{t-1},\;x_{t}\right]\right),$$

where *z_t_* represents the value of the update gate, *σ* is the sigmoid activation function, *W_z_* is the weight matrix, *h_t_*_-1_ is the state at the last moment, *x_t_* represents the input at this time, and [] denotes the concatenation of two matrices.


$$r_{t}=\sigma \left(W_{r}\cdot \left[h_{t-1},x_{t}\right]\right),$$

where *r_t_* represents the value of the reset gate, and *W_r_* is the weight matrix.


$${\tilde{h}}_{t}=\tanh\left(W\cdot \left[r_{t}*h_{t-1},x_{t}\right]\right),$$

where *h͂_t_* represents the input gate of the current state, *tanh*(.) is the activation function, and *W* represents the weight of the input gate.


$$h_{t}=\left(1-z_{t}\right)*h_{t-1}+z_{t}*{\tilde{h}}_{t},$$

where *h_t_* is the updated state at the current point in time, representing a balance between the current input and the past state.

### 2.4 RetNet module

RetNet (Sun *et al.* 2023) is a model published in August 2023, serving as the infrastructure for large-scale language models. It achieves training parallelism, low-cost inference, and excellent performance. RetNet is composed of L identical blocks stacked together. Each RetNet block contains two modules: Multi-scale Retention and Feedforward Network. Compute vector representation once per layer *X^l^* = *RetNet_l_*(*X^l^*−1), where *l* ∈ [1, *L*].

For the *n*-th timestep, the output is calculated as:


$$S_{n}=\gamma S_{n-1}+K_{n}^{T}V_{n}$$

$$\text{Retention}\left(X_{n}\right)=Q_{n}S_{n},\;n=1,\;\cdot \;\cdot \;\cdot ,\;\left|x\right|$$

*γ* is the time factor representing the decay at each step.

The retention layer is defined as:


$$Q=\left(XW_{Q}\right)⊙\Theta ,\;K=\left(XW_{K}\right)⊙\bar{\Theta },V=XW_{V},$$

Θn=einθ,Dnm=γn-m, n≥m0, n<m,

$$\text{Retention}\left(X\right)=(QK^{T}⊙D)V,$$

where $\bar{\Theta }$ is the complex conjugate of Θ, and *D* ∈ ℝ^|x|×|x|^ combines causal masking and exponential decay along relative distance as one matrix.

## 3 Results

### 3.1 TPepRet outperforms existing tools in different conditions

To validate TPepRet’s predictive performance, the collected data is further segmented. The curated data is segregated into a training set and a testing set. The test set is further categorized into a seen set and an unseen set based on whether peptides have appeared in the training set (refer to the Materials and methods section for details). Subsequently, we evaluate three tools newly released in 2023 with the same input as the reference tool—TEIM (Peng *et al.* 2023), TEINet (Jiang *et al.* 2023), and PanPep (Gao *et al.* 2023). We also train a ProtBERT-based prediction model to serve as a baseline. We evaluate the Area Under the Curve (AUC) and Area Under the Precision-Recall Curve (AUPR) of these tools under three different conditions in the seen set.

**Figure 2.**
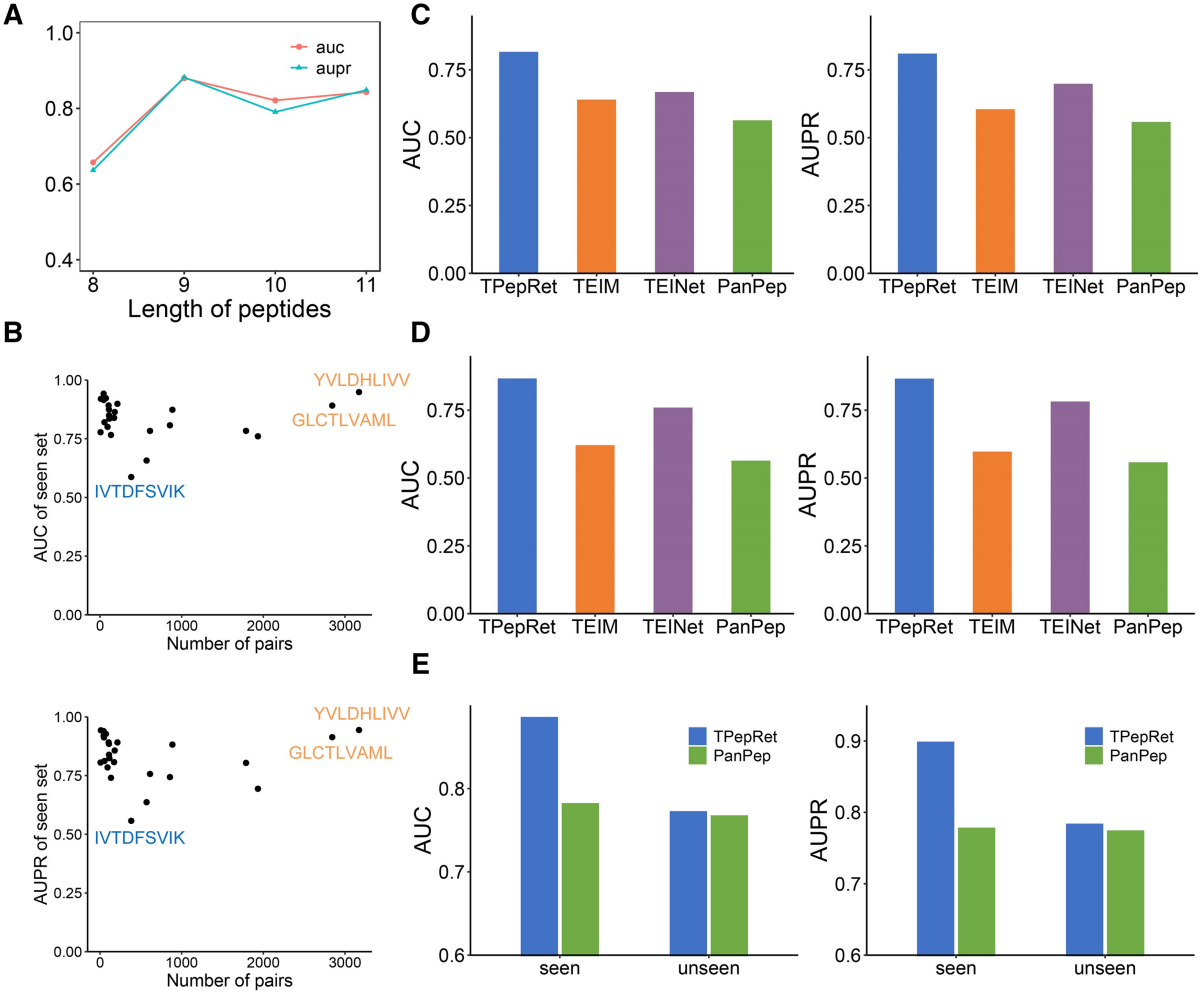
(A) The AUC and AUPR results for different peptide lengths in the seen set. (B) The relationship between AUC and AUPR results and their combined quantity. (C) Comparison of prediction results on the seen set. (D) The prediction results on the intersection seen set (on 25 intersection peptides in the training set of four tools). (E) Comparison of results after retraining and testing TPepRet using the training and testing sets of PanPep.

Firstly, the seen set is running on the tool directly. We calculate the AUC and AUPR results for different peptide lengths in the seen set, and the results in Fig. 2A show that the two indicators for peptides with lengths ranging from 9 to 11 mostly exceeded 0.8. However, when the peptide length is 8, the predicted results show a significant decrease. Next, the entries in the seen set are grouped according to different peptides, and their AUC and AUPR are calculated for scatter plot analysis with respect to the pairs number in the seen set. The scatter plots in Fig. 2B show that most peptides have few pairs, and their AUC and AUPR remain above 0.75. Two peptides with more pairs and better performance are marked in the scatter plot (YVLDHLIVV, GLCTLVAML), as well as one peptide with the worst performance (IVTDFSVIK). Finally, we run the seen set on three reference tools, the results in Fig. 2C reveal that TPepRet exhibits the best prediction performance, with an AUC of 0.8164 and an AUPR of 0.8100, followed by TEINet.

To eliminate any bias where the seen set may appear in the training set of TPepRet but not in the reference tool, we extract a total of 14 551 pairs of 25 peptide binding pairs based on the training sets of four tools. Figure 2D illustrates that, in the intersection seen set, TPepRet maintains a leading position as well, with AUC/AUPR of 0.8671 and 0.8665, respectively. This indicates the predictive advantage of TPepRet remains reliable on a completely fair test set. Supplementary Tables S2 and S3 show the performance of TPepRet’s results at a finer level of granularity.

Considering PanPep’s claim of performing well on its unseen set, we conduct a further “fair” testing scheme. PanPep is the only tool among the three reference tools that provide all positive and negative samples during training, with the data divided into seen and unseen sets. We retrain and test TPepRet using PanPep’s training and testing data. The results in Fig. 2E demonstrate that TPepRet’s model architecture achieves superior prediction performance on both the seen and unseen sets. The result on the unseen set may be attributed to PanPep using a different negative sample filling strategy, confirming the superior predictive ability of TPepRet in TCR-peptide binding.

The ProtBERT-based baseline model uses the ProtBERT protein pretraining model to encode CDR3 fragments and peptides separately. The output matrices from these encodings are then concatenated and processed by a three-layer fully connected neural network for final output. The prediction performance of the baseline model is evaluated on both the seen set and the intersection seen set. While the baseline model achieves AUC scores of 0.7792 and 0.8523 and AUPR scores of 0.7685 and 0.8401 on the seen set and the intersection seen set, respectively, TPepRet consistently provides superior results.

To verify TPepRet’s predictive ability in scenarios with limited data volume, the unseen set is further divided in a 4:1 ratio. 4/5 are placed in the training set for retraining, while the remaining 1/5 continues to be used for testing. After the data in the unseen set enters the training set, the tool’s prediction results for the remaining 1/5 of the data increase from random prediction to AUC = 0.5914 and AUPR = 0.6059. This improvement in prediction performance with the addition of a small amount of data to the model training set indicates TPepRet’s significant adaptability, promising for predicting TCR-peptide binding as the data volume increases.

### 3.2 TPepRet better characterizes peptides’ binding preferences

**Figure 3.**
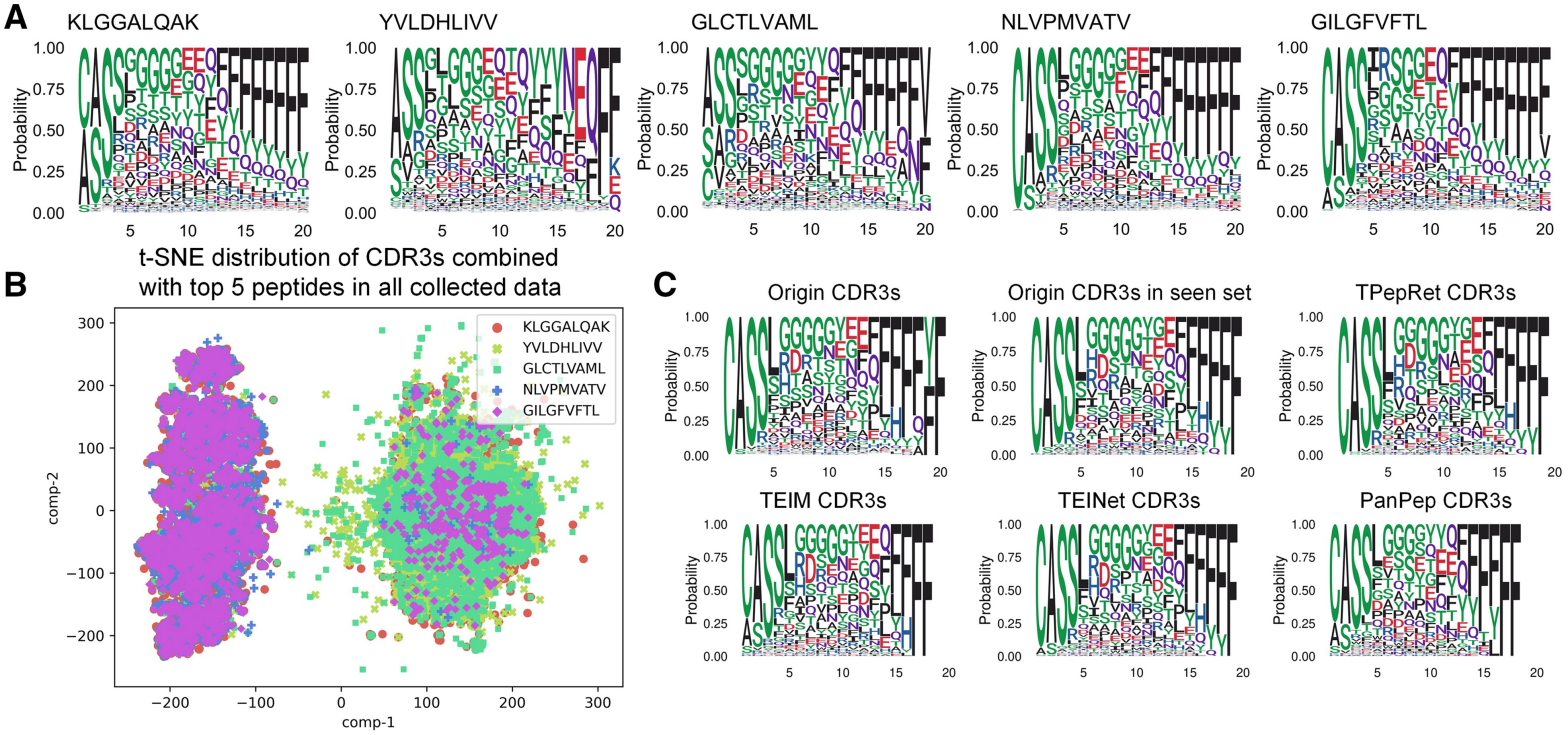
(A) Ggseqlogo of the real binding CDR3 sequences of top five peptides in all collected data. (B) The t-SNE distribution of CDR3s combined with top five peptides in all collected data. (C) The predicted ggseqlogo of the top N CDR3 sequences of TPRVTGGGAM using different tools.

To explore the binding characteristics of each peptide, we conduct a detailed analysis of the binding preferences based on existing binding data. Specifically, we extract the peptides of over 100 real binding CDR3s from all collected binding pairs. The probabilities of amino acids appearing at each site are visualized using the “ggseqlogo” package (Wagih 2017), an R toolkit capable of displaying the probability of amino acids at each site. The height of each amino acid in the visualization corresponds to the frequency of its appearance at that site in the existing binding data. A total of 99 peptides meeting the specified requirements are extracted for further analysis. Figure 3A shows a visualization example of peptides with the top five data volumes in all collected datasets (all visualization images are available in Supplementary Fig. S3). Despite the frequency deviation caused by the insufficient length of most CDR3 data at the tail end, notable observations include the tendency for peptides to exhibit strong binding preferences in the head of CDR3. In contrast, in the middle and tail of CDR3, a broader range of binding preferences is observed. Figure 3B shows the t-distributed Stochastic Neighbor Embedding (t-SNE) distribution of CDR3s combined with top five peptides in all collected data. We can see the peptides with similar ggseqlogos have similar spatial distributions (group 1: KLGGALQAK/YVLDHLIVV/GLCTLVAML, group 2: NLVPMVATV/GILGFVFTL). Group 2 has a similar binding preference, whereas group 1 exhibit a wider range of binding preferences. More details about the t-SNE process and the results of the similarity analysis of the two groups can be found in Supplementary Materials and Supplementary Fig. S4.

To assess the ability of the tools to characterize the binding preferences of peptides, we extract the top N predicted binding scores of CDR3s from the seen set and generate ggseqlogo plots for each tool. Here, *N* represents the number of real binding compounds in the seen set. As an example, Fig. 3C illustrates the case of TPRVTGGGAM (all plots are available in Supplementary Fig. S5). In the top of Fig. 3C, the first image represents the ggseqlogo maps of all the collected complexes of TPRVTGGGAM, while the second image shows the ggseqlogo maps of all positive samples in the seen set. By comparing the tendency of CDR3 to bind amino acids at sites 1/2/10/11/12/13, it becomes evident that TPepRet exhibits the best reproducibility in replicating the original binding preferences of peptides.

**Figure 4.**
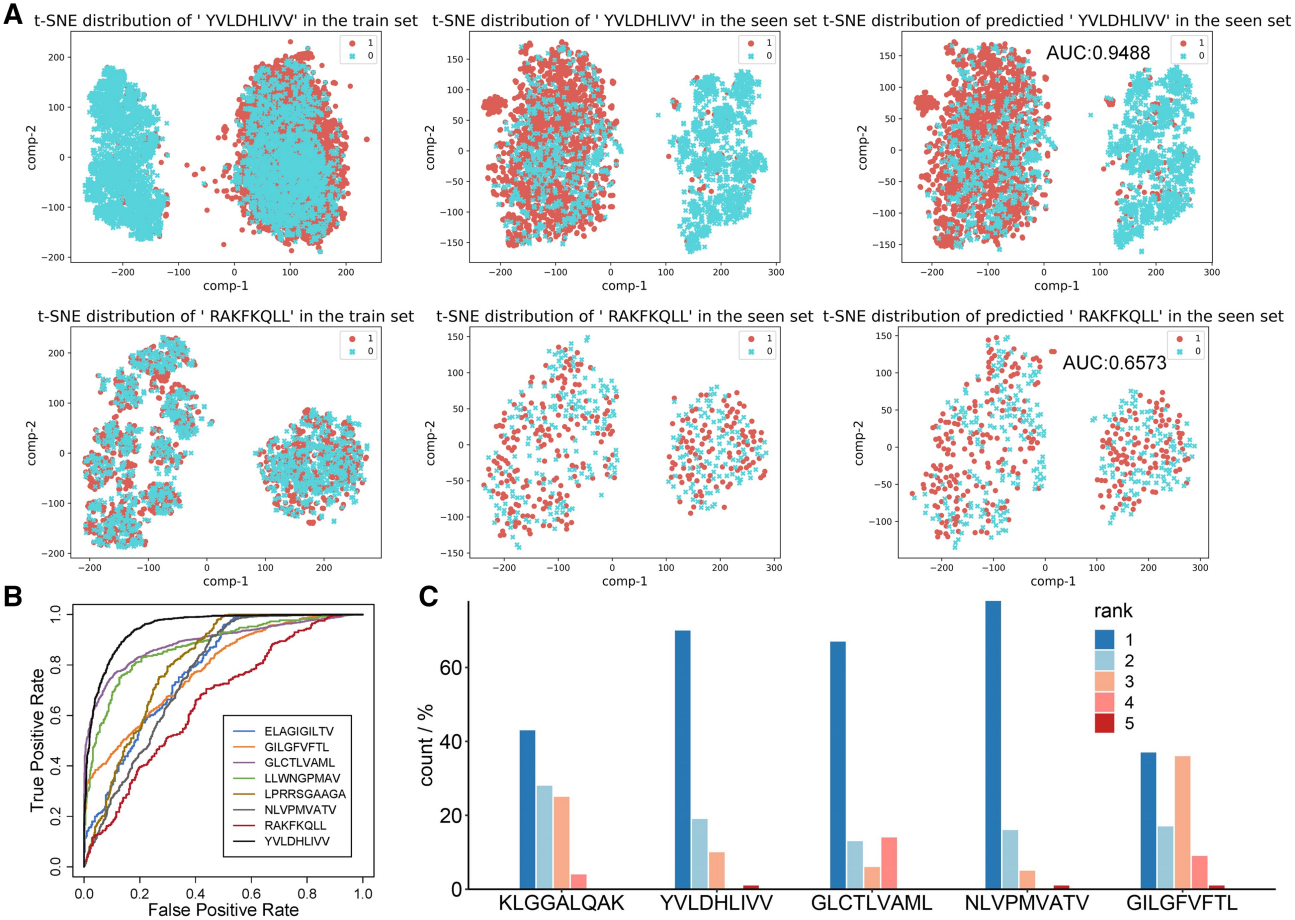
(A) The t-SNE distributions of positive and negative samples in the train set (left), seen set (middle), and predicted result set (right) of ‘YVLDHLIVV’ (top) and ‘RAKFKQLL’ (bottom). (B) The AUROC of the eight peptides with the highest number of paired instances from both the training and seen sets. (C) Pairing of five peptides with 500 binding pairs in the validation dataset, and predicting the binding scores of each pair using TPepRet. For each CDR3, rank from highest to lowest according to binding score and provide a statistical rank of the real binding compounds among these five peptides. A rank of 1 means that TPepRet correctly predict the real CDR3-peptide pairs and give the highest score.

To further dissect the discriminatory power of TPepRet, we select the eight peptides with the highest number of paired instances from both the training and seen sets. We then proceed to visualize the t-SNE distributions of positive and negative samples for each of these peptides (Fig. 4A and Supplementary Fig. S6). Figure 4B illustrates the Area Under the Receiver Operating Characteristic Curve (AUROC) for these eight peptides, revealing significant variability in predictive performance. Specifically, Fig. 4A (top) highlights the “YVLDHLIVV” set, which achieved the highest AUROC score of 0.9488 among the peptides. In contrast, Fig. 4A (bottom) depicts the “RAKFKQLL” set, which yielded the lowest AUROC score of 0.6573. A comparative analysis of these peptides’ training sets and their respective AUROC scores underscores the pivotal role of sample quality in predictive accuracy. It becomes evident that the degree of dissimilarity between positive and negative samples in the training phase significantly impacts model performance. This finding reinforces the notion that robust predictive modeling necessitates not only voluminous data but also a judicious selection of high-quality positive and negative examples to train on.

### 3.3 TPepRet can correctly retrieve the binding peptide of each CDR3

To further assess the anti-interference ability of TPepRet, a validation dataset is prepared, including five peptides with the maximum binding pairs. From each of these five peptides, 100 non-repetitive CDR3s are randomly extracted in the seen set, resulting in a total of 500 CDR3s. TPepRet is then utilized to predict the binding of each CDR3 to the five peptides. The original binding peptides of CDR3s are statistically ranked among the five peptides. In an ideal scenario, the original binding sequences of CDR3s should be ranked first. The predicted ranking of the five peptides based on their real binding CDR3s is illustrated in Fig. 4C. It is evident that all CDR3s can still recognize their original binding compounds and rank the predicted values first among pseudo binding pairs. Additionally, from Fig. 4C, it can be observed that many CDR3s also rank other peptides at higher positions. This observation is not surprising as these are the top five peptides with the most binding compounds. Their biological significance suggests strong immunogenicity, and they are likely to elicit reactions from other CDR3s as well.

### 3.4 TPepRet can characterize clonal expansion of T cells

The stronger binding ability of T cells may indicate that their higher clone expansion (Huang *et al.* 2019). Previous studies have attempted to analyze the consistency between the predicted scores of the tool and the clonal expansion rates of T cells. We have also conducted similar experiments to validate the ability of TPepRet to characterize T-cell clonal expansion. For the consistency of comparison, we use the same test data as PanPep, namely two single-cell datasets from two healthy donors on the 10x Genomics Chromium Single Cell Immune Profiling platform. The first validation is the ability on the 0–1 classification problem (0 for unique clones, 1 for expanded clones). TPepRet obtains AUC: 0.724, AUPR: 0.280 and AUC: 0.784, AUPR: 0.434 on two donors, respectively (while PanPep scores with AUC: 0.565, AUPR: 0.093 on donor 1 and AUC: 0.583, AUPR: 0.121 on donor 2, respectively). The results indicate the advantage of TPepRet in the classification of T-cell clonal expansion data. Then we validate the consistency between the predicted scores of tools and the original unique molecular identifier (UMI) count, as well as the T-cell clonal expansion rate. TPepRet achieves scores of 0.101 and 0.234 on two donors, slightly lower than PanPep’s 0.280 and 0.234, but higher than the UMI coefficients of 0.046 and 0.016. This indicates that the TPepRet score is better than the UMI coefficient in characterizing T-cell clonal expansion rate but slightly weaker than PanPep, but overall consistency is not high enough. In summary, TPepRet is superior to PanPep in characterizing the clonal expansion rate of T cells, especially in the 0–1 classification task.

### 3.5 Prediction after alanine scanning can determine the site importance

**Figure 5.**
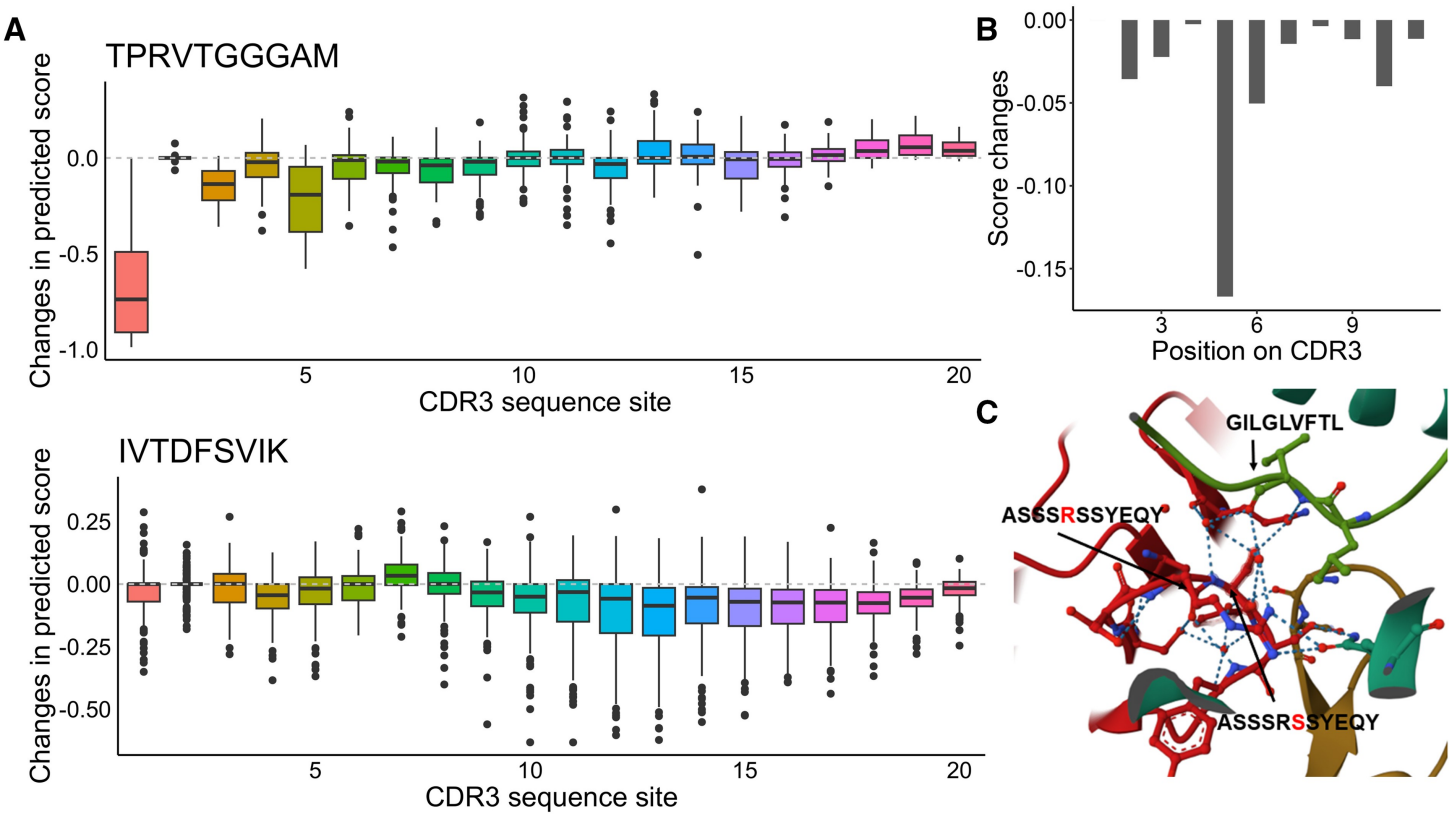
(A) Statistical distribution of the changes in predicted results after scanning for ‘alanine’ on the CDR3 sequences (TPRVTGGGAM and IVTDFSVIK as examples). (B) Changes in predicted TPepRet scores after ‘alanine scan’ on CDR3 on 5HHM. (C) The 3D crystal structure of 5HHM.

In order to investigate the impact of individual sites on the CDR3 on binding interactions, the “alanine scan” method (Weiss *et al.* 2000) is employed to systematically mutate each site on the CDR3. The resulting change in binding score between each mutated sequence and the original peptide is then predicted using TPepRet. Alanine scans are conducted on the binding data of 91 peptides, each with more than 10 CDR3 conjugates in the observed dataset. Subsequently, the predicted changes in the outcomes of these 91 mutations are obtained. The results of these 91 peptides can be generally divided into two types. One is a peptide with no binding preference for the first amino acid in the head of CDR3, such as “TPRVTGGGAM” in Fig. 5A. From the real binding logo in Fig. 3, it can be seen that it only prefers cysteine for the first amino acid of CDR3, so there is a significant change in the results after the alanine mutation. Another type is a peptide with a preference for alanine binding in the first amino acid at the head of CDR3, such as “IVTDFSVIK” in Fig. 5A. The change in binding fraction of this peptide is mainly concentrated between sites 10–13. The comprehensive results of the alanine scan reveal that the 1/3/5 positions at the head of CDR3 and the 10–13 sites in the middle rear are pivotal key sites governing the binding interaction between peptides and CDR3s. This conclusion is consistent with the previous chapters where TPepRet is able to capture the binding preferences of peptides at these key sites. Figure 5B and C show an example of peptide-TCR crystal structure extracted from the Protein Data Bank (PDB) database (ID: 5HHM) (Valkenburg *et al.* 2016). Figure 5B shows the changes in TPepRet scores after conducting an “alanine scan”; and Fig. 5C shows the 3D structure of its key contact sites. It can be seen that the TPepRet score decreases the most at the two key contact sites. TPepRet attains optimal predictive performance precisely due to its ability to discern the preferences of peptides at these crucial sites. Identifying these key sites holds significant importance in guiding clinical applications. More “alanine scan” results can be found in Supplementary Fig. S7.

### 3.6 High performance on natural and synthetic large-scale T-cell receptor databases

To assess the predictive capability of TPepRet on extensive real-world datasets, we acquire TCR data from over 1400 subjects who are exposed to or infected with the SARS-CoV-2 virus, as published by Robins *et al.* in 2020 (Nolan *et al.* 2020), totaling hundreds of millions of sequences. Following purification, weight removal, and categorization based on distinct peptide groups, a dataset comprising 1 48 181 binding instances across 325 peptide groups is assembled. The Spearman coefficient is computed between the maximum binding probability of each peptide group and its hit count to assess the correlation between the binding probability predicted by TPepRet and its expansion probability. The results demonstrate a Spearman correlation coefficient of 0.64, signifying a substantial correlation between binding probability and expansion of peptide groups. This finding affirms that the output of TPepRet serves not only as a tool to ascertain the binding occurrence between peptides and TCRs but also mirrors the strength of their binding. Consequently, TPepRet is poised to offer robust support for clinical treatment.

### 3.7 TPepRet can screen SARS-CoV-2 TCRs

We have collected an additional SARS-CoV-2-related TCR data from the IEDB database, totaling 337 data. TPepRet shows excellent screening performance on this dataset. There are 171 entries with a predicted score of over 0.99, 242 entries with a predicted score of over 0.9, 281 entries with a predicted score of over 0.7, and 305 entries with a predicted score of over 0.5. This result demonstrates TPepRet’s extremely strong SARS-CoV-2 TCR molecular screening ability, which is very useful for virus screening in the clinic. More detailed results can be found in Supplementary Table S4.

## 4 Conclusions

With the burgeoning volume of data, computational methods now offer valuable support for clinical immunotherapy. Nevertheless, the current tools exhibit inadequate predictive performance. TPepRet integrates insights from prior tools and employs a fusion of BiGRU and RetNet modules for its modeling. Following comparative analysis, TPepRet has emerged as the preeminent tool for predicting TCR-peptide binding. This article illustrates the strengths of TPepRet by evaluating its predictive performance in three distinct environments. Additionally, TPepRet assesses its usability by characterizing peptide binding preferences, distinguishing authentic binding compounds from spurious ones, characterizing the T-cell clonal expansion rate, evaluating the impact of alanine mutations on prediction results, and gauging performance on large external databases. Finally, the ability of TPepRet to screen SARS-CoV-2 TCRs is also demonstrated. TPepRet offers a more comprehensive characterization of the binding dynamics between peptides and TCR, delivering precise and visualized support for clinical applications.

While TPepRet has demonstrated superior predictive results, opportunities for enhancement persist. As the dataset size expands and data quality improves, TPepRet’s predictive performance is expected to improve. TPepRet exclusively focuses on the CDR3 fragment on the TCR β chain, recognizing it as the crucial binding element. However, certain fragments with potential roles have been overlooked. As the dataset expands, incorporating additional potentially relevant data into the model may yield improved results. Owing to the constrained availability of structural data, TPepRet currently provides limited fine-grained residue-level prediction information. We anticipate that the ongoing emergence of high-quality and fine-grained data will contribute to a more refined modeling of TCR-peptide binding prediction.

## Author contributions

M.W. and M.L. conceived the study and designed the experiments. M.W. developed the algorithm and optimized the analytic method. M.W. and W.F. conducted the data analysis. M.W., W.F., T.W., and M.L contributed to the interpretation of the data and wrote the paper. All authors read and approved the final manuscript.

## Supplementary Material

btaf022_Supplementary_Data

## Data Availability

The data underlying this article are publicly available with their sources described in the article.
