# Supplementary material for: TPepRet: a deep learning model for characterizing T-cell receptors–antigen binding patterns
Source: Bioinformatics. 2025 Jan 28;41(1):btaf022. doi: 10.1093/bioinformatics/btaf022 (PMC11784750; doi:10.1093/bioinformatics/btaf022)

# TPepRet: A deep learning model for characterizing T cell receptors-antigen binding patterns

Meng Wang^1^, Wei Fan^2^, Tianrui Wu^1^, Min Li^1,*^

1 School of Computer Science and engineering, Central South University, Changsha 410083, China.

2 Nuffield Department of Women’s and Reproductive Health, University of Oxford, Oxford, OX39DU, UK.

*To whom correspondence should be addressed.

The data source and their overlap are shown in Supplement Figure S1A, there is a small amount of overlap between the data from VDJdb and from McPAS, and there is no overlap between the data from the IEDB database and the other two repositories. All data are originated from humans. We define the concepts of seen set and unseen set in our test set. Seen data means that all of the peptides in it appear in the training data, while none of the sequences in unseen data appear in the training data. The significance of seen data is that the model is learning the binding relationships of the peptides in it, whereas unseen data is completely new binding data for the model. The relationship between them is shown in Supplement Figure S1B.

The procedure for t-SNE analysis in the main text: the CDR sequences of individual peptides are padded to a given length and then encoded with AAIndex, followed by flattening all dimensions of all individual CDR sequences into a one-dimensional vector. The five peptides with the highest number of bound CDRs are subjected to TSNE classification and visualization. The features of the CDRs will be initialized to the data with principal component analysis (PCA), which speeds up the convergence of t-SNE. By mapping each CDR sequence into the same two-dimensional space, similar relationships between different classes (combining different peptides) can be seen.

Details of model parameters: the CDR3 fragments and peptides are encoded into a 20 × 28 matrix and a 14 × 28 matrix, respectively, using AAIndex, and then the two matrices are fed into the BiGRU network containing 128 hidden layers to learn the relationship in the sequence direction of each sequence, and subsequence, respectively. After BiGRU, the two matrices become 20 × 256 and 14 × 256, respectively. Subsequently, the two matrices are spliced in the second dimension to obtain a 34 × 256 matrix. The matrices are then passed into a RetNet network with parameters layers=24, hidden_dim=34×256, ffn_size=512, heads=8, and double_v_dim=True, respectively. The output of this network will still be a 34×256 matrix. The matrix is then sent to the (34 × 256) × 128 fully connected layer, ReLu function and the 128 × 128 fully connected layer, ReLu function. Then after a 20% random weight removal, it goes to the final 128 × 1 fully connected layer that has a Sigmoid activation function that is used to derive the predicted probability of binding and maps between 0 and 1.

we have performed ablation experiments on several key modules of TPepRet, namely, without RetNet, without BiGRU, with GRU module, and using a different number of RetNet layers (the recommended number of layers is 24 for TPepRet in this paper). It turns out that TPepRet achieves optimal prediction results on both AUC and AUPR. The results are shown in Supplement Figure S2.

To quantify the representation of the two groups in Section 3.2 in the main text, we added a heat map to illustrate this result, as shown in Supplementary Figure S4. As can be seen from the Heat Map, there is a similarity between the binders of NLVPMVATV and GILGFVFTL, and the similarity between the remaining three is not significant. This result corresponds to Figure 3A in the main text, where it is observed that NLVPMVATV has a similar binding preference to GILGFVFTL, whereas the other three exhibit a wider range of binding preferences.

**Supplement Table S1. The selected entries in AAIndex.**

| AA | Ala | Arg | Asn | Asp | Cys | Gln | Glu | Gly | His | Ile | Leu | Lys | Met | Phe | Pro | Ser | Thr | Trp | Tyr | Val |
| --- | --- | --- | --- | --- | --- | --- | --- | --- | --- | --- | --- | --- | --- | --- | --- | --- | --- | --- | --- | --- |
| AACode | A | R | N | D | C | Q | E | G | H | I | L | K | M | F | P | S | T | W | Y | V |
| CHOP780202 | 0.83 | 0.93 | 0.89 | 0.54 | 1.19 | 1.1 | 0.37 | 0.75 | 0.87 | 1.6 | 1.3 | 0.74 | 1.05 | 1.38 | 0.55 | 0.75 | 1.19 | 1.37 | 1.47 | 1.7 |
| CIDH920103 | 0.36 | -0.52 | -0.9 | -1.09 | 0.7 | -1.05 | -0.83 | -0.82 | 0.16 | 2.17 | 1.18 | -0.56 | 1.21 | 1.01 | -0.06 | -0.6 | -1.2 | 1.31 | 1.05 | 1.21 |
| CIDH920105 | 0.02 | -0.42 | -0.77 | -1.04 | 0.77 | -1.1 | -1.14 | -0.8 | 0.26 | 1.81 | 1.14 | -0.41 | 1 | 1.35 | -0.09 | -0.97 | -0.77 | 1.71 | 1.11 | 1.13 |
| FAUJ880109 | 0 | 4 | 2 | 1 | 0 | 2 | 1 | 0 | 1 | 0 | 0 | 2 | 0 | 0 | 0 | 1 | 1 | 1 | 1 | 0 |
| FAUJ880111 | 0 | 1 | 0 | 0 | 0 | 0 | 0 | 0 | 1 | 0 | 0 | 1 | 0 | 0 | 0 | 0 | 0 | 0 | 0 | 0 |
| FINA910104 | 1 | 1.7 | 1 | 0.7 | 1 | 1 | 0.7 | 1.5 | 1 | 1 | 1 | 1.7 | 1 | 1 | 0.1 | 1 | 1 | 1 | 1 | 1 |
| GEIM800104 | 1.19 | 1 | 0.94 | 1.07 | 0.95 | 1.32 | 1.64 | 0.6 | 1.03 | 1.12 | 1.18 | 1.27 | 1.49 | 1.02 | 0.68 | 0.81 | 0.85 | 1.18 | 0.77 | 0.74 |
| GEIM800106 | 0.86 | 1.15 | 0.6 | 0.66 | 0.91 | 1.11 | 0.37 | 0.86 | 1.07 | 1.17 | 1.28 | 1.01 | 1.15 | 1.34 | 0.61 | 0.91 | 1.14 | 1.13 | 1.37 | 1.31 |
| KANM800102 | 0.81 | 0.85 | 0.62 | 0.71 | 1.17 | 0.98 | 0.53 | 0.88 | 0.92 | 1.48 | 1.24 | 0.77 | 1.05 | 1.2 | 0.61 | 0.92 | 1.18 | 1.18 | 1.23 | 1.66 |
| KLEP840101 | 0 | 1 | 0 | -1 | 0 | 0 | -1 | 0 | 0 | 0 | 0 | 1 | 0 | 0 | 0 | 0 | 0 | 0 | 0 | 0 |
| KRIW710101 | 4.6 | 6.5 | 5.9 | 5.7 | -1 | 6.1 | 5.6 | 7.6 | 4.5 | 2.6 | 3.25 | 7.9 | 1.4 | 3.2 | 7 | 5.25 | 4.8 | 4 | 4.35 | 3.4 |
| LIFS790101 | 0.92 | 0.93 | 0.6 | 0.48 | 1.16 | 0.95 | 0.61 | 0.61 | 0.93 | 1.81 | 1.3 | 0.7 | 1.19 | 1.25 | 0.4 | 0.82 | 1.12 | 1.54 | 1.53 | 1.81 |
| MEEJ800101 | 0.5 | 0.8 | 0.8 | -8.2 | -6.8 | -4.8 | -16.9 | 0 | -3.5 | 13.9 | 8.8 | 0.1 | 4.8 | 13.2 | 6.1 | 1.2 | 2.7 | 14.9 | 6.1 | 2.7 |
| OOBM770102 | -1.4 | -0.92 | -1.18 | -1.16 | -1.37 | -1.12 | -1.16 | -1.36 | -1.22 | -1.19 | -1.32 | -1.07 | -1.3 | -1.14 | -1.24 | -1.3 | -1.25 | -1.03 | -1.03 | -1.25 |
| PALJ810107 | 1.08 | 0.93 | 1.05 | 0.86 | 1.22 | 0.95 | 1.09 | 0.85 | 1.02 | 0.98 | 1.04 | 1.01 | 1.11 | 0.96 | 0.91 | 0.95 | 1.15 | 1.17 | 0.8 | 1.03 |
| QIAN880123 | -0.44 | -0.13 | 0.05 | -0.2 | 0.13 | -0.58 | -0.28 | 0.08 | 0.09 | -0.04 | -0.12 | -0.33 | -0.21 | -0.13 | -0.48 | 0.27 | 0.47 | -0.22 | -0.11 | 0.06 |
| RACS770103 | 1.16 | 1.72 | 1.97 | 2.66 | 0.5 | 3.87 | 2.4 | 1.63 | 0.86 | 0.57 | 0.51 | 3.9 | 0.4 | 0.43 | 2.04 | 1.61 | 1.48 | 0.75 | 1.72 | 0.59 |
| RADA880108 | -0.06 | -0.84 | -0.48 | -0.8 | 1.36 | -0.73 | -0.77 | -0.41 | 0.49 | 1.31 | 1.21 | -1.18 | 1.27 | 1.27 | 0 | -0.5 | -0.27 | 0.88 | 0.33 | 1.09 |
| ROSM880102 | -0.67 | 3.89 | 2.27 | 1.57 | -2 | 2.12 | 1.78 | 0 | 1.09 | -3.02 | -3.02 | 2.46 | -1.67 | -3.24 | -1.75 | 0.1 | -0.42 | -2.86 | 0.98 | -2.18 |
| SWER830101 | -0.4 | -0.59 | -0.92 | -1.31 | 0.17 | -0.91 | -1.22 | -0.67 | -0.64 | 1.25 | 1.22 | -0.67 | 1.02 | 1.92 | -0.49 | -0.55 | -0.28 | 0.5 | 1.67 | 0.91 |
| ZIMJ680102 | 11.5 | 14.3 | 12.8 | 11.7 | 13.5 | 14.5 | 13.6 | 3.4 | 13.7 | 21.4 | 21.4 | 15.7 | 16.3 | 19.8 | 17.4 | 9.47 | 15.8 | 21.7 | 18 | 21.6 |
| ZIMJ680104 | 6 | 10.8 | 5.41 | 2.77 | 5.05 | 5.65 | 3.22 | 5.97 | 7.59 | 6.02 | 5.98 | 9.74 | 5.74 | 5.48 | 6.3 | 5.68 | 5.66 | 5.89 | 5.66 | 5.96 |
| AURR980120 | 0.71 | 1.09 | 0.95 | 1.43 | 0.65 | 0.87 | 1.19 | 1.07 | 1.13 | 1.05 | 0.84 | 1.1 | 0.8 | 0.95 | 1.7 | 0.65 | 0.09 | 1.25 | 0.85 | 1.12 |
| MUNV940103 | 1.08 | 0.98 | 1.2 | 1.27 | 0.73 | 1.05 | 1.09 | 1.1 | 0.91 | 0.58 | 0.79 | 1.03 | 0.81 | 0.69 | 1.41 | 0.99 | 0.78 | 0.76 | 0.67 | 0.55 |
| NADH010104 | 32 | -95 | -73 | -29 | 182 | -95 | -74 | -22 | -25 | 106 | 104 | -124 | 82 | 132 | -82 | -34 | 20 | 118 | 44 | 113 |
| NADH010106 | 5 | -57 | -77 | 45 | 224 | -67 | -8 | -47 | -50 | 83 | 82 | -38 | 83 | 117 | -103 | -41 | 79 | 130 | 27 | 117 |
| GUYH850105 | -0.27 | 2 | 0.61 | 0.5 | -0.23 | 1 | 0.33 | -0.22 | 0.37 | -0.8 | -0.44 | 1.17 | -0.31 | -0.55 | 0.36 | 0.17 | 0.18 | 0.05 | 0.48 | -0.65 |
| MIYS990104 | -0.04 | 0.07 | 0.13 | 0.19 | -0.38 | 0.14 | 0.23 | 0.09 | -0.04 | -0.34 | -0.37 | 0.33 | -0.3 | -0.38 | 0.19 | 0.12 | 0.03 | -0.33 | -0.29 | -0.29 |

**Supplement Table S2. AUC/AUPR results for TPepRet on different peptide lengths in the intersection seen data.**

| Peplen | AUC | AUPR |
| --- | --- | --- |
| 8 | 0.6573 | 0.6366 |
| 9 | 0.8800 | 0.8821 |
| 10 | 0.8212 | 0.7906 |
| 11 | 0.8431 | 0.8484 |

**Supplement Table S3. AUC/AUPR results for TPepRet on peptides with more than 100 binders.**

| Peptide | Count | AUC | AUPR |
| --- | --- | --- | --- |
| YVLDHLIVV | 3172 | 0.9488 | 0.9440 |
| GLCTLVAML | 2844 | 0.8913 | 0.9135 |
| NLVPMVATV | 1934 | 0.7606 | 0.6937 |
| GILGFVFTL | 1788 | 0.7836 | 0.8039 |
| LLWNGPMAV | 886 | 0.8735 | 0.8819 |
| LPRRSGAAGA | 856 | 0.8074 | 0.7435 |
| ELAGIGILTV | 612 | 0.7834 | 0.7569 |
| RAKFKQLL | 570 | 0.6573 | 0.6366 |
| IVTDFSVIK | 382 | 0.5870 | 0.5573 |
| KRWIILGLNK | 213 | 0.8989 | 0.8911 |
| TPRVTGGGAM | 180 | 0.8638 | 0.8568 |
| CRVLCCYVL | 172 | 0.8388 | 0.8072 |
| CINGVCWTV | 134 | 0.7661 | 0.7405 |
| EAAGIGILTV | 114 | 0.8356 | 0.8241 |
| VTEHDTLLY | 110 | 0.8496 | 0.8387 |
| KAFSPEVIPMF | 109 | 0.8747 | 0.8849 |
| FRCPRRFCF | 105 | 0.8919 | 0.8913 |

**Supplement Table S4. A statistical rank of the predicted binding compounds among the 5 peptides with the most conjugates.**

| Peptide | Rank | Count |
| --- | --- | --- |
| KLGGALQAK | 1 | 43 |
| KLGGALQAK | 2 | 28 |
| KLGGALQAK | 3 | 25 |
| KLGGALQAK | 4 | 4 |
| KLGGALQAK | 5 | 0 |
| YVLDHLIVV | 1 | 70 |
| YVLDHLIVV | 2 | 19 |
| YVLDHLIVV | 3 | 10 |
| YVLDHLIVV | 4 | 0 |
| YVLDHLIVV | 5 | 1 |
| GLCTLVAML | 1 | 67 |
| GLCTLVAML | 2 | 13 |
| GLCTLVAML | 3 | 6 |
| GLCTLVAML | 4 | 14 |
| GLCTLVAML | 5 | 0 |
| NLVPMVATV | 1 | 78 |
| NLVPMVATV | 2 | 16 |
| NLVPMVATV | 3 | 5 |
| NLVPMVATV | 4 | 0 |
| NLVPMVATV | 5 | 1 |
| GILGFVFTL | 1 | 37 |
| GILGFVFTL | 2 | 17 |
| GILGFVFTL | 3 | 36 |
| GILGFVFTL | 4 | 9 |
| GILGFVFTL | 5 | 1 |

**Supplement Table S5. Predictive Score for TPepRet on SARS-CoV-2 TCRs.**

| SARS-CoV-2-related CDR3 | Peptide | Score |
| --- | --- | --- |
| ASGEGNTGELF | YLQPRTFLL | 0.9959 |
| ASGGTNTGELF | YLQPRTFLL | 0.9828 |
| ASSDLDTGELF | YLQPRTFLL | 0.9970 |
| ASSDIDTGELF | YLQPRTFLL | 0.9970 |
| ASSELAGGNTGELF | YLQPRTFLL | 0.9993 |
| ASSDLNTGELF | YLQPRTFLL | 0.9991 |
| ASSDMNTGELF | YLQPRTFLL | 0.9985 |
| ASSLDIEAF | YLQPRTFLL | 0.9376 |
| ASSPDIEAF | YLQPRTFLL | 0.9996 |
| ASSPDIEQF | YLQPRTFLL | 0.9870 |
| ASSPDIEQY | YLQPRTFLL | 0.9953 |
| ASSPEDTQY | YLQPRTFLL | 0.6404 |
| ASSSDIEAF | YLQPRTFLL | 0.9948 |
| ASSSQNTGELF | YLQPRTFLL | 0.9416 |
| ASSSLNTGELF | YLQPRTFLL | 0.9674 |
| ASTPLNTGELF | YLQPRTFLL | 0.9936 |
| ASTDLNTGELF | YLQPRTFLL | 0.9960 |
| ATSNLNTGELF | YLQPRTFLL | 0.9930 |
| SARDGLAMNTGELF | YLQPRTFLL | 0.9997 |
| SARDGLAQNTGELF | YLQPRTFLL | 0.9993 |
| SVEEDRNTGELF | YLQPRTFLL | 0.9855 |
| AIQYANTGELF | YLQPRTFLL | 0.9997 |
| ASQGLNTGELF | YLQPRTFLL | 0.9671 |
| ATGGLNTGELF | YLQPRTFLL | 0.9978 |
| ATHELNTGELF | YLQPRTFLL | 0.9991 |
| ATMPDRNTGELF | YLQPRTFLL | 0.9430 |
| ATQAVNTGELF | YLQPRTFLL | 0.9965 |
| ATQDANTGELF | YLQPRTFLL | 0.9998 |
| ATQRTNTGELF | YLQPRTFLL | 0.9848 |
| ATSDLDSGELF | YLQPRTFLL | 0.9970 |
| ATSGAETHNTGELF | YLQPRTFLL | 0.8878 |
| ATTDLNTGELF | YLQPRTFLL | 0.9975 |
| ASSPPGAGNTGELF | YLQPRTFLL | 0.9145 |
| ASSPPGGGNTGELF | YLQPRTFLL | 0.9644 |
| AAQATNTGELF | YLQPRTFLL | 0.9986 |
| AAQMTNTGELF | YLQPRTFLL | 0.9981 |
| AGQNLNTGELF | YLQPRTFLL | 0.9987 |
| AGQRANTGELF | YLQPRTFLL | 0.9990 |
| ASQATNTGELF | YLQPRTFLL | 0.9923 |
| ASQDLNTGELF | YLQPRTFLL | 0.9997 |
| ASQDQNTGELF | YLQPRTFLL | 0.9986 |
| ASQEANTGELF | YLQPRTFLL | 0.9994 |
| ASQQANTGELF | YLQPRTFLL | 0.9957 |
| ASSERNSLEAF | YLQPRTFLL | 0.7856 |
| ASSFLAGGNTGELF | YLQPRTFLL | 0.8332 |
| ASSIIGGGNTGELF | YLQPRTFLL | 0.7778 |
| ATQGWDTGELF | YLQPRTFLL | 0.9239 |
| ATQNLNTGELF | YLQPRTFLL | 0.9982 |
| AYQEVNTGELF | YLQPRTFLL | 0.9997 |
| AARDQRDLNTGELF | YLQPRTFLL | 1.0000 |
| AIRTSGMLNTGELF | YLQPRTFLL | 0.9958 |
| ALLLDWDTGELF | YLQPRTFLL | 0.9336 |
| ALWKGGRENTGELF | YLQPRTFLL | 0.9963 |
| ARERDRNTGELF | YLQPRTFLL | 0.9781 |
| ASGDLDSIEAF | YLQPRTFLL | 0.9840 |
| ASGDLSSGEQY | YLQPRTFLL | 0.8287 |
| ASMDQNTGELF | YLQPRTFLL | 0.9959 |
| ASPEANTGELF | YLQPRTFLL | 0.9990 |
| ASRDIHQQNTGELF | YLQPRTFLL | 0.9998 |
| ASRGGAELNTGELF | YLQPRTFLL | 0.9827 |
| ASRGLAEQNTGELF | YLQPRTFLL | 0.9856 |
| ASRRGFEQY | YLQPRTFLL | 0.0576 |
| ASRSRVEQNTGELF | YLQPRTFLL | 0.9973 |
| ASSDLDGGEAF | YLQPRTFLL | 0.7252 |
| ASSDLDNLVAF | YLQPRTFLL | 0.9917 |
| ASSDLNSGEQY | YLQPRTFLL | 0.9371 |
| ASSDLSTGELF | YLQPRTFLL | 0.9970 |
| ASSDLTGGEAF | YLQPRTFLL | 0.7182 |
| ASSDSEGHNTGELF | YLQPRTFLL | 0.9953 |
| ASSDTTRQNTGELF | YLQPRTFLL | 0.9990 |
| ASSEIGGGNTGELF | YLQPRTFLL | 0.9952 |
| ASSEMNTGELF | YLQPRTFLL | 0.9901 |
| ASTDLDTGELF | YLQPRTFLL | 0.9818 |
| ASTGLNTGELF | YLQPRTFLL | 0.9911 |
| ASTSLNTGELF | YLQPRTFLL | 0.9666 |
| ATLAEMNTGELF | YLQPRTFLL | 0.9966 |
| ATMDDLNSGELF | YLQPRTFLL | 0.9885 |
| ATQGPSEINTGELF | YLQPRTFLL | 0.9954 |
| SAEPGTSGRDDLTQY | YLQPRTFLL | 0.5697 |
| SAGDRNTGELF | YLQPRTFLL | 0.9969 |
| SAGQRNTGELF | YLQPRTFLL | 0.9549 |
| SAHGDLNTGELF | YLQPRTFLL | 0.9940 |
| SARDAEGQNTGELF | YLQPRTFLL | 0.9998 |
| SARDAVAANTGELF | YLQPRTFLL | 0.9998 |
| SARDDQAVNTGELF | YLQPRTFLL | 0.9998 |
| SARDDRAQNTGELF | YLQPRTFLL | 0.9998 |
| SARDEAGQNTGELF | YLQPRTFLL | 1.0000 |
| SARDERAVNTGELF | YLQPRTFLL | 0.9989 |
| SARDEVAHNTGELF | YLQPRTFLL | 0.9999 |
| SARDFNSWNTGELF | YLQPRTFLL | 1.0000 |
| SARDGPGVNTGELF | YLQPRTFLL | 0.9975 |
| SARDGQGMNTGELF | YLQPRTFLL | 0.9983 |
| SARDGTGWNTGELF | YLQPRTFLL | 0.9943 |
| SARDGWDHNTGELF | YLQPRTFLL | 0.9984 |
| SARDHKAMNTGELF | YLQPRTFLL | 0.9906 |
| SARDHRGANTGELF | YLQPRTFLL | 0.9891 |
| SARDLAAQNTGELF | YLQPRTFLL | 0.9999 |
| SARDLAGENTGELF | YLQPRTFLL | 0.9982 |
| SARDLGRGWNTGELF | YLQPRTFLL | 0.9990 |
| SARDPPRVNTGELF | YLQPRTFLL | 0.9998 |
| SARDQAAQNTGELF | YLQPRTFLL | 0.9997 |
| SARDQPGQNTGELF | YLQPRTFLL | 0.9999 |
| SARDQWAANTGELF | YLQPRTFLL | 0.9998 |
| SARDQYRMNTGELF | YLQPRTFLL | 0.9996 |
| SARDRLAQNTGELF | YLQPRTFLL | 0.9999 |
| SARDSKGNGWNTGELF | YLQPRTFLL | 0.7000 |
| SARDSRAQNTGELF | YLQPRTFLL | 0.9993 |
| SARDSVAVNTGELF | YLQPRTFLL | 0.9998 |
| SARDVEGMNTGELF | YLQPRTFLL | 0.9993 |
| SARDWEQMNTGELF | YLQPRTFLL | 0.9996 |
| SARDYRQTNTGELF | YLQPRTFLL | 0.9999 |
| SARGELAVNTGELF | YLQPRTFLL | 0.9990 |
| SARGESDQNTGELF | YLQPRTFLL | 0.9991 |
| SARGGLSQNTGELF | YLQPRTFLL | 0.9945 |
| SARGGQGQNTGELF | YLQPRTFLL | 0.9943 |
| SARGLAGSNTGELF | YLQPRTFLL | 0.8330 |
| SARGWGNEANTGELF | YLQPRTFLL | 0.7790 |
| SARSRQGQNTGELF | YLQPRTFLL | 0.9413 |
| SARTDHNTGELF | YLQPRTFLL | 0.7576 |
| SARTGDDWNTGELF | YLQPRTFLL | 0.9898 |
| SARVGERAVNTGELF | YLQPRTFLL | 0.8538 |
| SVGTLNTGELF | YLQPRTFLL | 0.9500 |
| ATEYTNTGELF | YLQPRTFLL | 0.9954 |
| ATSEENTGELF | YLQPRTFLL | 0.9896 |
| AITDLNTGELF | YLQPRTFLL | 0.9991 |
| AAAERNTGELF | YLQPRTFLL | 0.9996 |
| ANQDRTSGELF | YLQPRTFLL | 0.9758 |
| ASGTLNSLVAF | YLQPRTFLL | 0.9993 |
| ASMTHNTGELF | YLQPRTFLL | 0.9922 |
| ASSDQNGNIQY | YLQPRTFLL | 0.5493 |
| ASSGTNQNIQY | YLQPRTFLL | 0.1148 |
| ASTDMNTGELF | YLQPRTFLL | 0.9918 |
| SGWQPQH | YLQPRTFLL | 0.0019 |
| SVAGFLAVYNEQF | YLQPRTFLL | 0.1734 |
| SVDADHNTGELF | YLQPRTFLL | 0.9825 |
| SVEEQNTGELF | YLQPRTFLL | 0.9955 |
| SVGDRNNLELF | YLQPRTFLL | 0.7413 |
| SVLEMNTGELF | YLQPRTFLL | 0.9973 |
| ANRDTDTQY | YLQPRTFLL | 0.4153 |
| ASQYSNTGELF | YLQPRTFLL | 0.9942 |
| ASSPLAGGNTGELF | YLQPRTFLL | 0.9941 |
| ASSPLGGGNTGELF | YLQPRTFLL | 0.9782 |
| ASSQLAGGNTGELF | YLQPRTFLL | 0.9928 |
| ASSQRNDLKAF | YLQPRTFLL | 0.8372 |
| ASSTPRKEWGRAEAF | YLQPRTFLL | 0.3352 |
| ACQELNTGELF | YLQPRTFLL | 0.9890 |
| ALFRDRNTGELF | YLQPRTFLL | 0.9106 |
| AWSDHNSKELF | YLQPRTFLL | 0.9584 |
| AWSLAGMGETQY | YLQPRTFLL | 0.2643 |
| AWSLLNDLEAF | YLQPRTFLL | 0.9841 |
| AWSVLAGGNTGELF | YLQPRTFLL | 0.9843 |
| AWSVVTDEHNEQF | YLQPRTFLL | 0.1944 |
| ASRAANEQF | FIAGLIAIV | 0.1318 |
| ASSESNTGELF | YLQPRTFLL | 0.9916 |
| ASYSGRALTEAF | YLQPRTFLL | 0.2188 |
| ASIGLNTGELF | YLQPRTFLL | 0.9931 |
| ASNELNTGELF | YLQPRTFLL | 0.9983 |
| AVLAEQNTGELF | YLQPRTFLL | 0.9988 |
| AIQESNTGELF | YLQPRTFLL | 0.9996 |
| AMQEMNTGELF | YLQPRTFLL | 0.9998 |
| ATQGLNTGELF | YLQPRTFLL | 0.9964 |
| ASSLALANEQF | YLQPRTFLL | 0.1968 |
| AASDPNTGELF | YLQPRTFLL | 0.9997 |
| AGGRGNTGELF | YLQPRTFLL | 0.9962 |
| ANGKANTGELF | YLQPRTFLL | 0.9993 |
| ANQDANTGELF | YLQPRTFLL | 0.9999 |
| ANQDSNTGELF | YLQPRTFLL | 0.9996 |
| APTPDQNTGELF | YLQPRTFLL | 0.8347 |
| ARQDTNTGELF | YLQPRTFLL | 0.9989 |
| ASGEANTGELF | YLQPRTFLL | 0.9996 |
| ASGTANTGELF | YLQPRTFLL | 0.9998 |
| ASIDENTGELF | YLQPRTFLL | 0.9985 |
| ASNSQNTGELF | YLQPRTFLL | 0.9710 |
| ASQDSNTGELF | YLQPRTFLL | 0.9993 |
| ASQNENTGELF | YLQPRTFLL | 0.9960 |
| ATLVDLNTGELF | YLQPRTFLL | 0.9851 |
| ATTGNWNTGELF | YLQPRTFLL | 0.8557 |
| AVGEANTGELF | YLQPRTFLL | 0.9999 |
| AIQDLNTGELF | YLQPRTFLL | 0.9997 |
| ASSLVAGGNTGELF | YLQPRTFLL | 0.9359 |
| ASSLVQGGNTGELF | YLQPRTFLL | 0.9458 |
| ASSPLNGGNTGELF | YLQPRTFLL | 0.9982 |
| ATQDRNTGELF | YLQPRTFLL | 0.9970 |
| AAQNLNTGELF | YLQPRTFLL | 0.9992 |
| ASSDSLDGYT | YLQPRTFLL | 0.9244 |
| ASSEANTGELF | YLQPRTFLL | 0.9972 |
| ASSEGLGGEQY | YLQPRTFLL | 0.4911 |
| ASSLDIETY | YLQPRTFLL | 0.4609 |
| ASSLDILAF | YLQPRTFLL | 0.8824 |
| ASSLDSEQF | YLQPRTFLL | 0.2389 |
| ASSLDVEQY | YLQPRTFLL | 0.7676 |
| ASSLEIEAF | YLQPRTFLL | 0.8031 |
| ASSLGLYGEQY | YLQPRTFLL | 0.2227 |
| ASSLGTGGDLH | YLQPRTFLL | 0.2453 |
| ASSNANTGELF | YLQPRTFLL | 0.9953 |
| ASSNSLGGYT | YLQPRTFLL | 0.6032 |
| ASSPDGEQY | YLQPRTFLL | 0.5050 |
| ASSPDIACT | YLQPRTFLL | 0.9857 |
| ASSPDIEDF | YLQPRTFLL | 0.9082 |
| ASSPDIQAF | YLQPRTFLL | 0.9933 |
| ASSPDITQY | YLQPRTFLL | 0.9903 |
| ASSPDIVAF | YLQPRTFLL | 0.9310 |
| ASSPDSEQY | YLQPRTFLL | 0.6739 |
| ASSPEIEAF | YLQPRTFLL | 0.8544 |
| ASSQDIEAF | YLQPRTFLL | 0.9987 |
| ASSQDIEQY | YLQPRTFLL | 0.9703 |
| ASSSDIEQF | YLQPRTFLL | 0.9348 |
| ASSSDIQQF | YLQPRTFLL | 0.9051 |
| ASSTDIEAF | YLQPRTFLL | 0.9818 |
| ASSVDIEAF | YLQPRTFLL | 0.8897 |
| ASSVPDRANTGELF | YLQPRTFLL | 0.9762 |
| ASSWDIEAF | YLQPRTFLL | 0.9351 |
| ASTQDIEAF | YLQPRTFLL | 0.9671 |
| ASTRDIEAF | YLQPRTFLL | 0.9465 |
| ATGSANTGELF | YLQPRTFLL | 0.9984 |
| ATQDVNTGELF | YLQPRTFLL | 0.9992 |
| SCGNANTGELF | YLQPRTFLL | 0.9882 |
| ASNHANTGELF | YLQPRTFLL | 0.9779 |
| ASSASYYEQY | YLQPRTFLL | 0.2982 |
| ASSELGGGNTGELF | YLQPRTFLL | 0.9955 |
| ASSHTNTGELF | YLQPRTFLL | 0.9400 |
| ASSVDNTGELF | YLQPRTFLL | 0.9494 |
| ATSTENTGELF | YLQPRTFLL | 0.9939 |
| ASSVPGDNEQY | RLQSLQTYV | 0.2510 |
| ASSLGGAGGADTQY | RLQSLQTYV | 0.6902 |
| ASTWGRASTDTQY | RLQSLQTYV | 0.7797 |
| ASSPPTSGTANEQF | RLQSLQTYV | 0.1988 |
| ASSILAGAADTQY | RLQSLQTYV | 0.5216 |
| VRQDRNTGELF | RLQSLQTYV | 0.5676 |
| SALTPALAGGVPETQY | RLQSLQTYV | 0.4009 |
| SASPPGTSVNNEQF | RLQSLQTYV | 0.1825 |
| ASRGEQGYQETQY | RLQSLQTYV | 0.6517 |
| ASSLGTGHQPQH | RLQSLQTYV | 0.5732 |
| ATYIGNTGELF | RLQSLQTYV | 0.7193 |
| SVEGDSYEQY | RLQSLQTYV | 0.5574 |
| ASSQDWRAGAYNEQF | RLQSLQTYV | 0.6678 |
| AWSAGQGMNTEAF | RLQSLQTYV | 0.7053 |
| AWSGGQIMNTEAF | RLQSLQTYV | 0.4801 |
| AWSVGAGMKYGYT | RLQSLQTYV | 0.6700 |
| ASSQDEYNTGELF | RLQSLQTYV | 0.6766 |
| ASSQDLQGTNEKLF | RLQSLQTYV | 0.8877 |
| ASSQGDGANVLT | RLQSLQTYV | 0.5342 |
| ASSQGQGNIQY | RLQSLQTYV | 0.3897 |
| ASSLGTGYQPQH | RLQSLQTYV | 0.5607 |
| ASSPGTGWAEAF | RLQSLQTYV | 0.7394 |
| ASSYEKGIMNTEAF | RLQSLQTYV | 0.6822 |
| ASTDRDTETQY | RLQSLQTYV | 0.4916 |
| ASTEGTGWTEAF | RLQSLQTYV | 0.7713 |
| ATSGGTGHQPQH | RLQSLQTYV | 0.6414 |
| ATTERDQETQY | RLQSLQTYV | 0.6405 |
| ASSELLAGGLETQY | RLQSLQTYV | 0.5632 |
| AYLEANSYEQY | YLQPRTFLL | 0.9323 |
| ASLRDMNTGELF | YLQPRTFLL | 0.9677 |
| ASREGNTGELF | YLQPRTFLL | 0.9943 |
| ASSQTESTDTQY | YLQPRTFLL | 0.0342 |
| ASQLMNTGELF | YLQPRTFLL | 0.9863 |
| ASSPVGGGNTGELF | YLQPRTFLL | 0.9836 |
| AAQDSNTGELF | YLQPRTFLL | 0.9998 |
| AGGVNPNTGELF | YLQPRTFLL | 0.9747 |
| AIADANTGELF | YLQPRTFLL | 1.0000 |
| ASGDDNTGELF | YLQPRTFLL | 0.9999 |
| ASGDENTGELF | YLQPRTFLL | 0.9996 |
| ASGEENTGELF | YLQPRTFLL | 0.9987 |
| ASGRQNTGELF | YLQPRTFLL | 0.9826 |
| ASGTQNTGELF | YLQPRTFLL | 0.9979 |
| ASIDQNTGELF | YLQPRTFLL | 0.9989 |
| ASMEENTGELF | YLQPRTFLL | 0.9861 |
| ASSDANTGELF | YLQPRTFLL | 0.9997 |
| ASSDDNTGELF | YLQPRTFLL | 0.9994 |
| ASSDGNTGELF | YLQPRTFLL | 0.9902 |
| ASSDGTSNEQY | YLQPRTFLL | 0.2341 |
| ASSDTNTGELF | YLQPRTFLL | 0.9985 |
| ASSEGTSGEQY | YLQPRTFLL | 0.2422 |
| ASSGLNTGELF | YLQPRTFLL | 0.9025 |
| ASSLVPEEQY | YLQPRTFLL | 0.2855 |
| ASYDLNTGELF | YLQPRTFLL | 0.9987 |
| ATQVDQNTGELF | YLQPRTFLL | 0.7023 |
| AVQDSNTGELF | YLQPRTFLL | 0.9998 |
| AIGDRSSGEQY | YLQPRTFLL | 0.8166 |
| ASIANRNTGELF | YLQPRTFLL | 0.8231 |
| ASIPDRNTGELF | YLQPRTFLL | 0.8768 |
| ASSPDMNTGELF | YLQPRTFLL | 0.9961 |
| ASSPLVSRNTGELF | YLQPRTFLL | 0.9902 |
| ASSPTAGGDTGELF | YLQPRTFLL | 0.9991 |
| ASSPTAGGNTGELF | YLQPRTFLL | 0.9985 |
| AIGDRNTGELF | YLQPRTFLL | 0.9998 |
| AAGVENTGELF | YLQPRTFLL | 0.9992 |
| ASSTLNSLEAF | YLQPRTFLL | 0.9452 |
| ASSLTGGGNTGELF | YLQPRTFLL | 0.7961 |
| APQDLDTGELF | YLQPRTFLL | 0.9988 |
| ASSARNSIEAF | YLQPRTFLL | 0.8780 |
| ASSEWIQETQY | YLQPRTFLL | 0.5756 |
| ASQDRNTGELF | YLQPRTFLL | 0.9974 |
| ASSSQNTAEAF | YLQPRTFLL | 0.8052 |
| ASSYRNSLEQF | YLQPRTFLL | 0.4795 |
| ASTELNGGTQY | YLQPRTFLL | 0.0967 |
| ATEDLNTGELF | YLQPRTFLL | 0.9994 |
| ASEDRNTGELF | YLQPRTFLL | 0.9987 |
| ATEDRNTGELF | YLQPRTFLL | 0.9991 |
| AEGELNTGELF | YLQPRTFLL | 0.9994 |
| AGQDLNTGELF | YLQPRTFLL | 0.9997 |
| ALGDLNTGELF | YLQPRTFLL | 0.9997 |
| APGDLNTGELF | YLQPRTFLL | 0.9997 |
| ASRPTGLAENTGELF | YLQPRTFLL | 0.9835 |
| ASSSVNNNEQF | YLQPRTFLL | 0.1783 |
| AASEMNTGELF | YLQPRTFLL | 0.9987 |
| AGADSNTGELF | YLQPRTFLL | 0.9999 |
| AGDYLNTGELF | YLQPRTFLL | 0.9977 |
| AGGDSNTGELF | YLQPRTFLL | 0.9998 |
| AIGDENTGELF | YLQPRTFLL | 0.9999 |
| ALGEPNTGELF | YLQPRTFLL | 0.9999 |
| ALGSSNTGELF | YLQPRTFLL | 0.9984 |
| ARGDQNTGELF | YLQPRTFLL | 0.9996 |
| ARGESNTGELF | YLQPRTFLL | 0.9993 |
| ASLSQNTGELF | YLQPRTFLL | 0.9884 |
| ASNDLNTGELF | YLQPRTFLL | 0.9994 |
| ASNNLNTGELF | YLQPRTFLL | 0.9975 |
| ASNNQNTGELF | YLQPRTFLL | 0.9886 |
| ASSDSYGYT | YLQPRTFLL | 0.6462 |
| ASSELNTGELF | YLQPRTFLL | 0.9953 |
| ASSFQNTGELF | YLQPRTFLL | 0.9566 |
| ASSNSFGYT | YLQPRTFLL | 0.6851 |
| ASSNSYGYT | YLQPRTFLL | 0.4496 |
| ASSVQNTGELF | YLQPRTFLL | 0.9253 |
| ASSYQNTGELF | YLQPRTFLL | 0.9816 |
| AGGDPNTGELF | YLQPRTFLL | 0.9999 |
| AKGTANTGELF | YLQPRTFLL | 0.9998 |
| ARGLADTGELF | YLQPRTFLL | 0.9939 |
| ASGPDLGYT | YLQPRTFLL | 0.9903 |
| ASNPPPGDSENTGELF | YLQPRTFLL | 0.8063 |
| ASSADIEQF | YLQPRTFLL | 0.8873 |
| ASSADIQQY | YLQPRTFLL | 0.7397 |
| ASSADVEAF | YLQPRTFLL | 0.9786 |
| ASSDDIEAF | YLQPRTFLL | 0.9997 |
| AGQVTNTGELF | YLQPRTFLL | 0.9961 |
| ASSSANSGELF | YLQPRTFLL | 0.7470 |
| ASTWGRASTDTQY | RLQSLQIYV | 0.7198 |

### Supplement Figure S1. A) Wenn Diagram of data sources and their overlap. B) Wenn Diagram of Training data, seen data and unseen data.


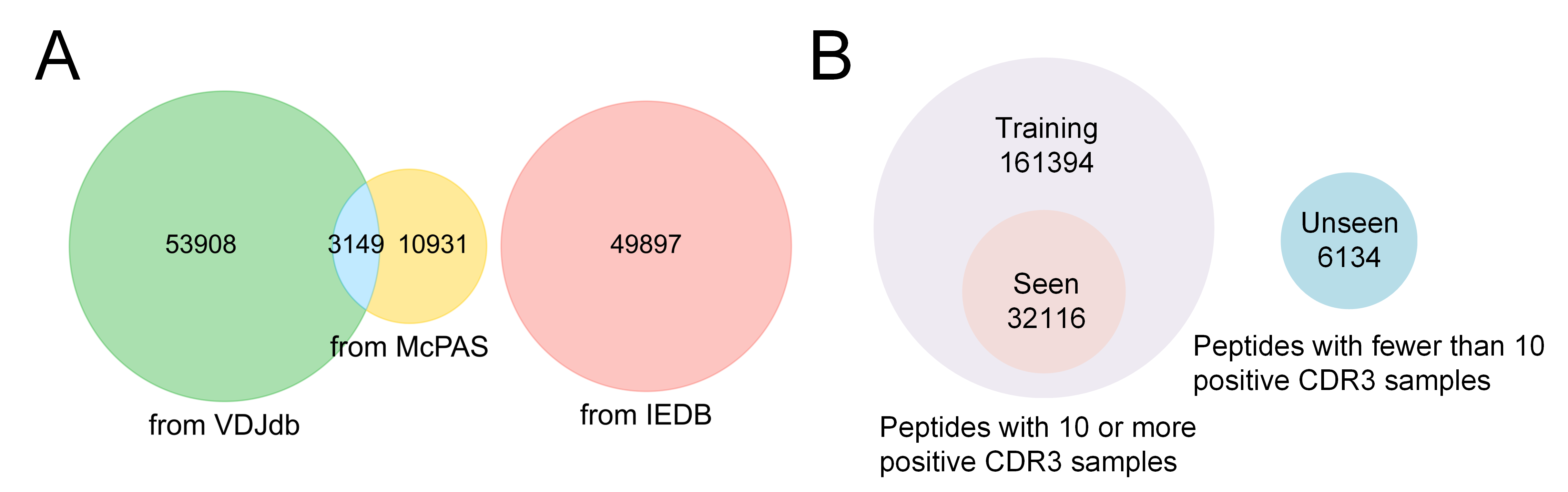


### Supplement Figure S2. Ablation experiments on the key modules of TPepRet.


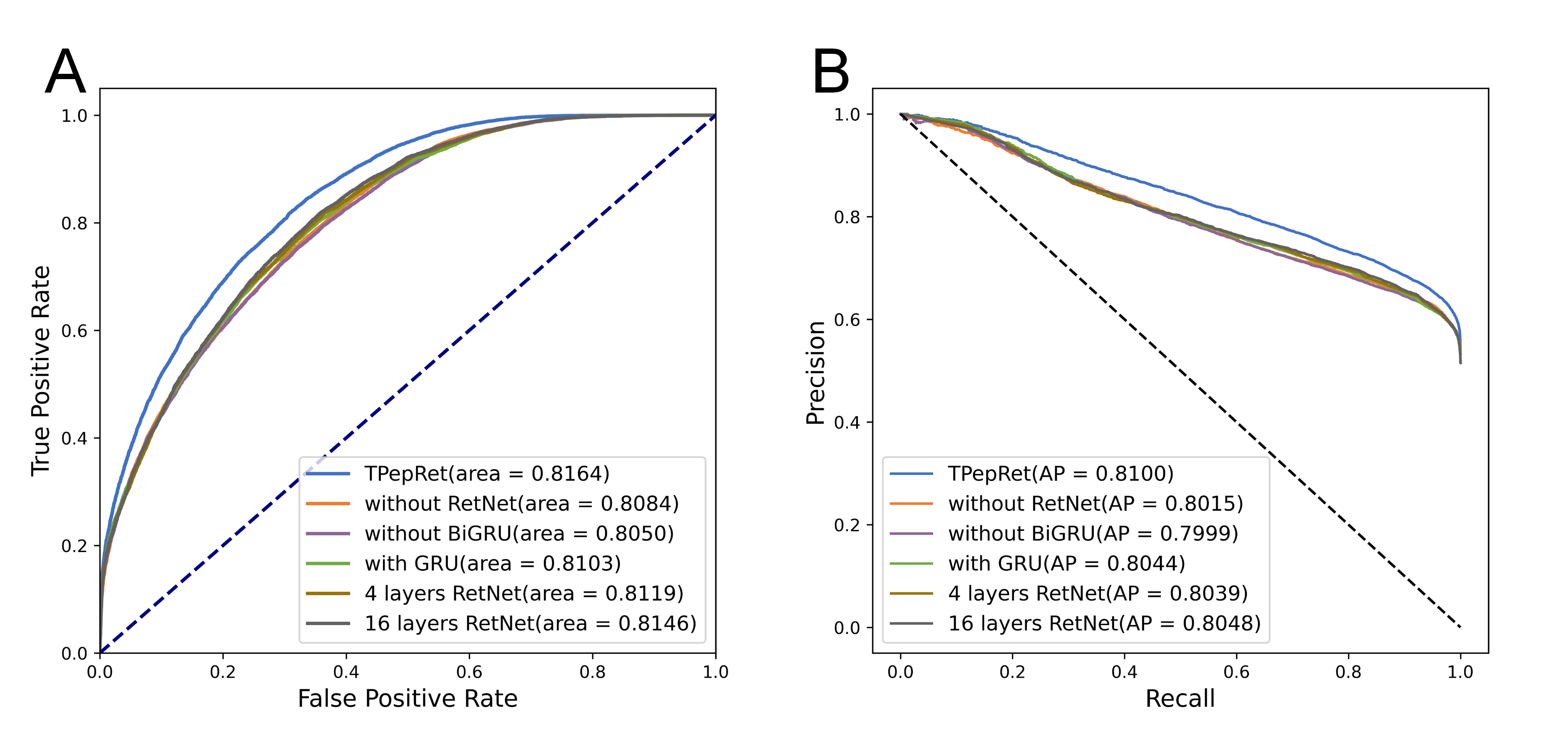


### Supplement Figure S3. Ggseqlogo of the real binding CDR3 sequences.


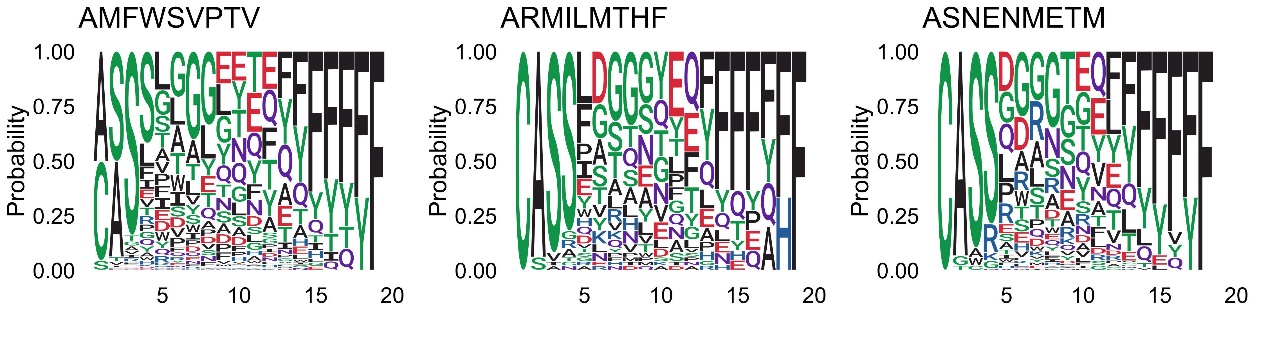

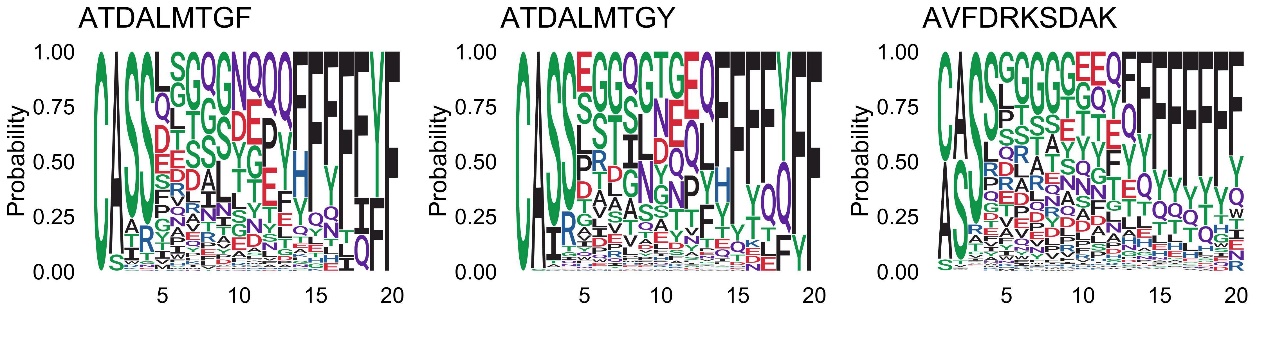

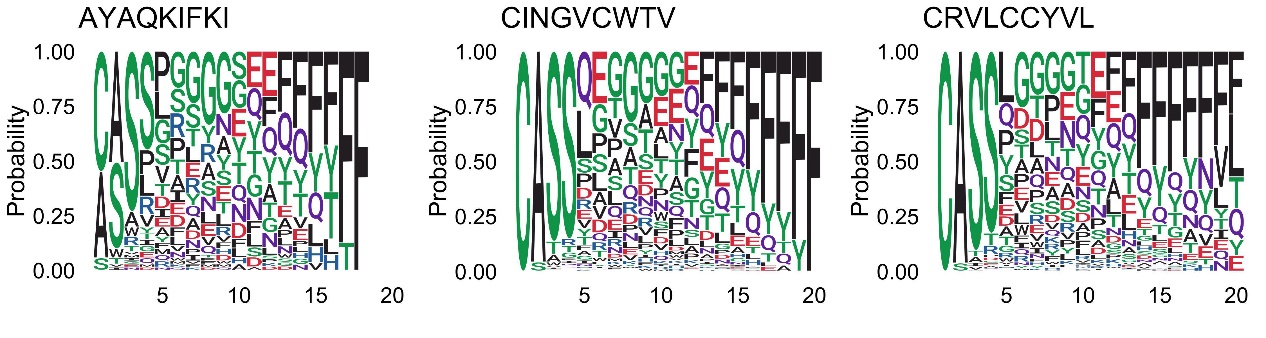

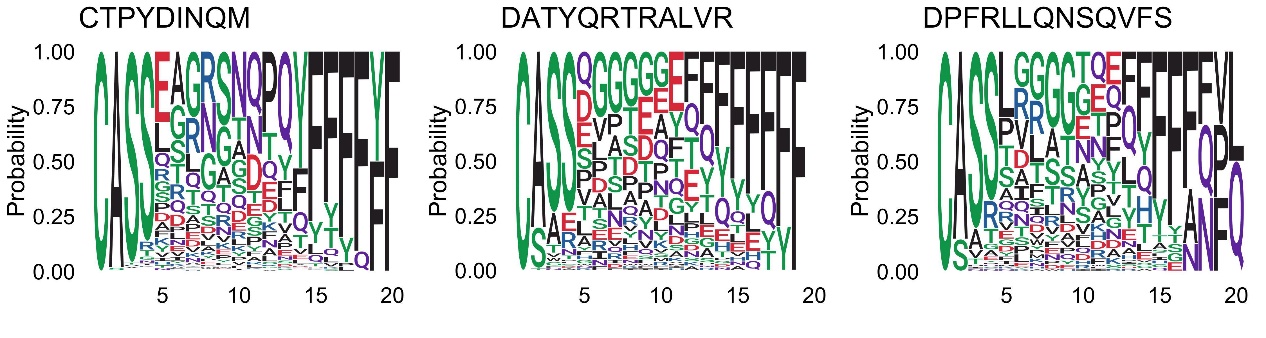

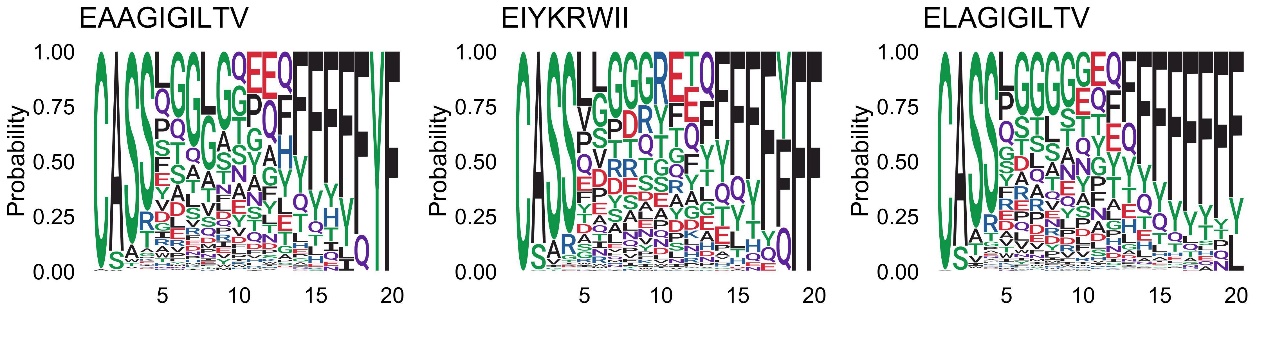

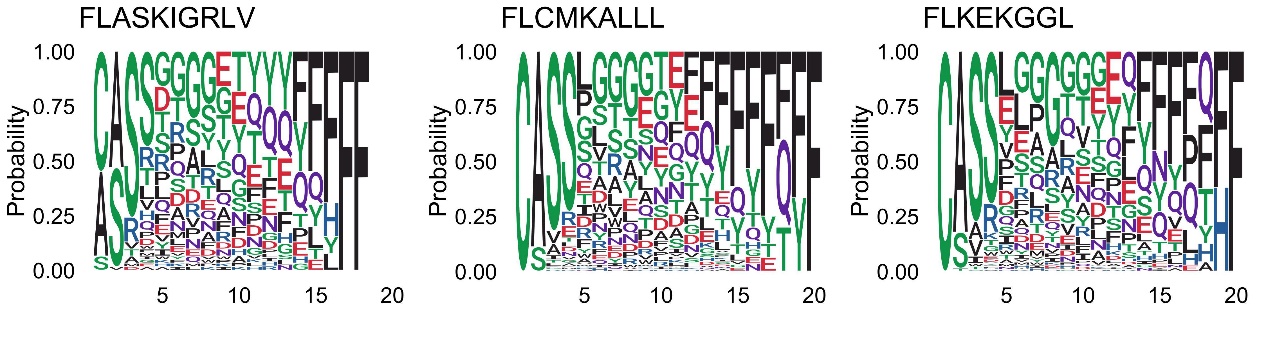

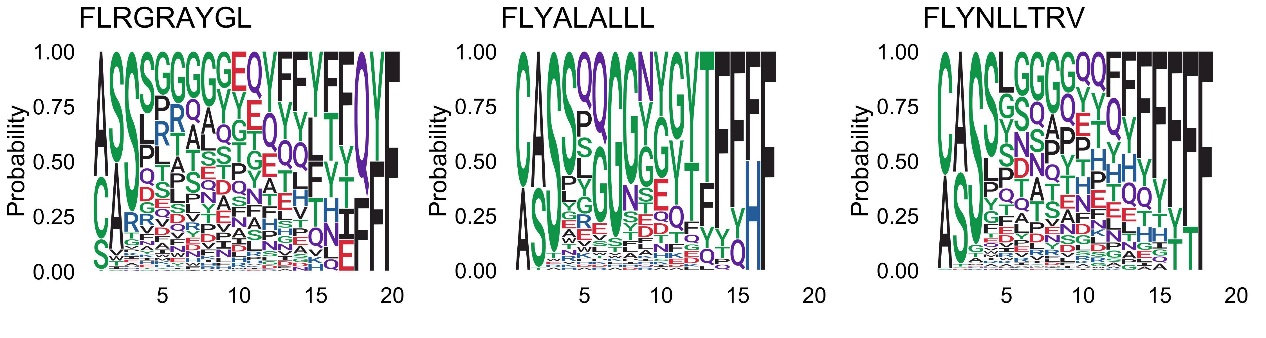

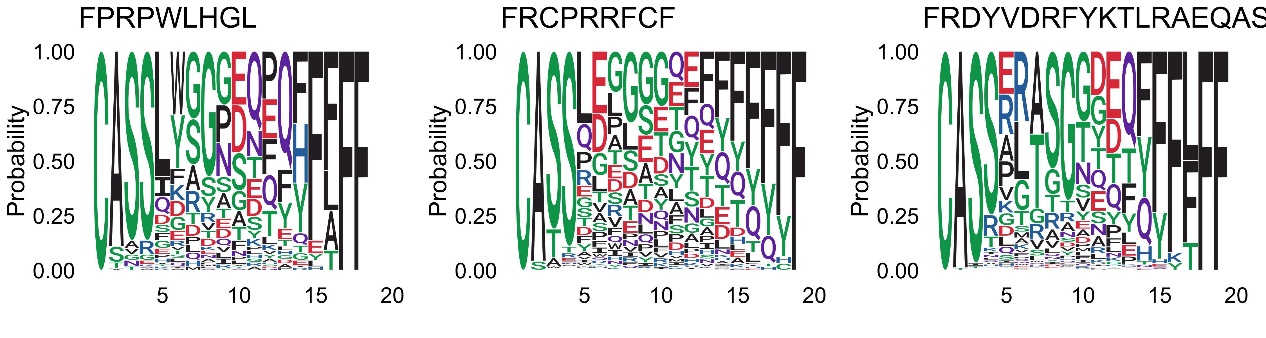

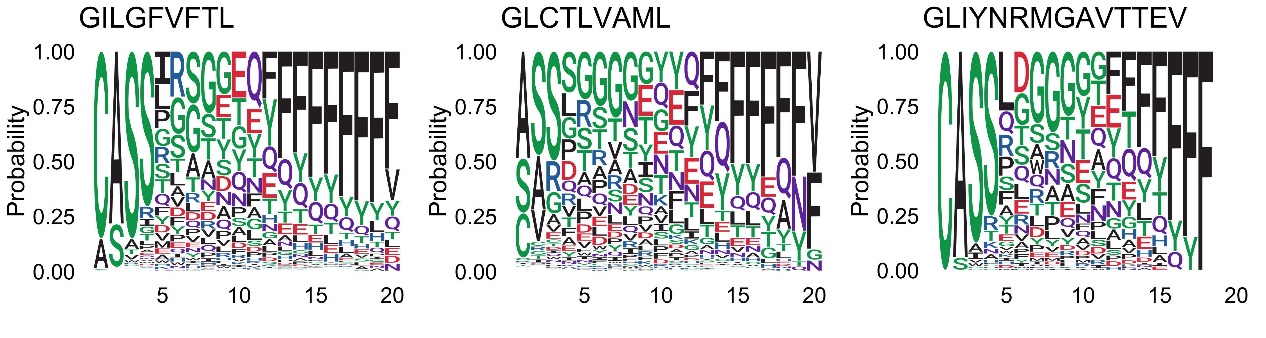

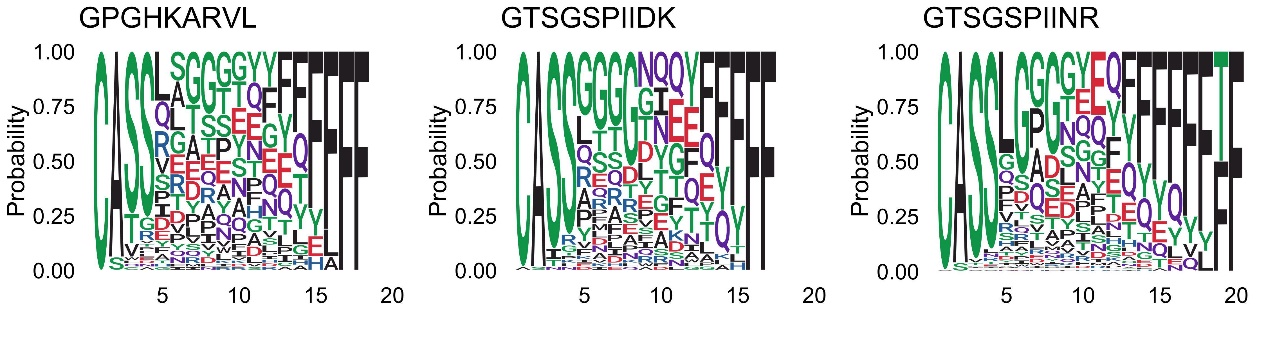

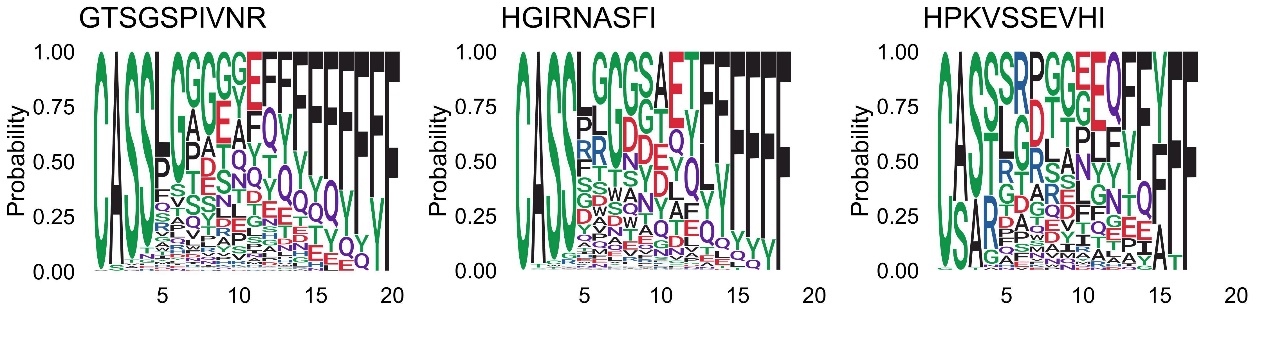

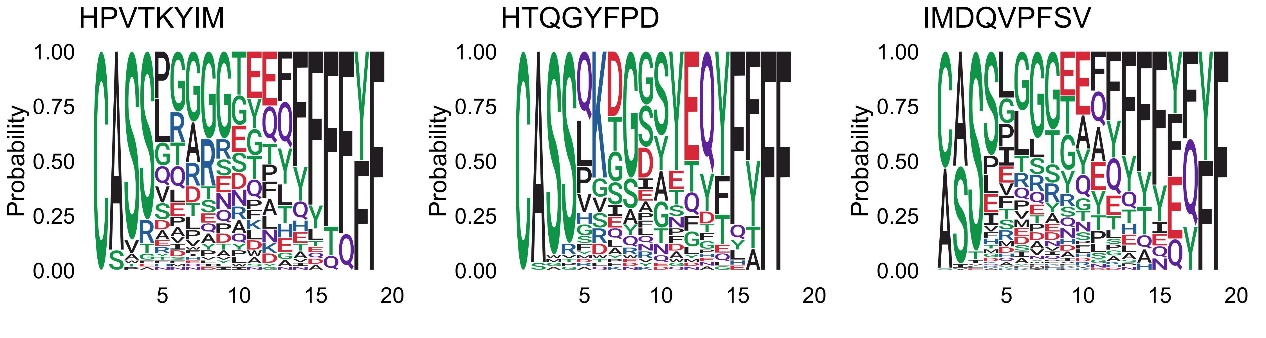

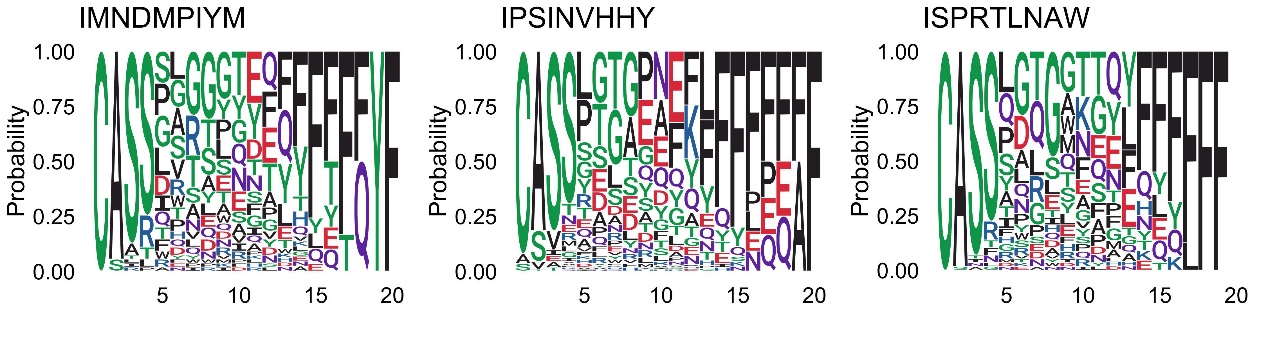

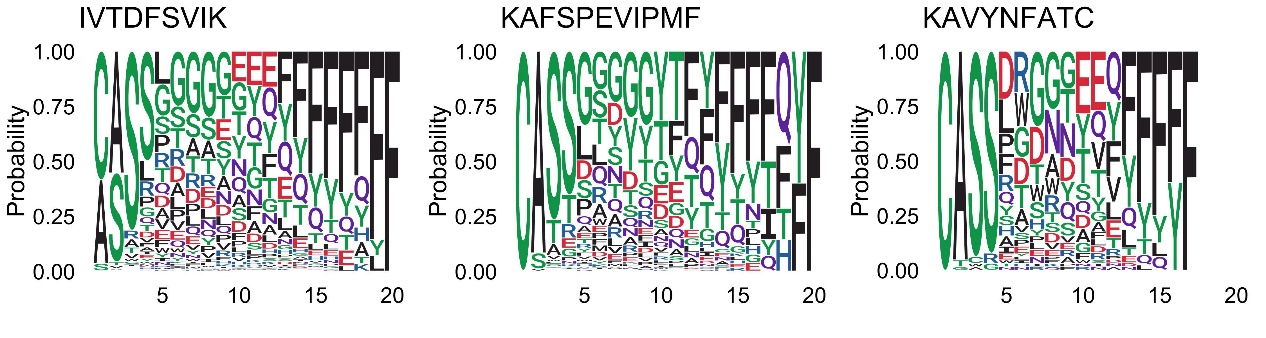

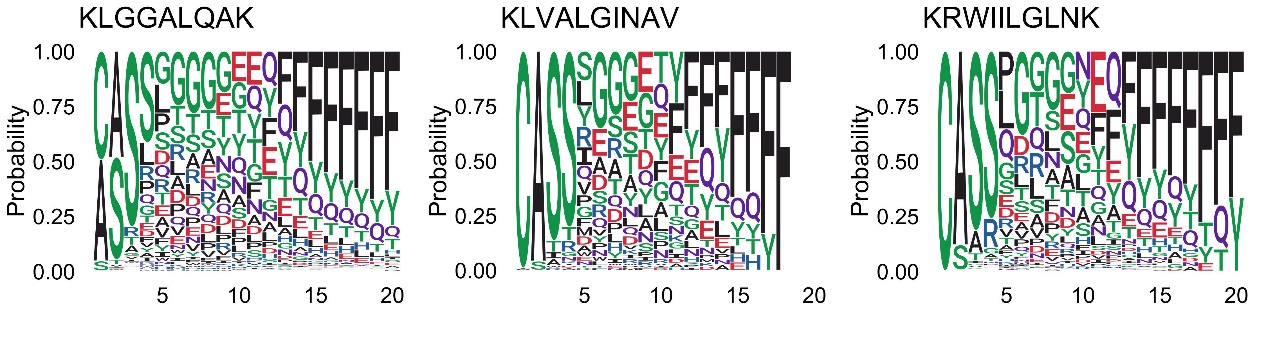

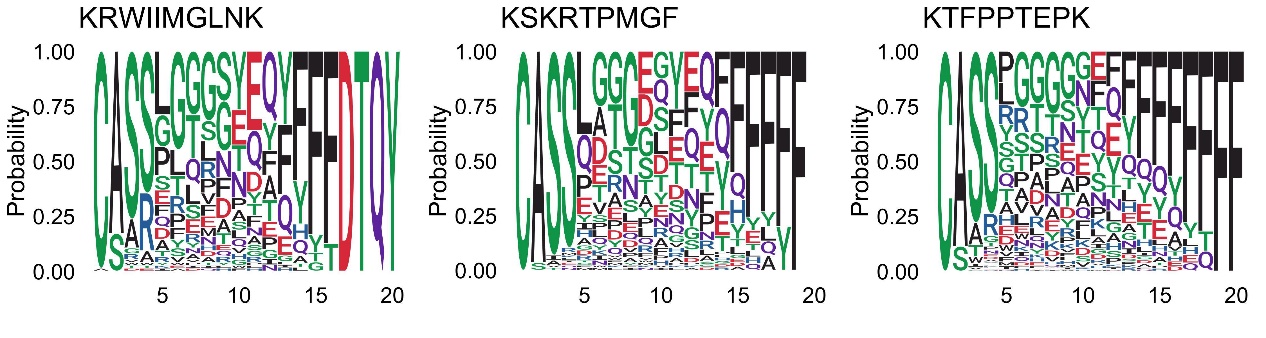

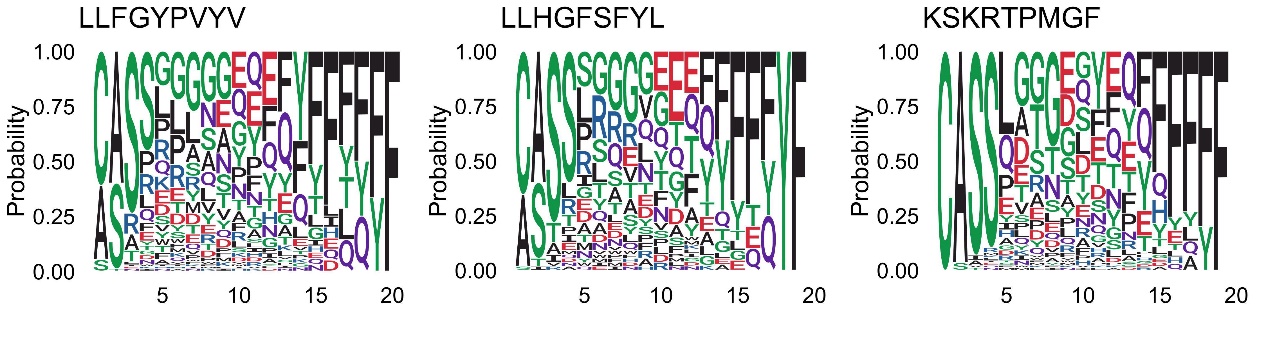

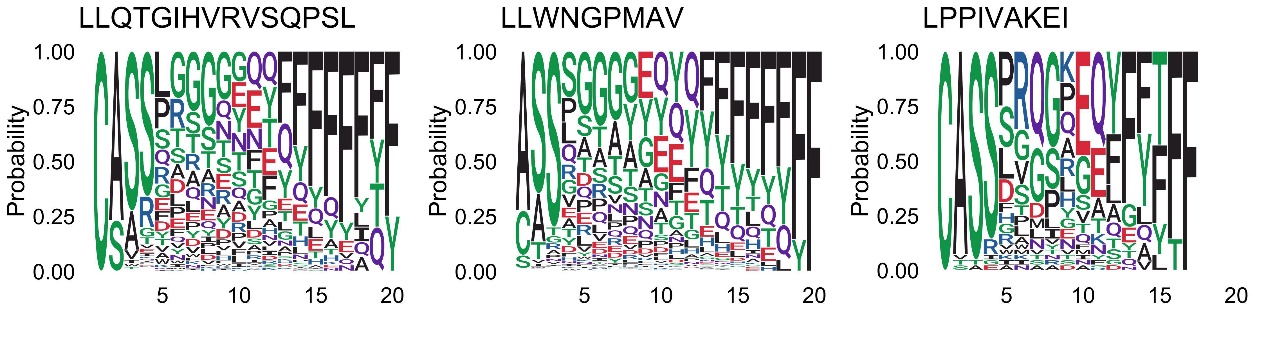

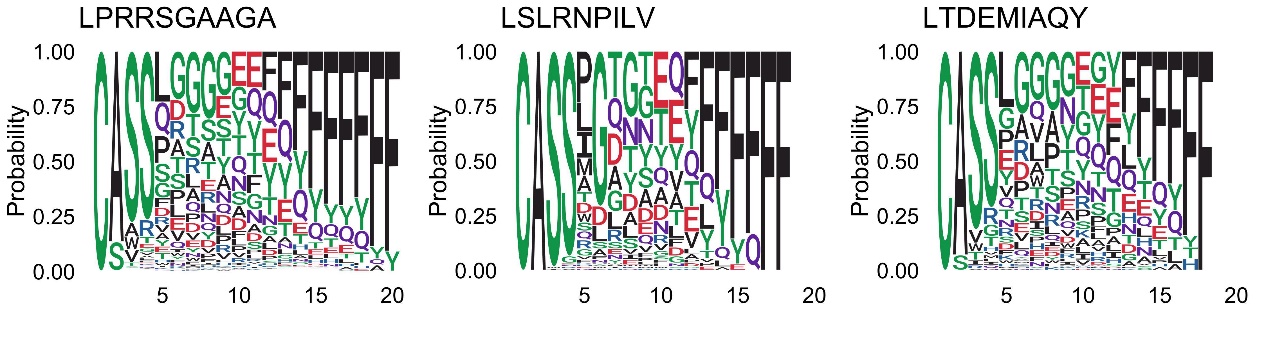

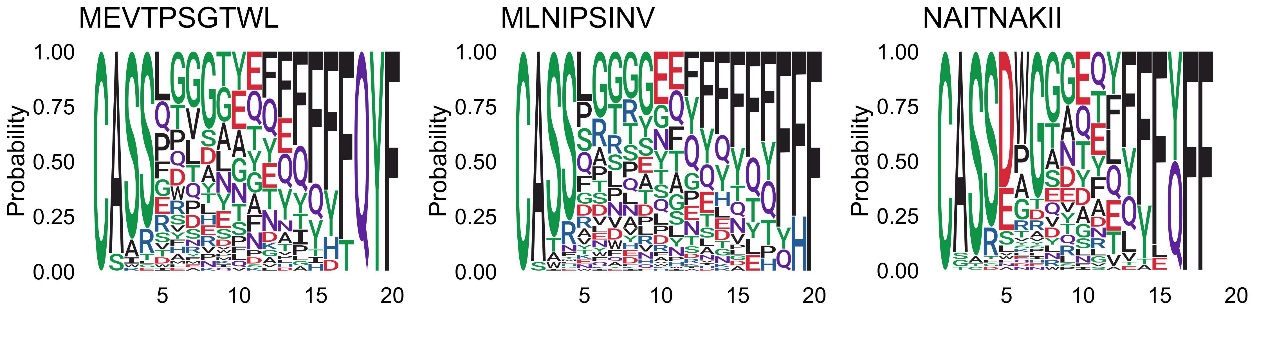

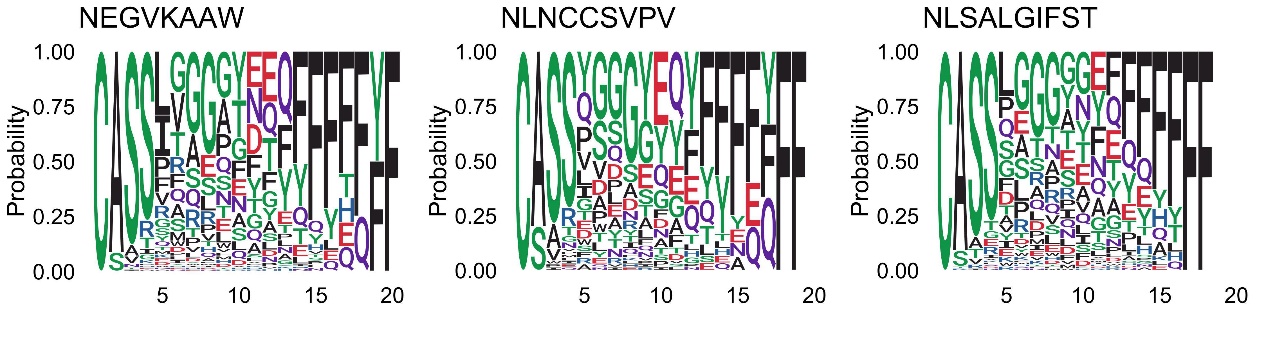

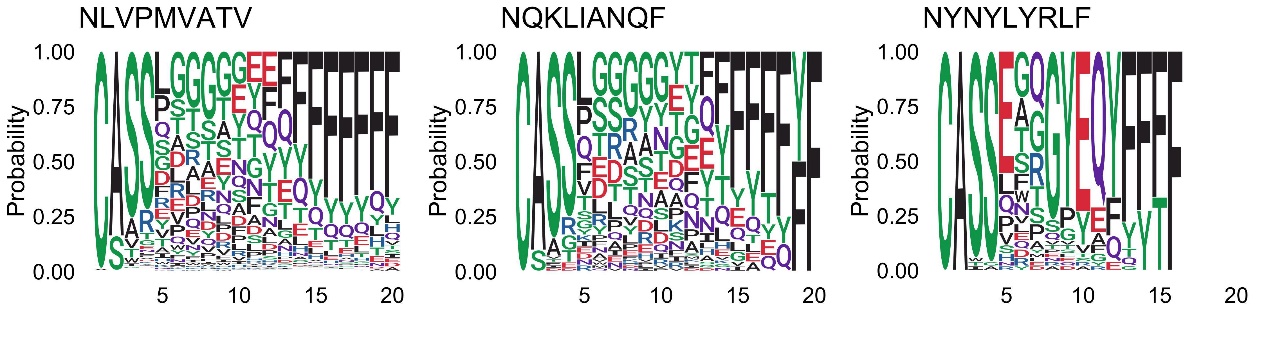

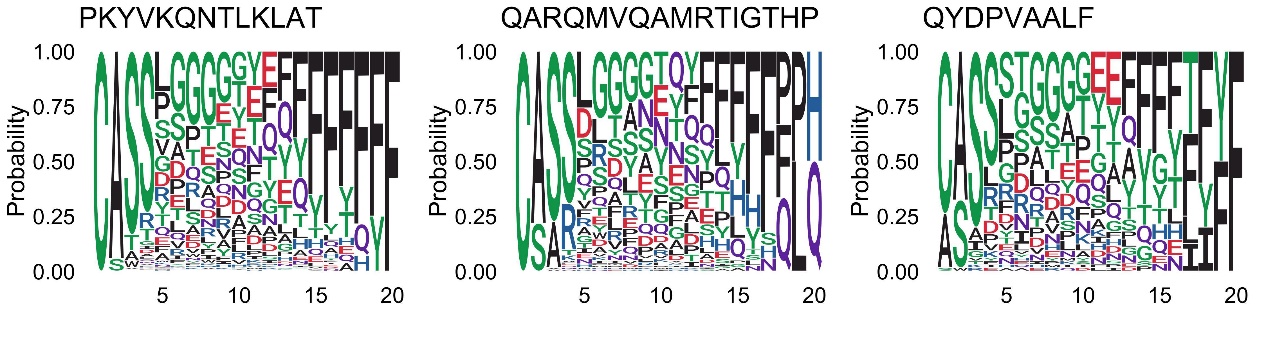

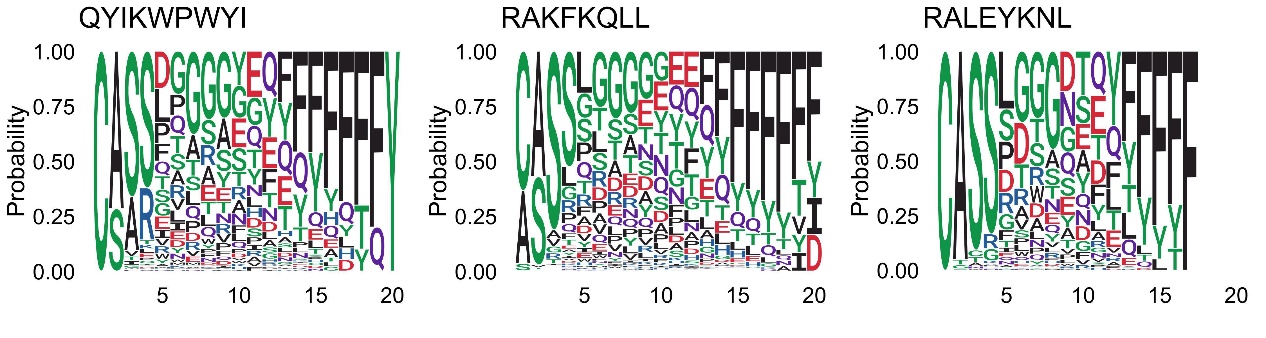

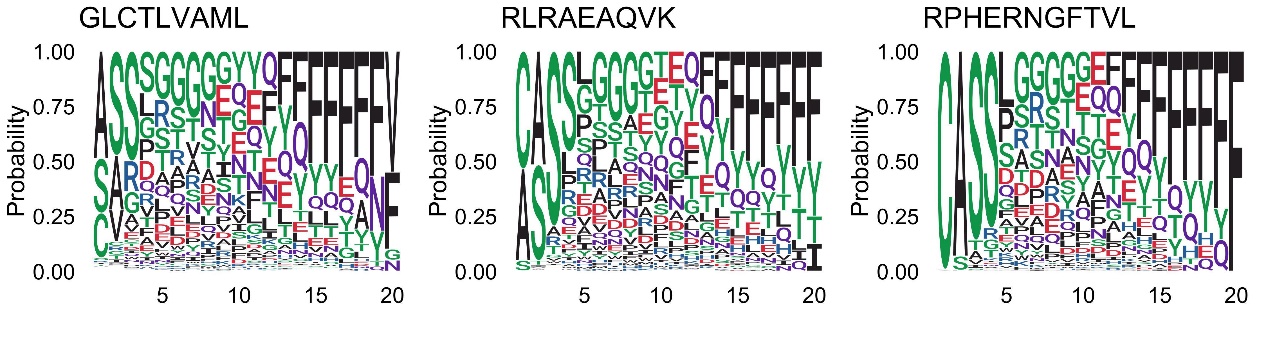

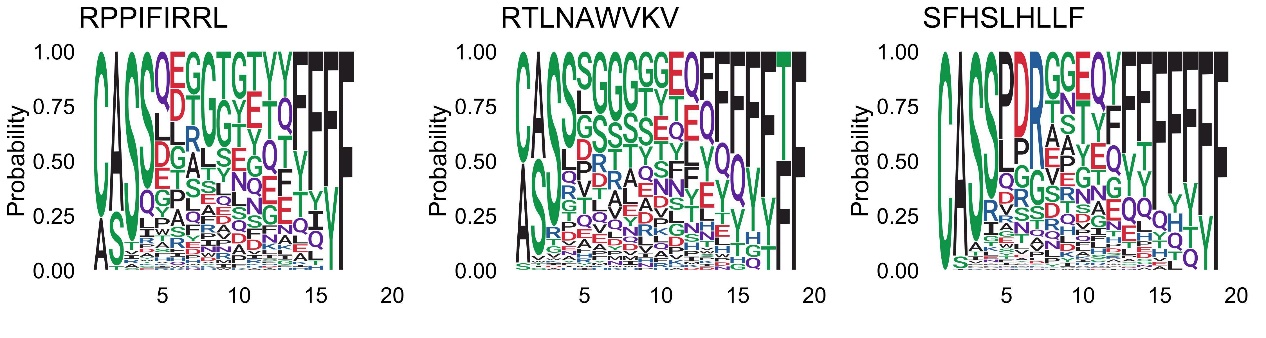

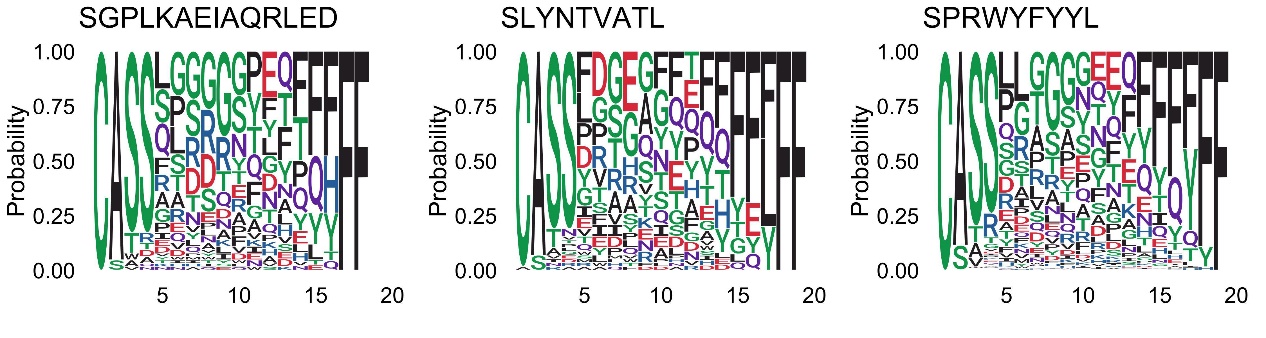

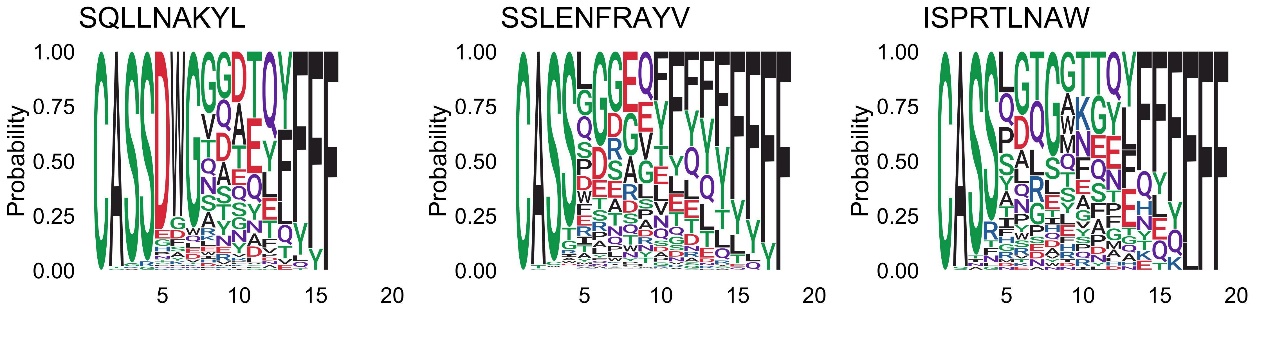

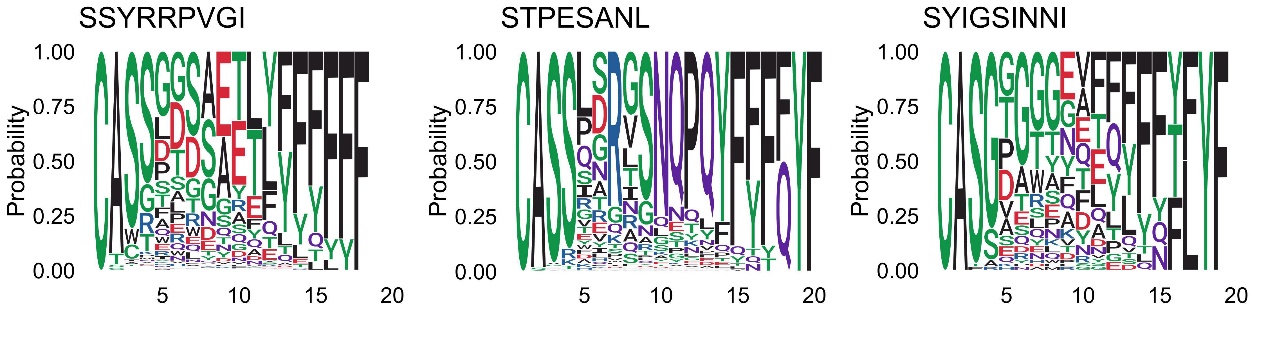

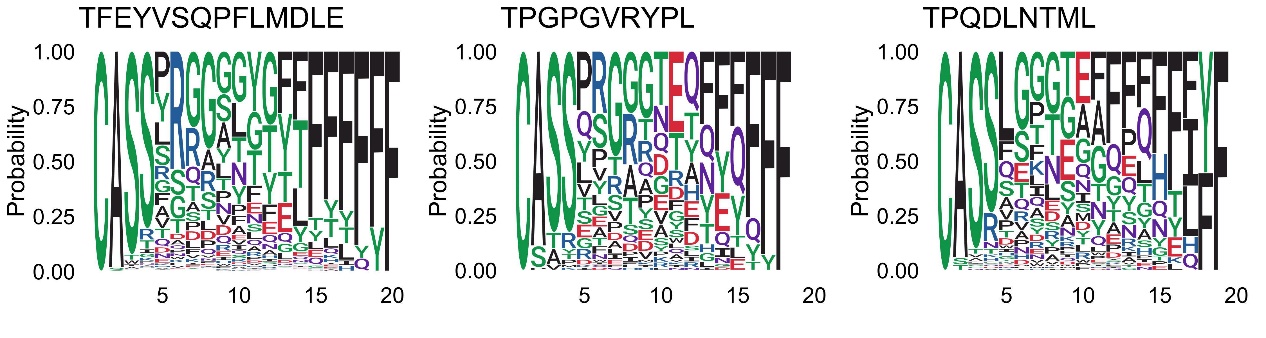

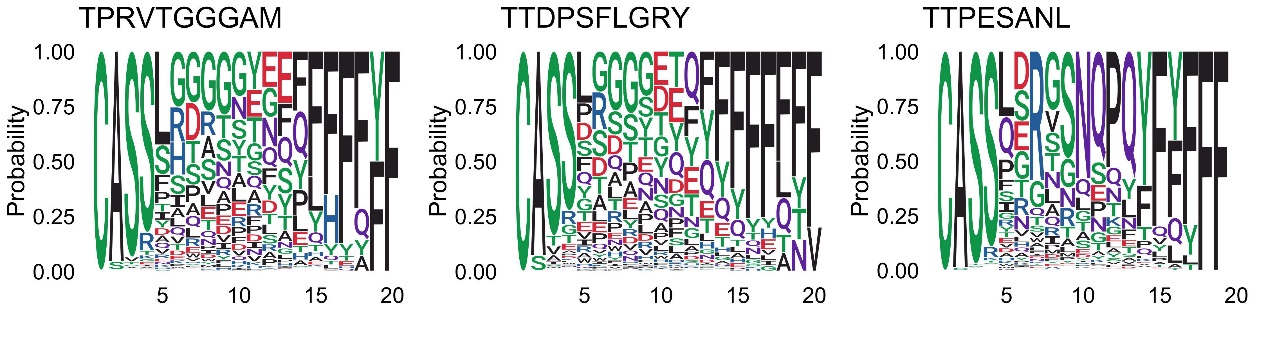

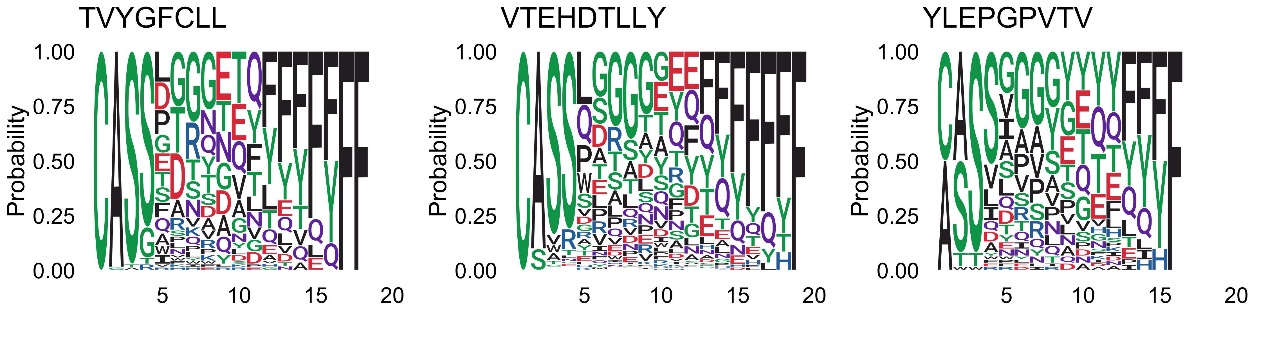

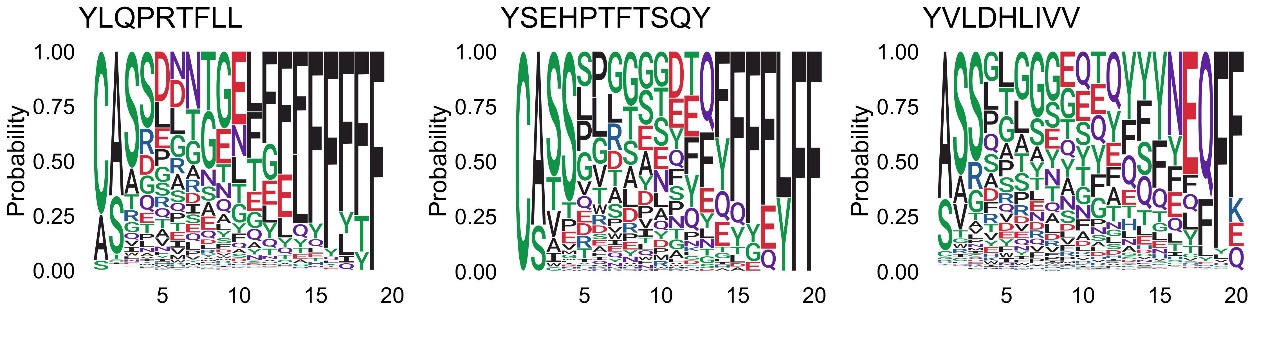


### Supplement Figure S4. Heat Map of top 5 peptides’ conjugates.


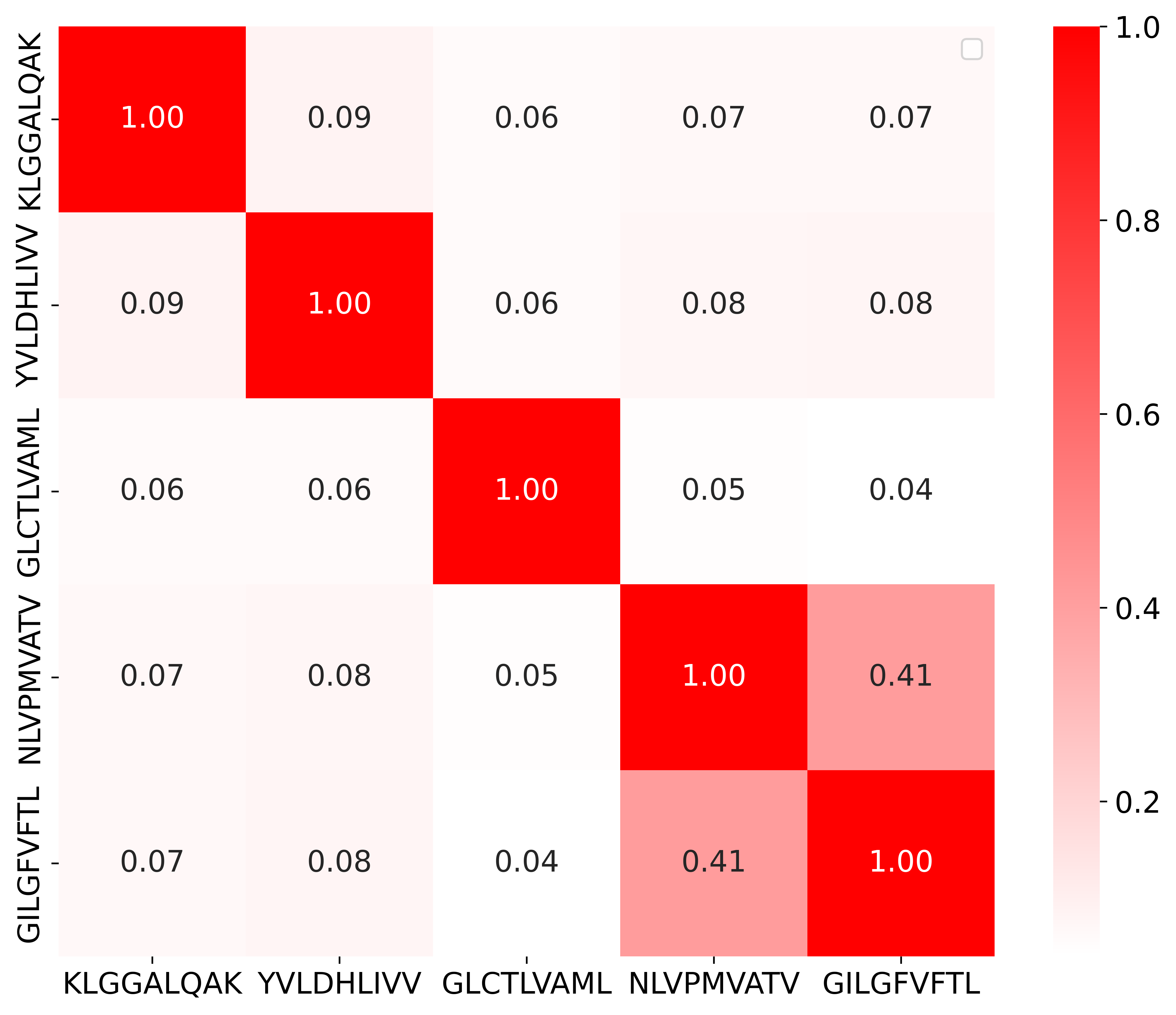


### Supplement Figure S5. The predicted ggseqlogo of the top N CDR3 sequences.

CINGVCWTV


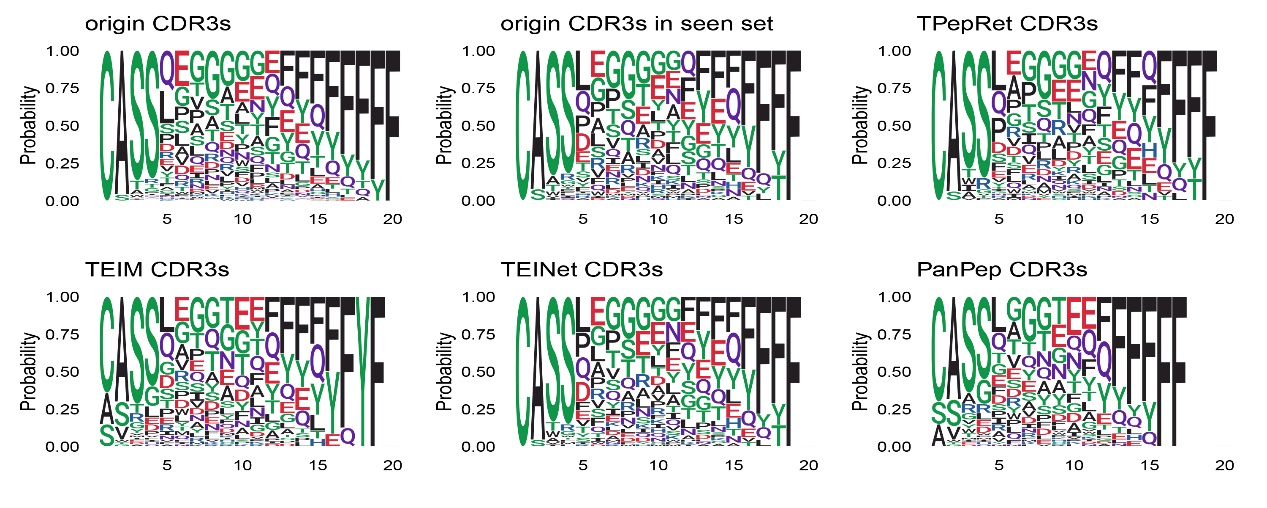


CRVLCCYVL


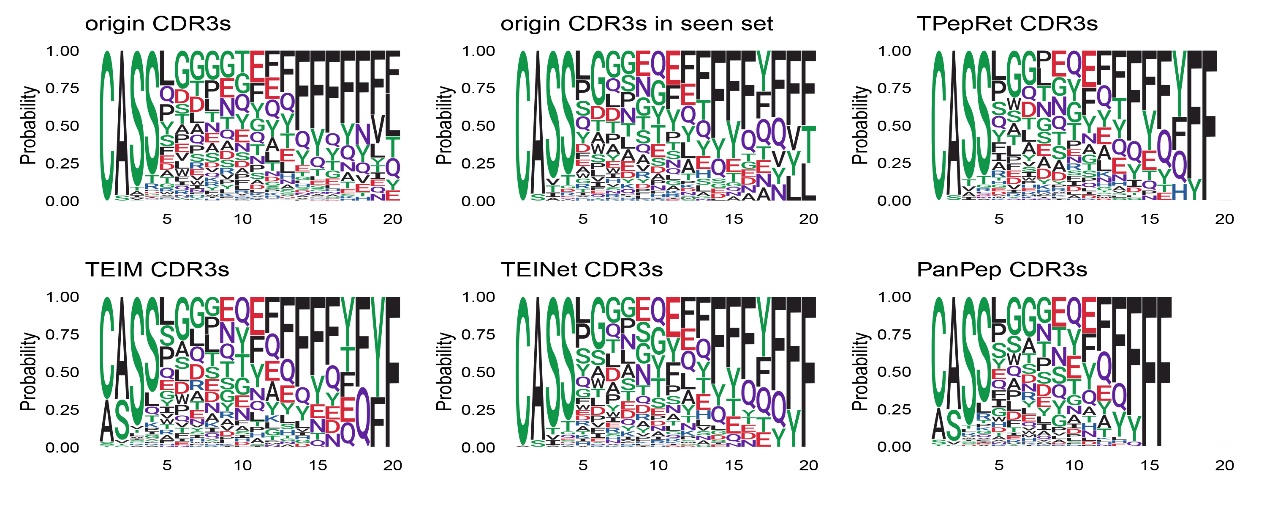


EAAGIGILTV


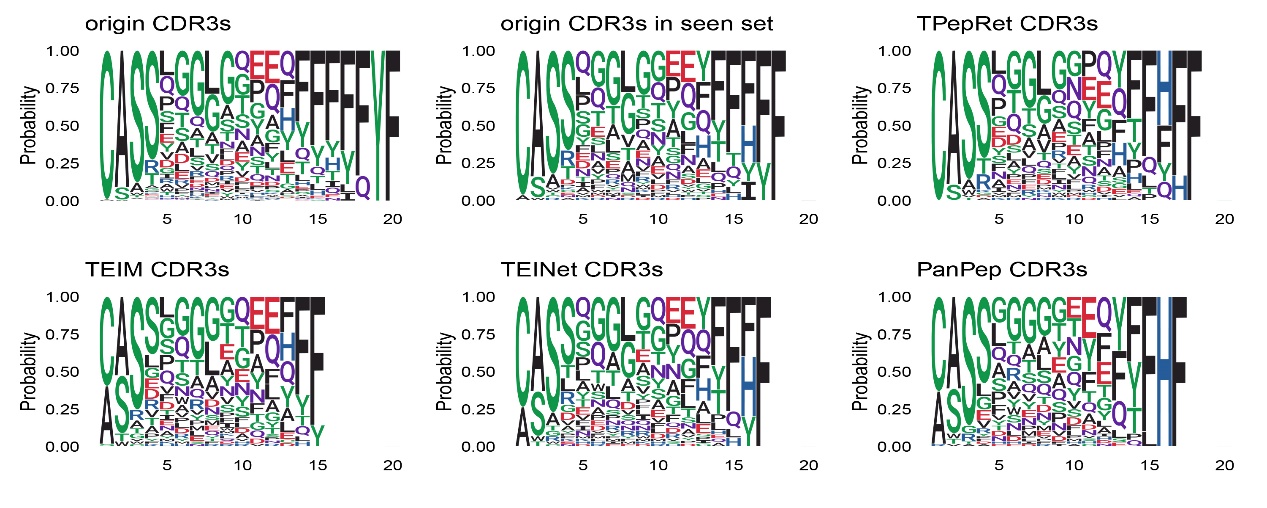


ELAGIGILTV


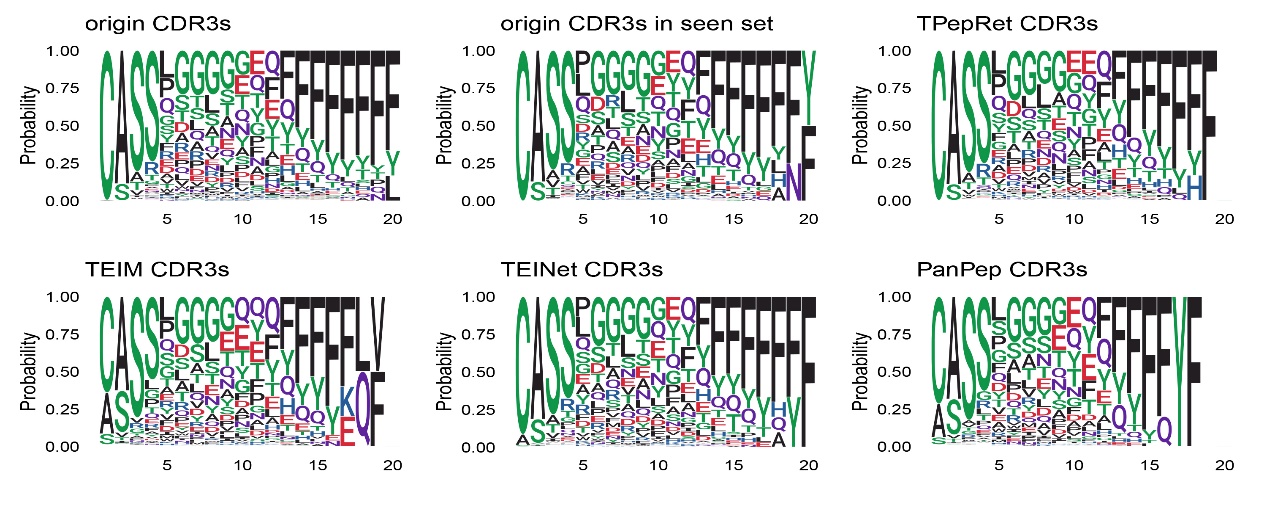


FRCPRRFCF


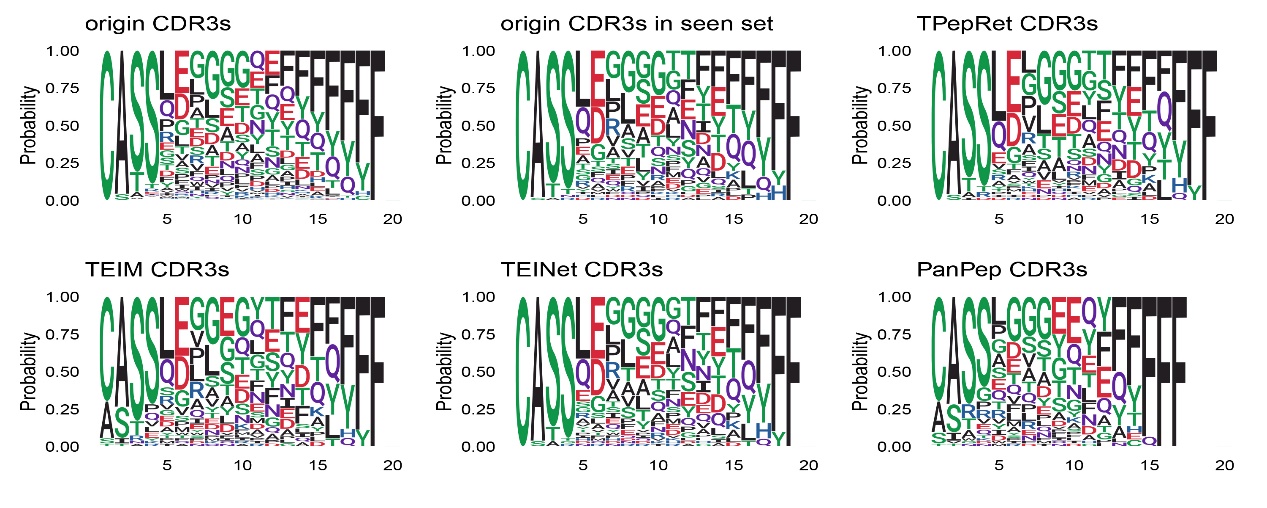


GILGFVFTL


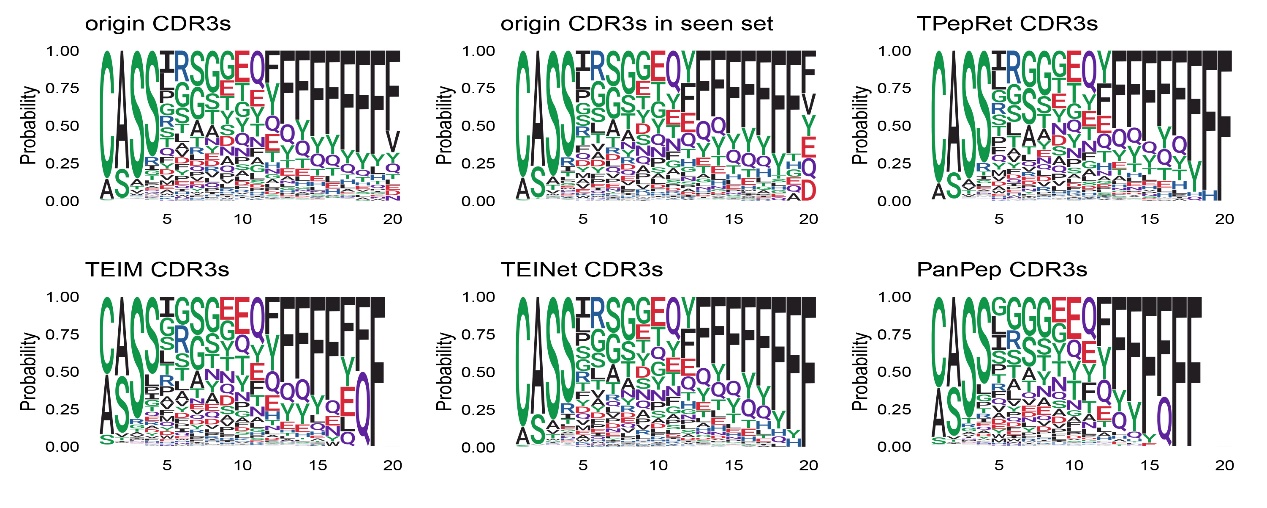


GLCTLVAML


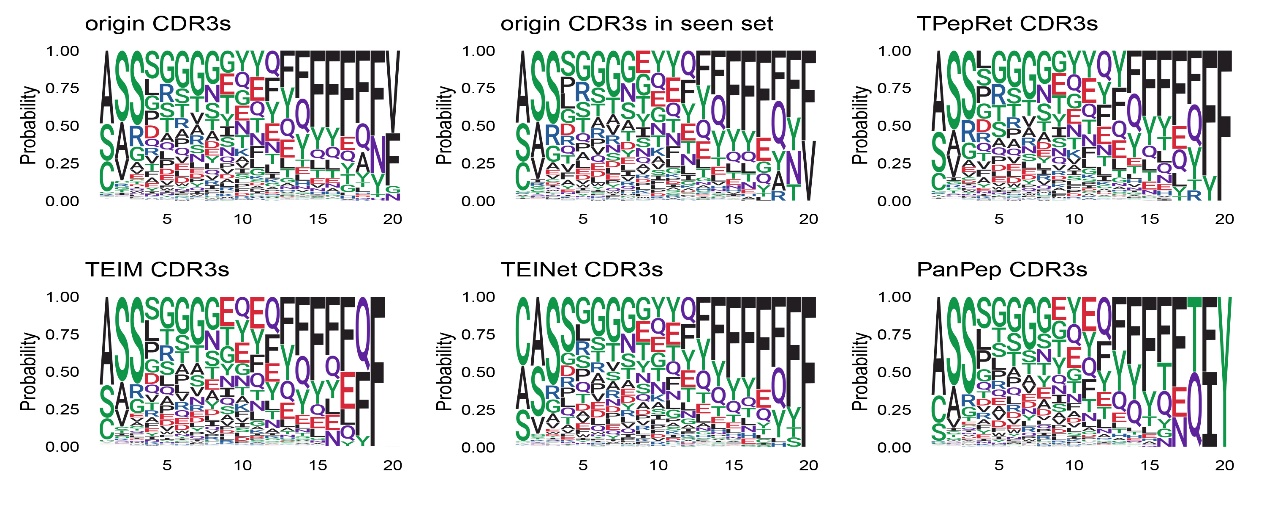


IVTDFSVIK


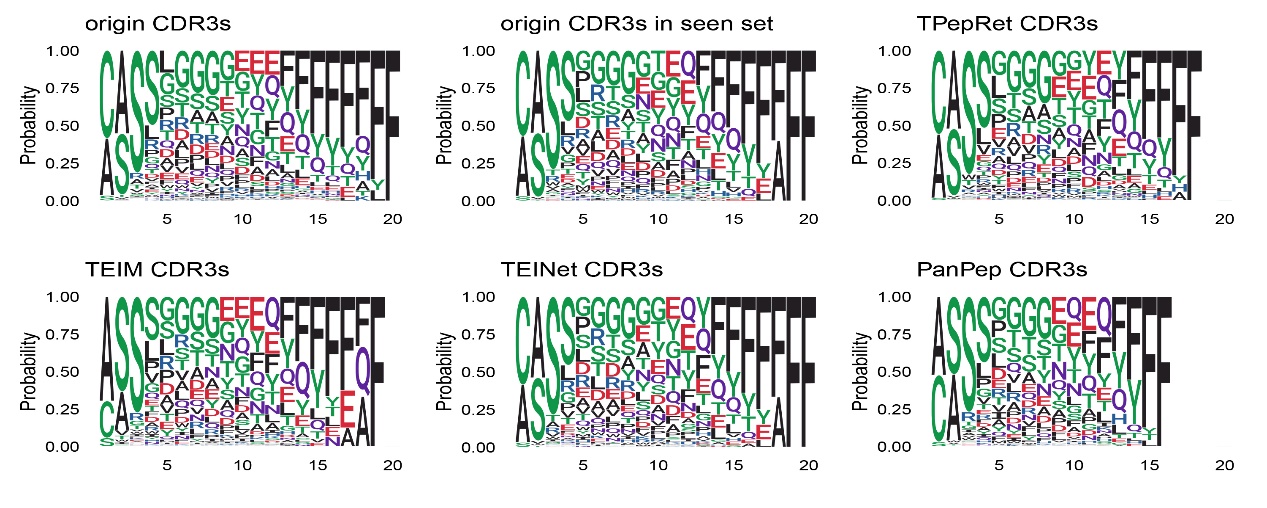


KAFSPEVIPMF


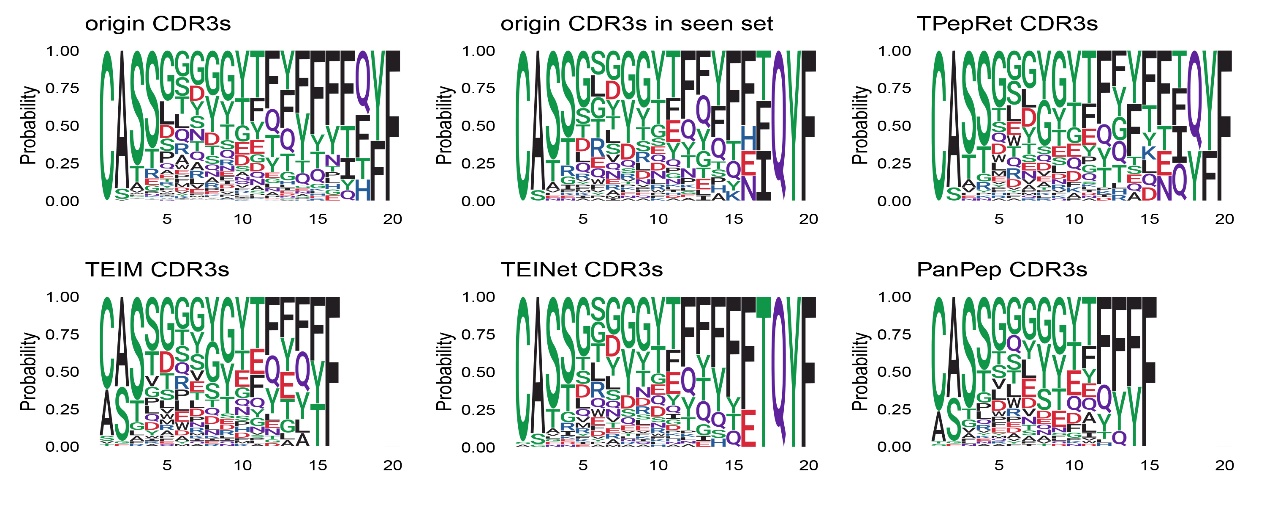


KRWIILGLNK


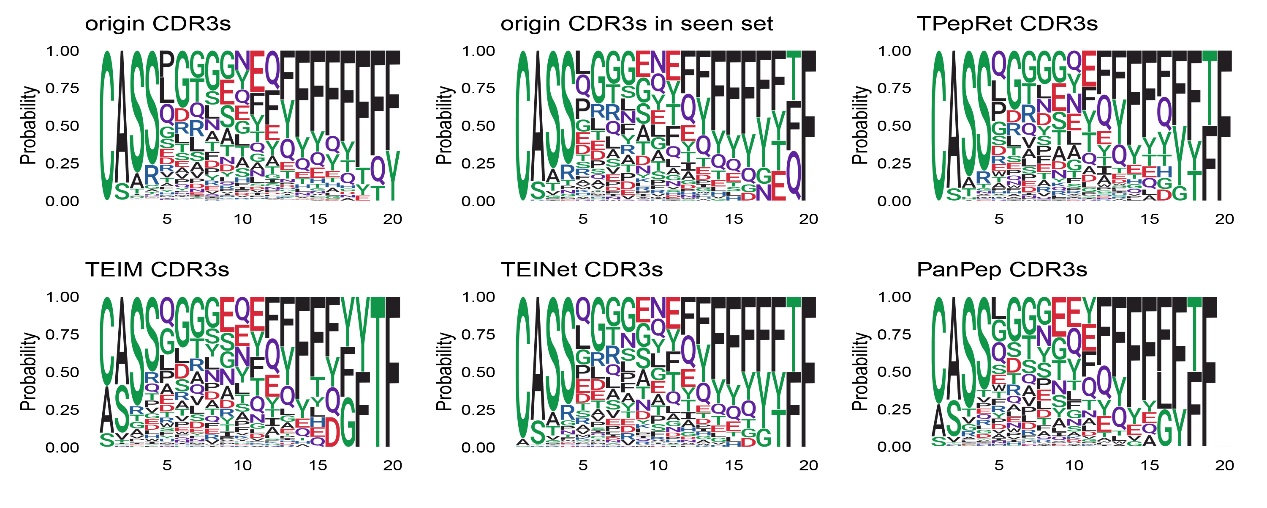


LLWNGPMAV


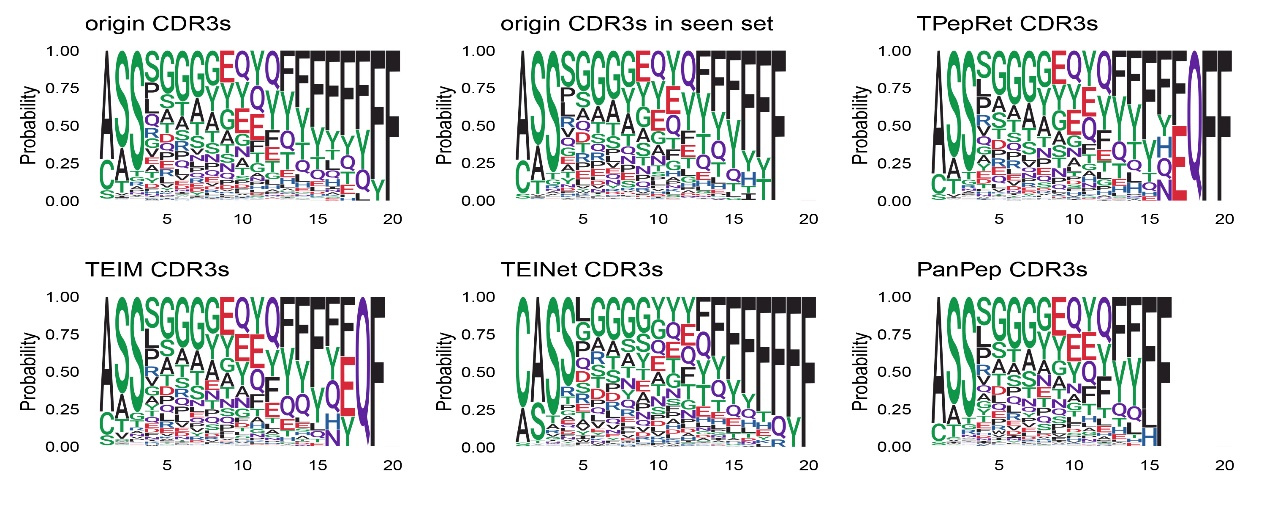


LPRRSGAAGA


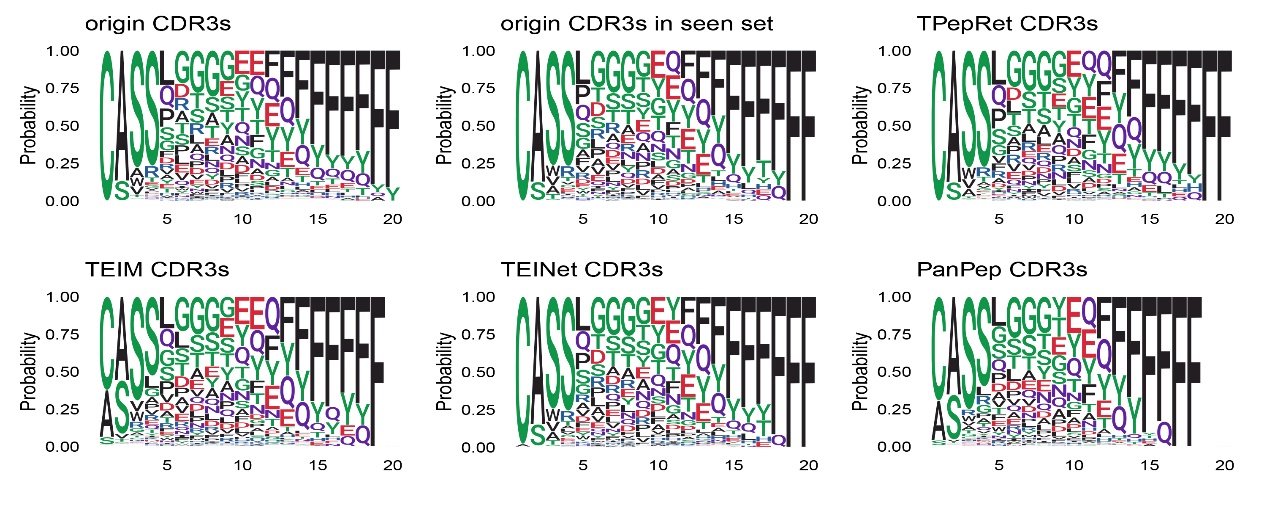


NLVPMVATV


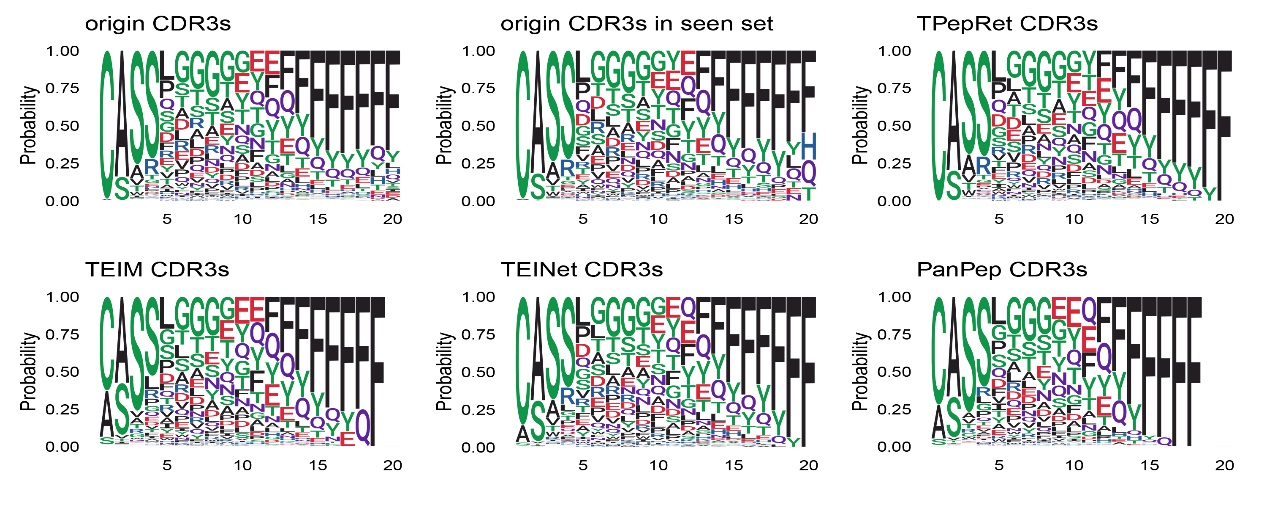


RAKFKQLL


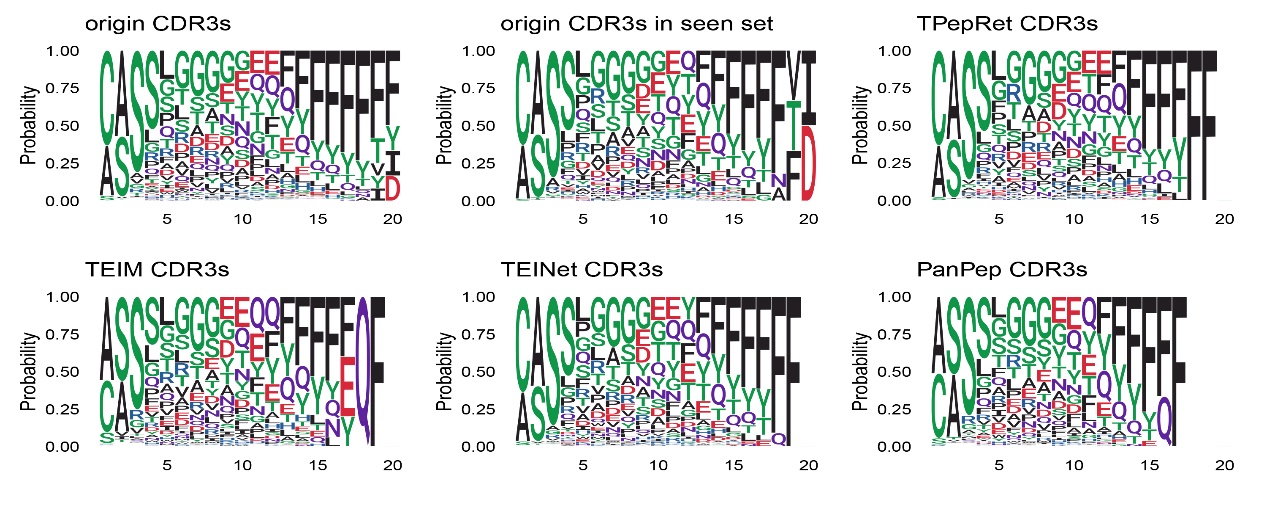


TPRVTGGGAM


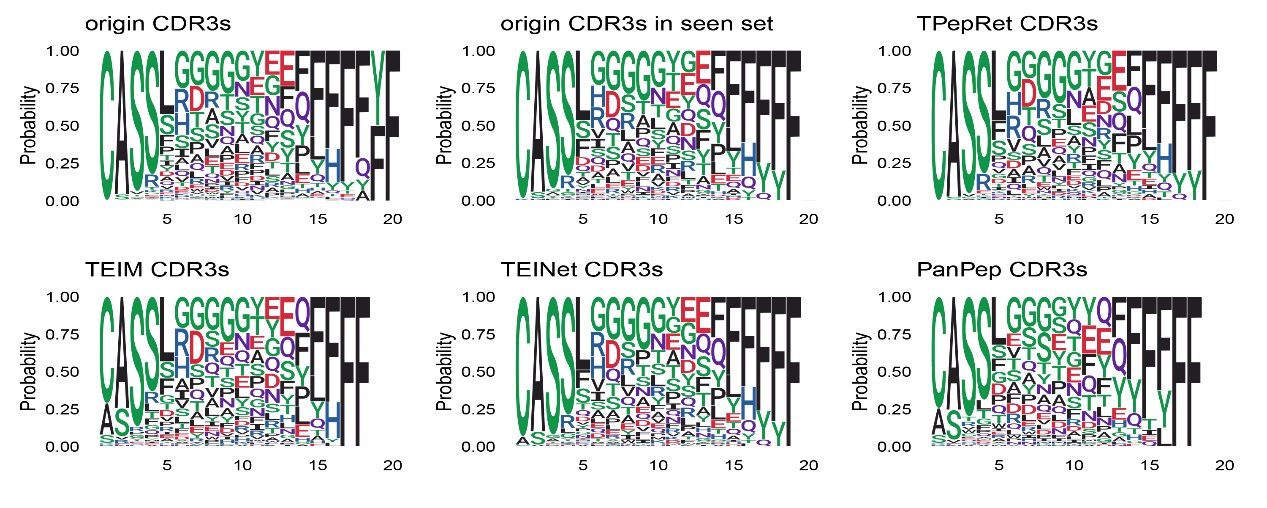


VTEHDTLLY


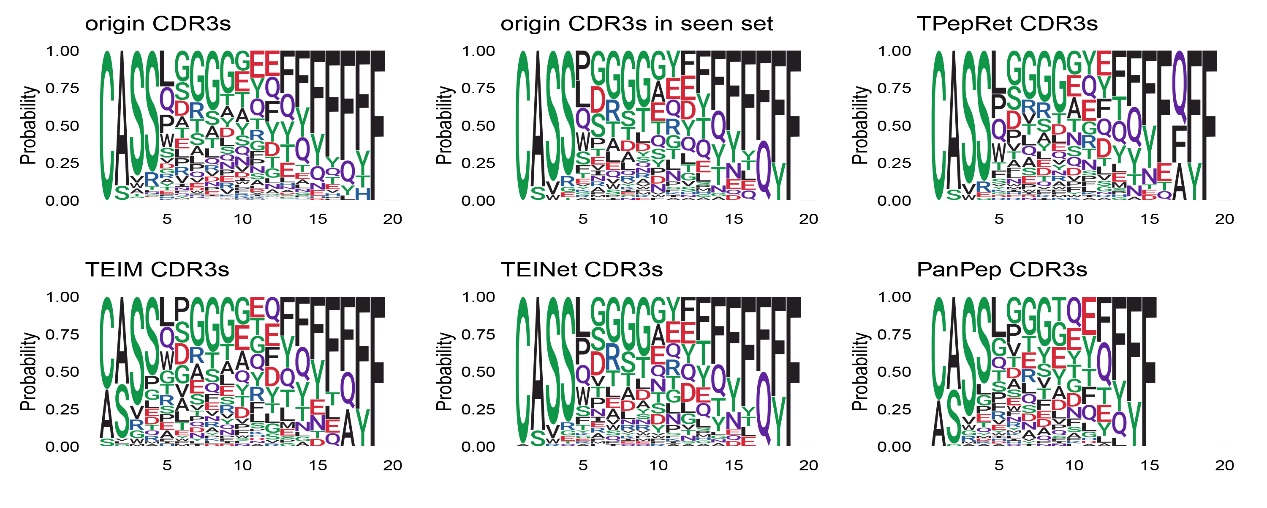


YVLDHLIVV


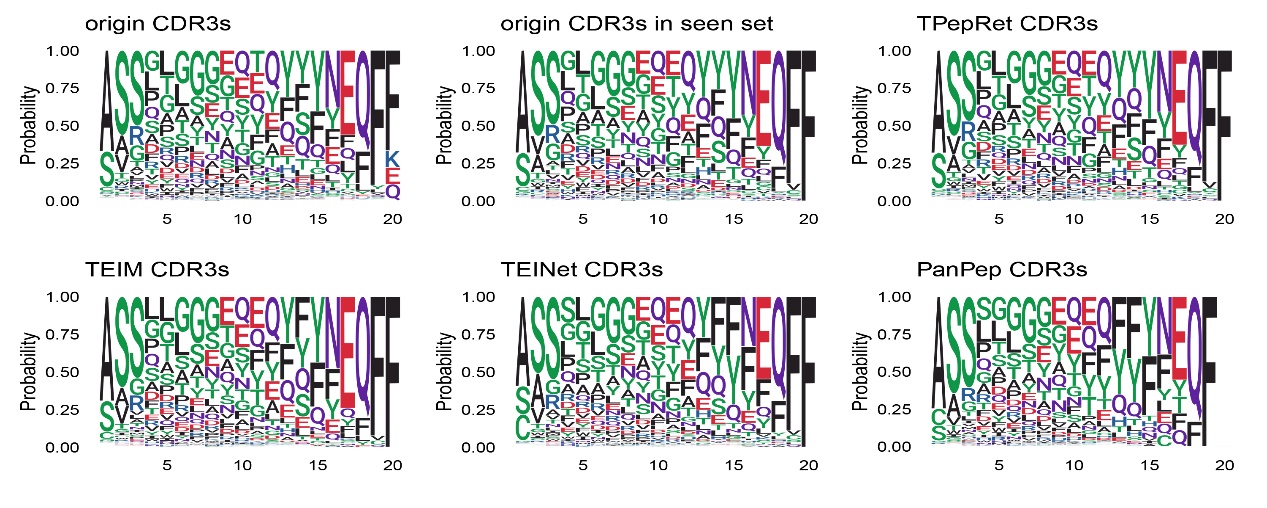


### Supplement Figure S6 The t-SNE distributions of positive and negative samples in the train set (left), seen set(middle), and predicted result set (right) of another six peptides.


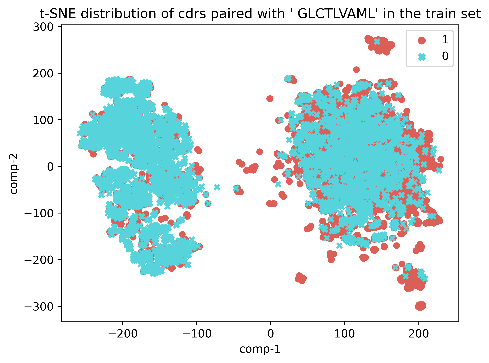

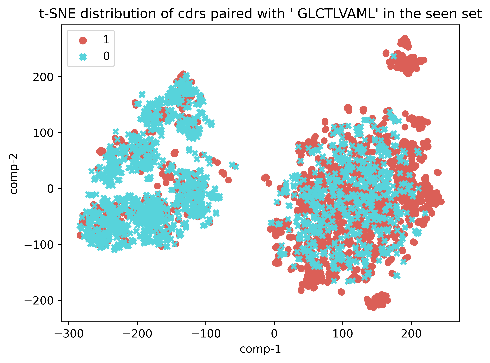

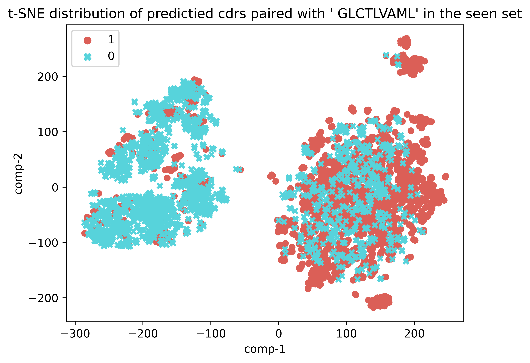


AUC:0.8913


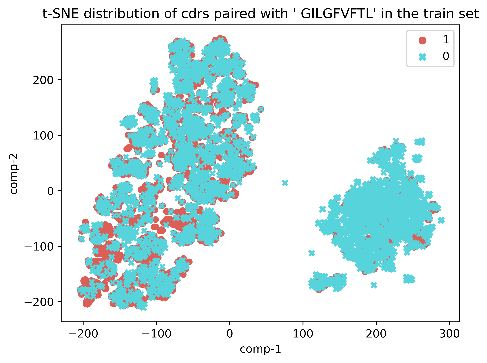

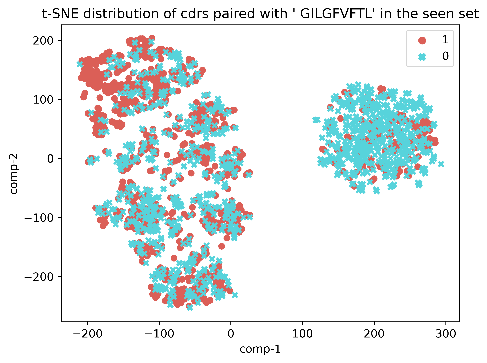

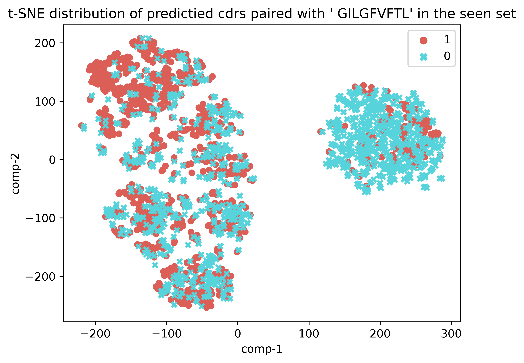


AUC:0.7836


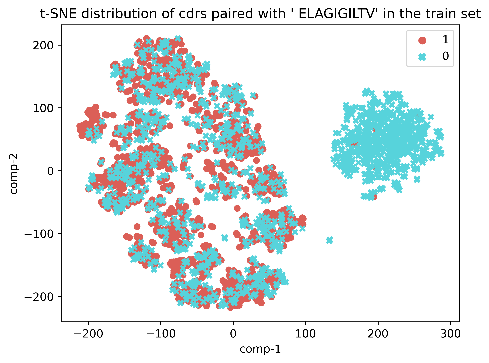

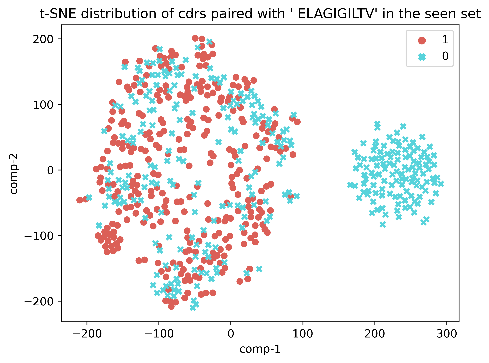

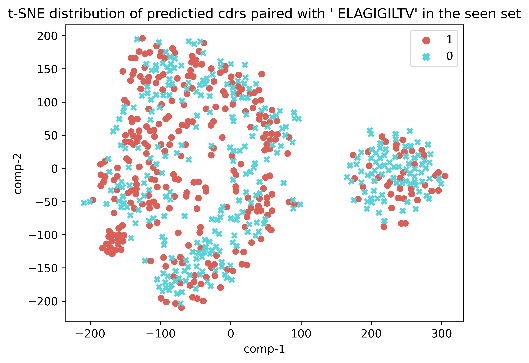


AUC:0.7834


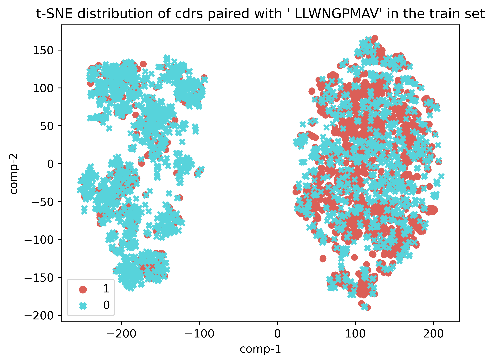

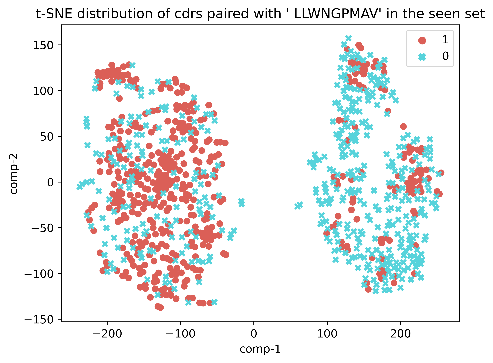

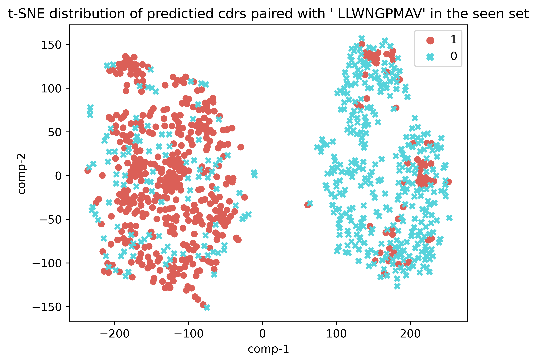


AUC:0.8735


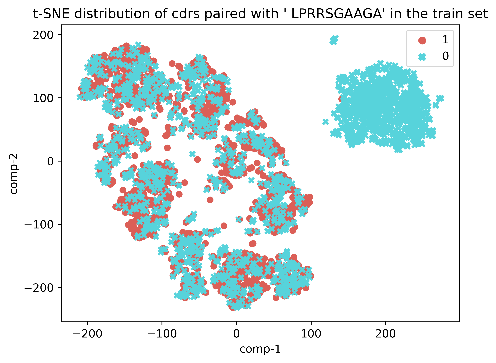

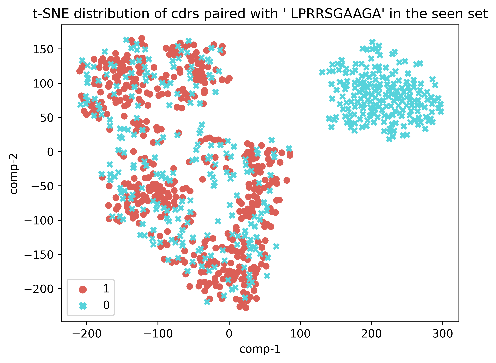

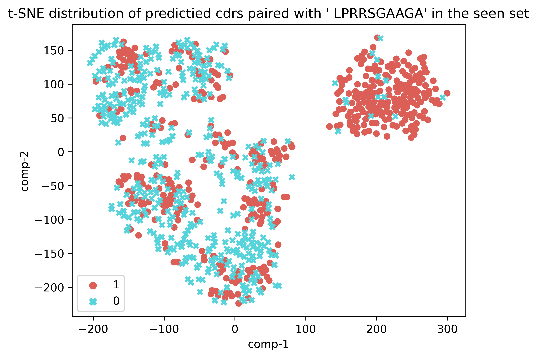


AUC:0.8074


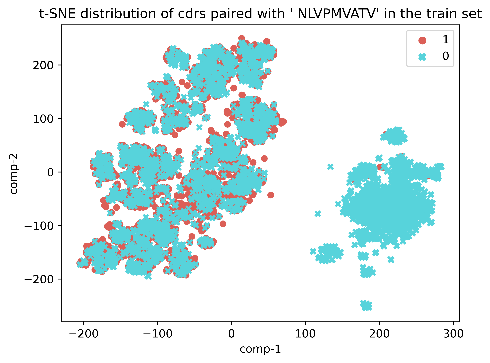

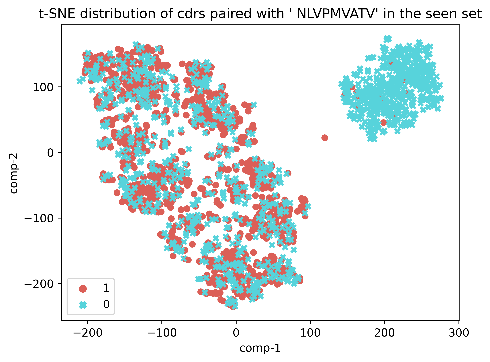

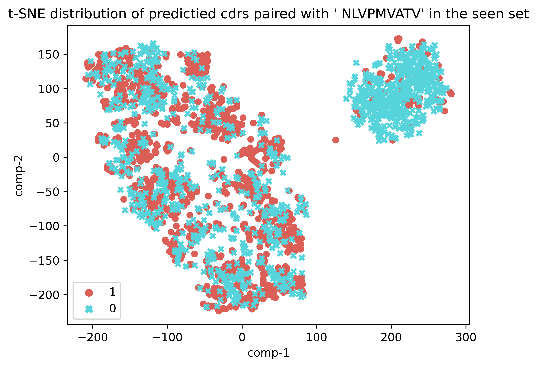


AUC:0.7606

### Supplement Figure S7 Statistical distribution of the changes in predicted results after scanning for ‘alanine’ on the CDR3 sequences.


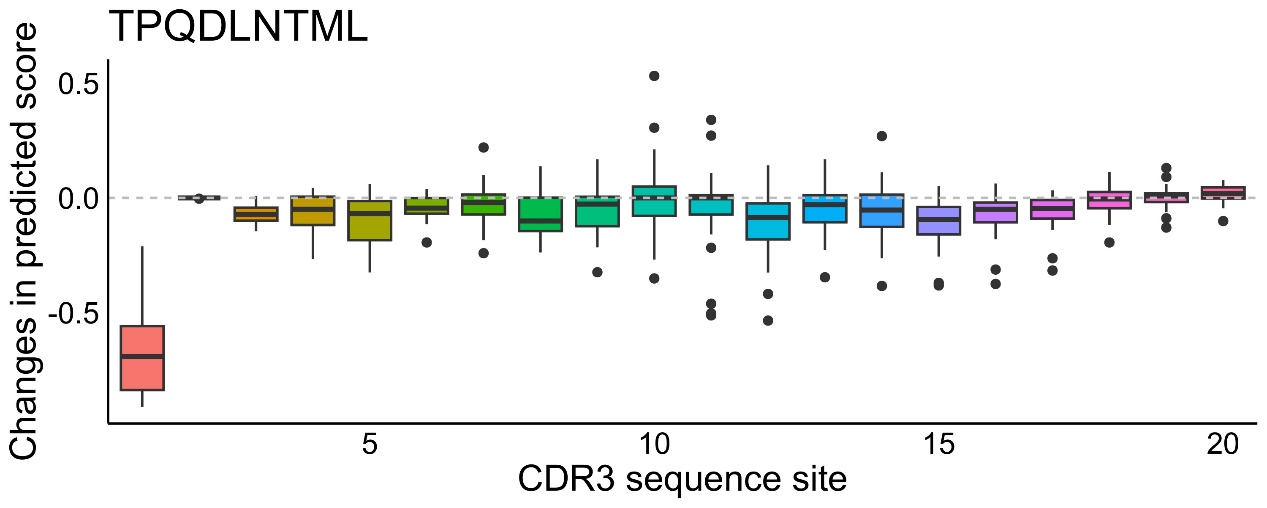

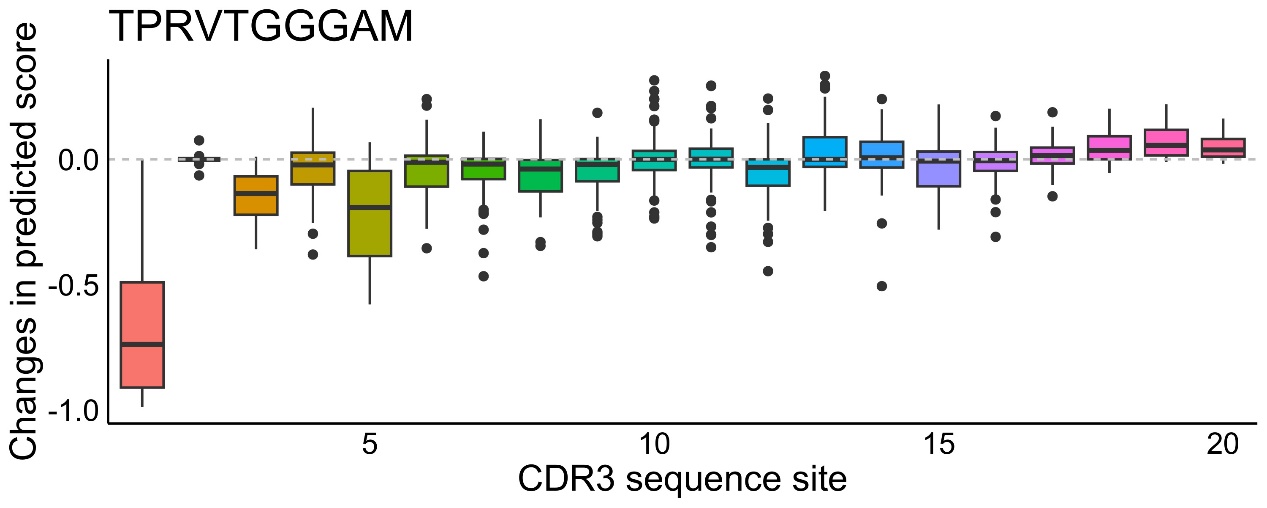

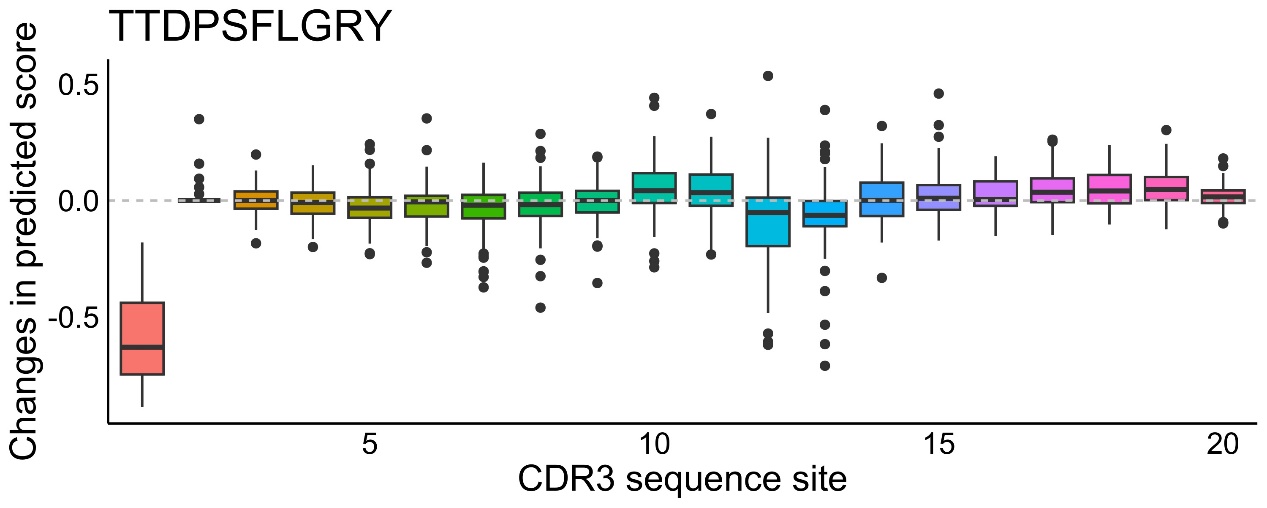

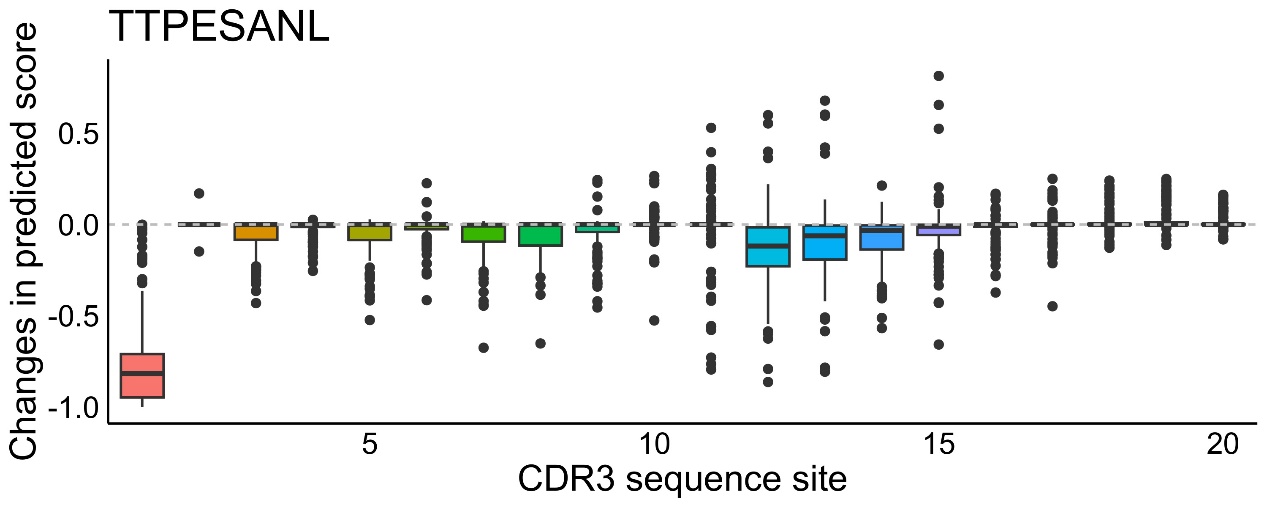

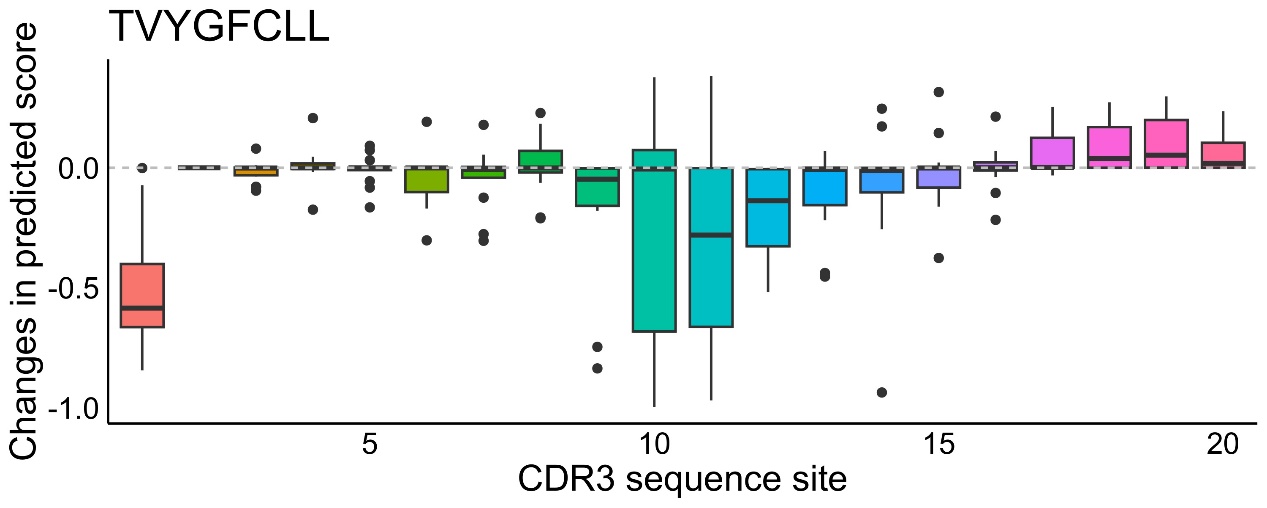

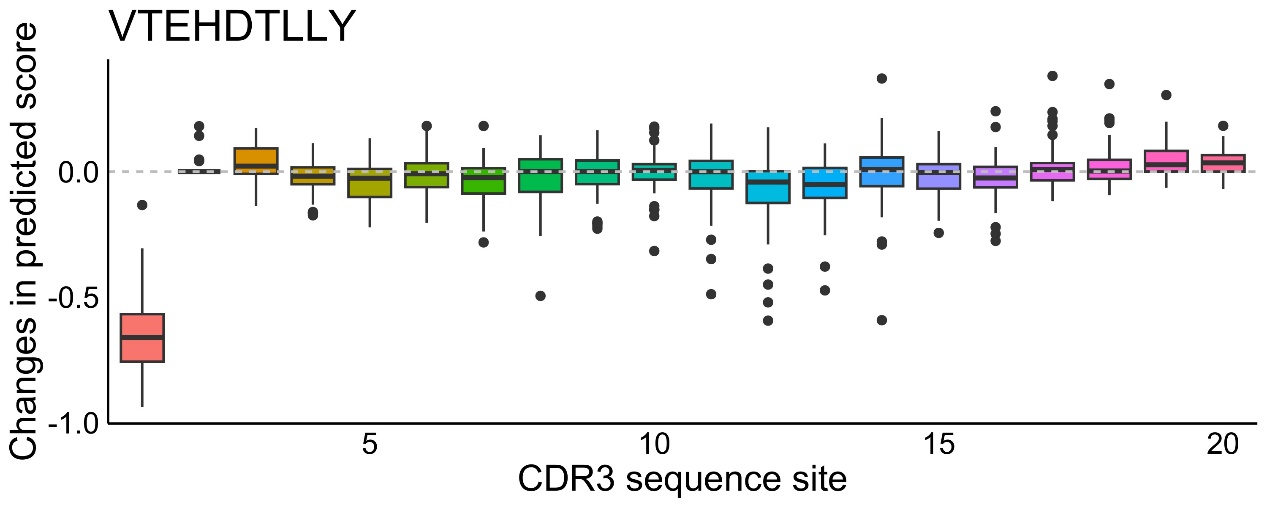

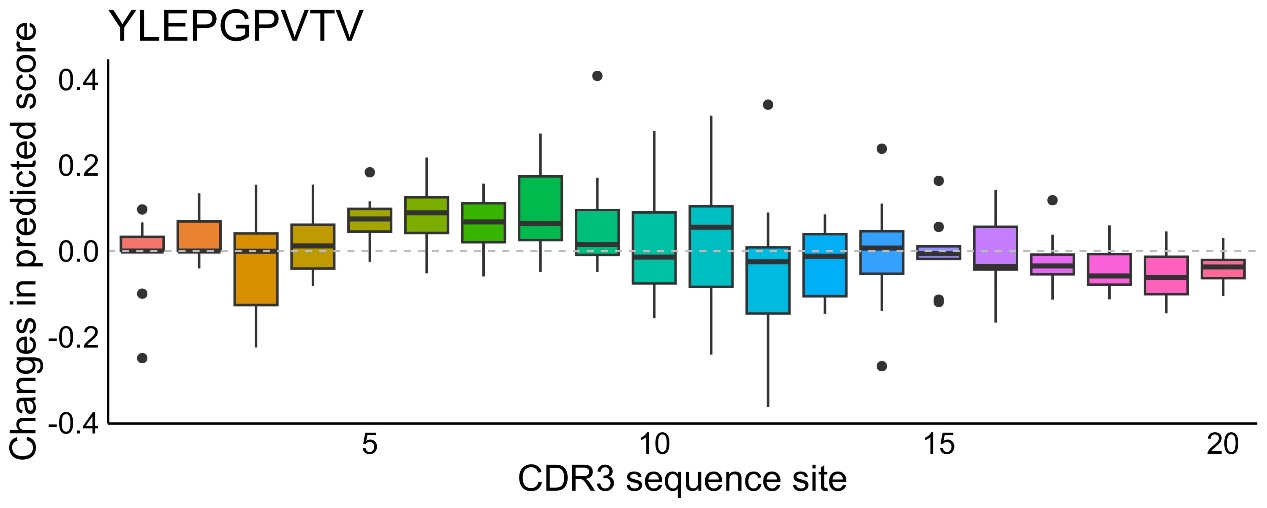

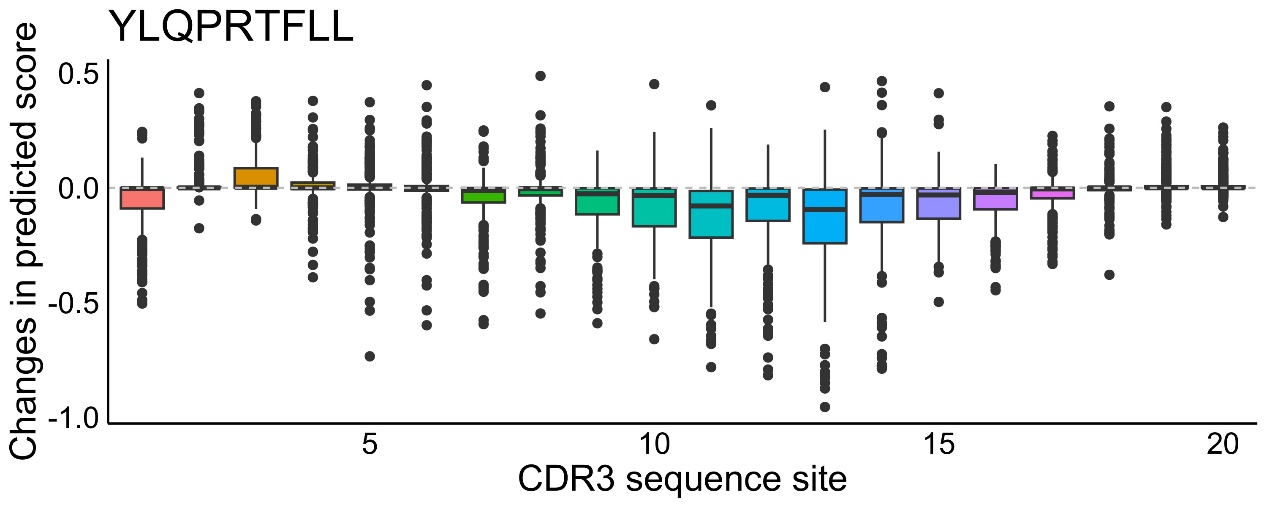

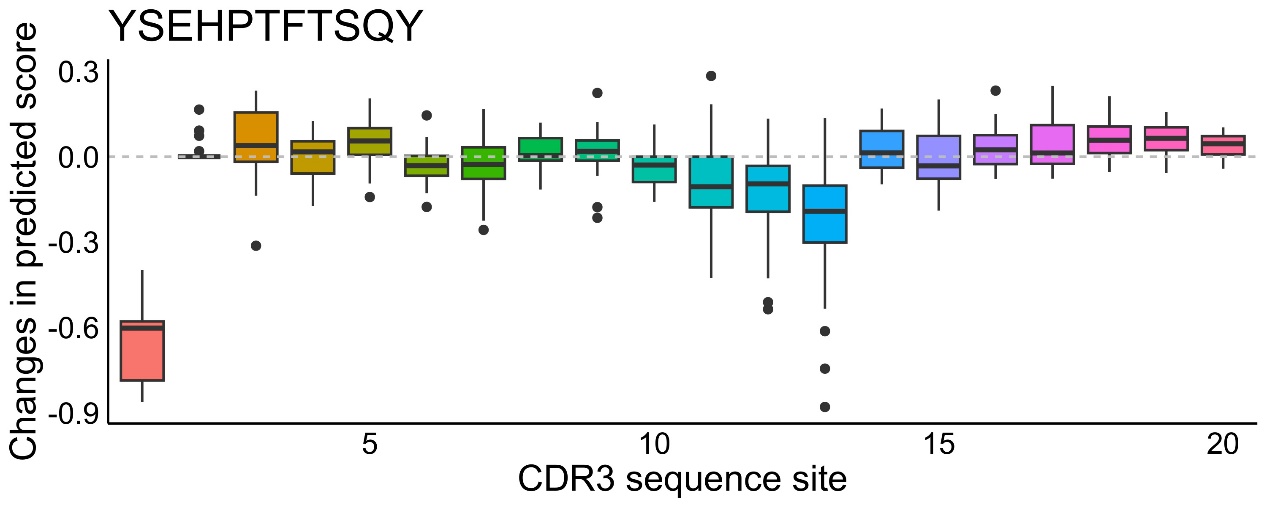

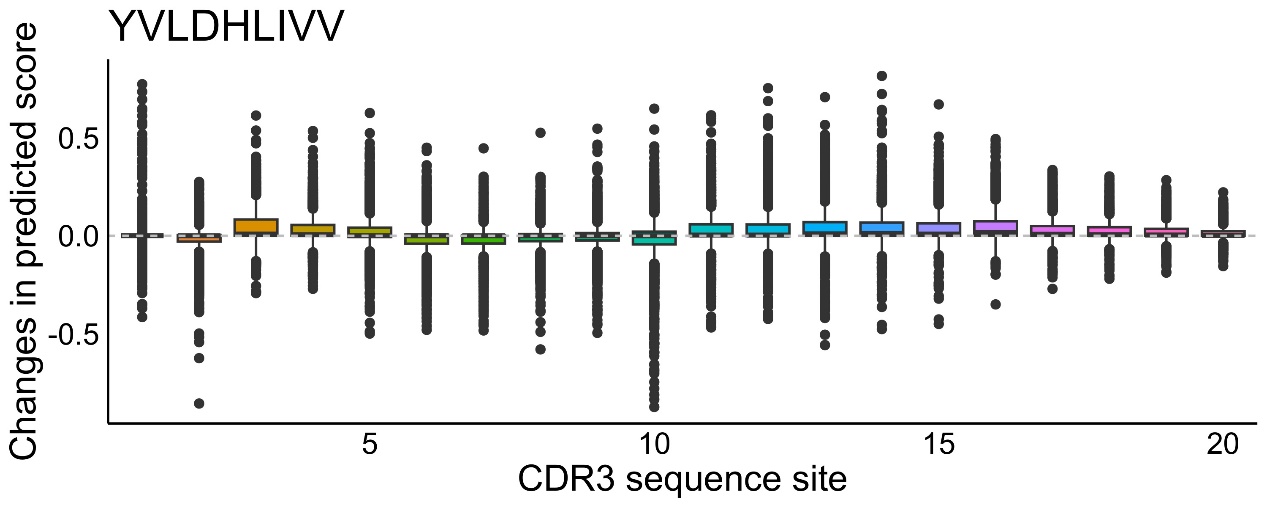

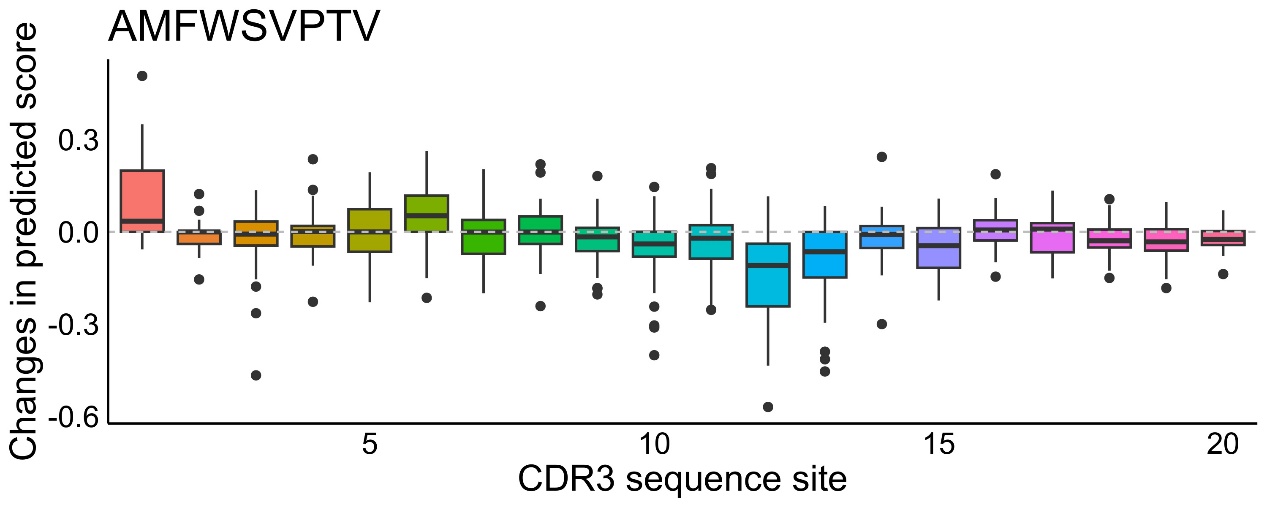

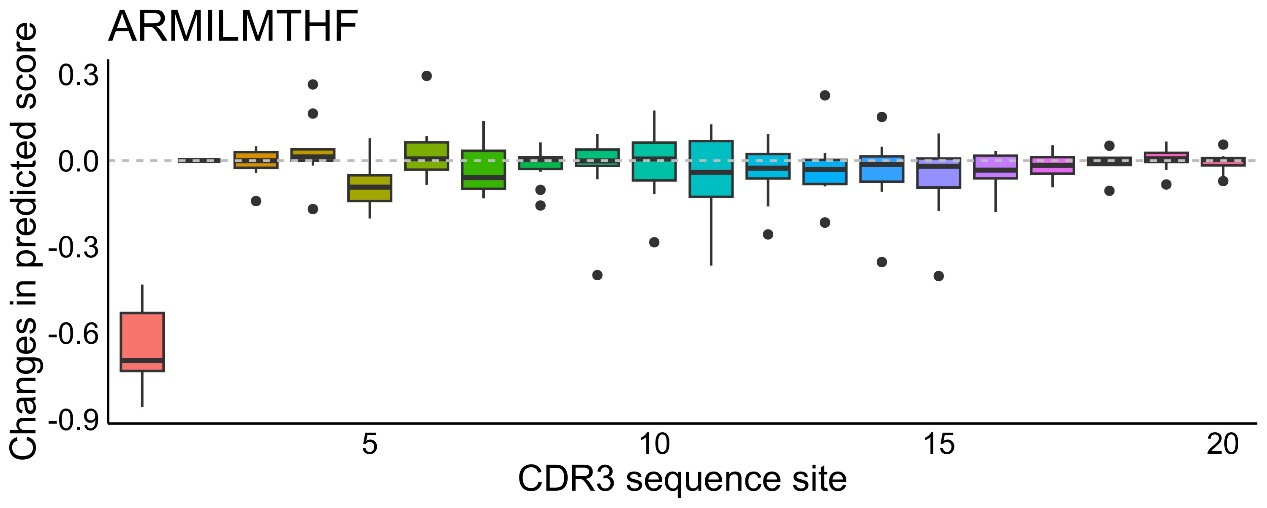

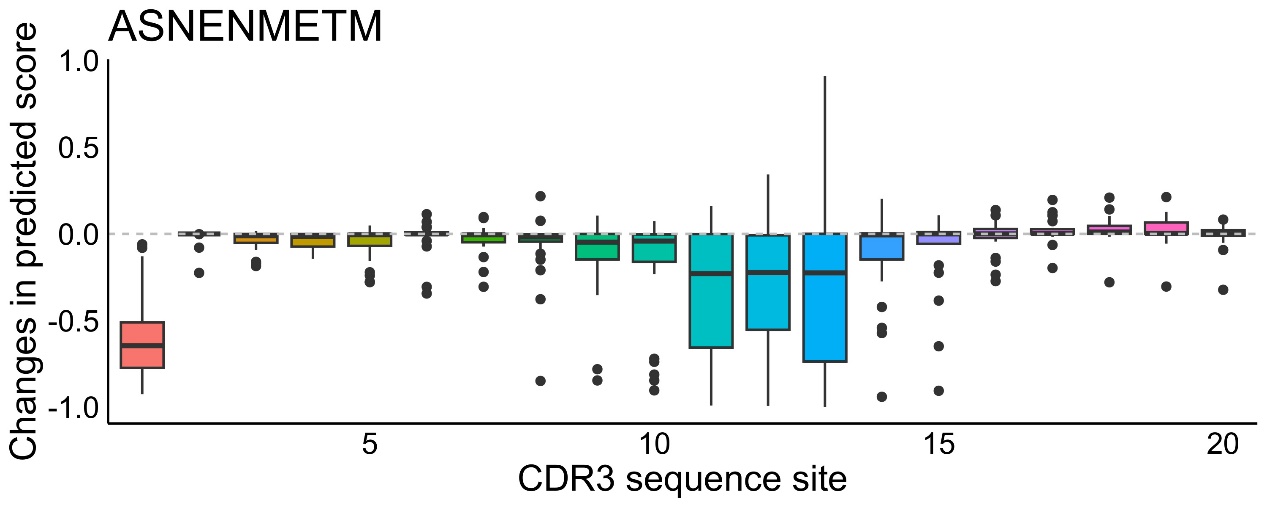

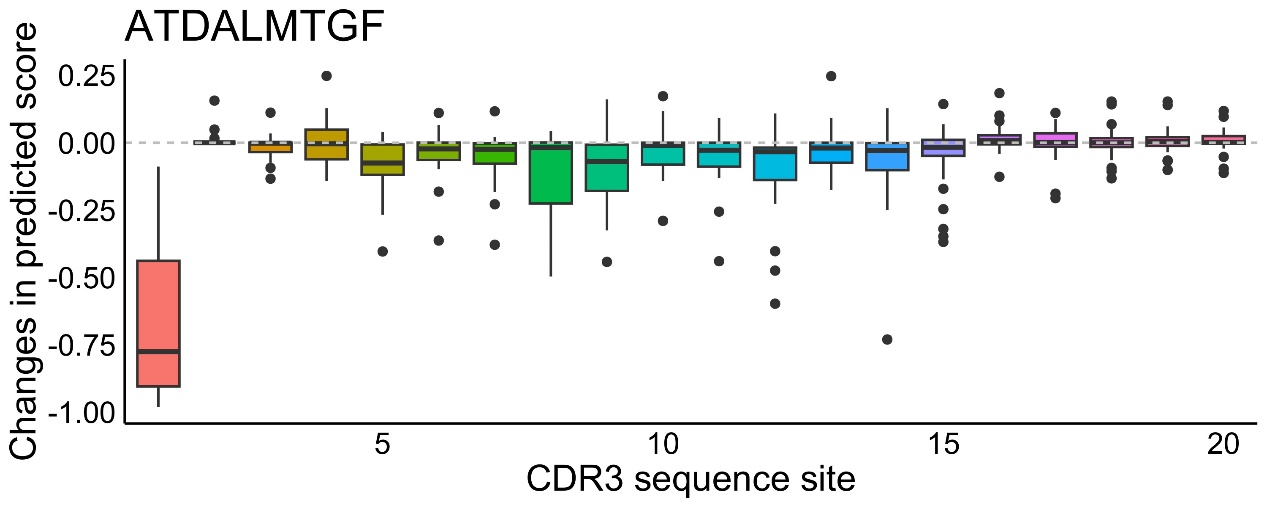

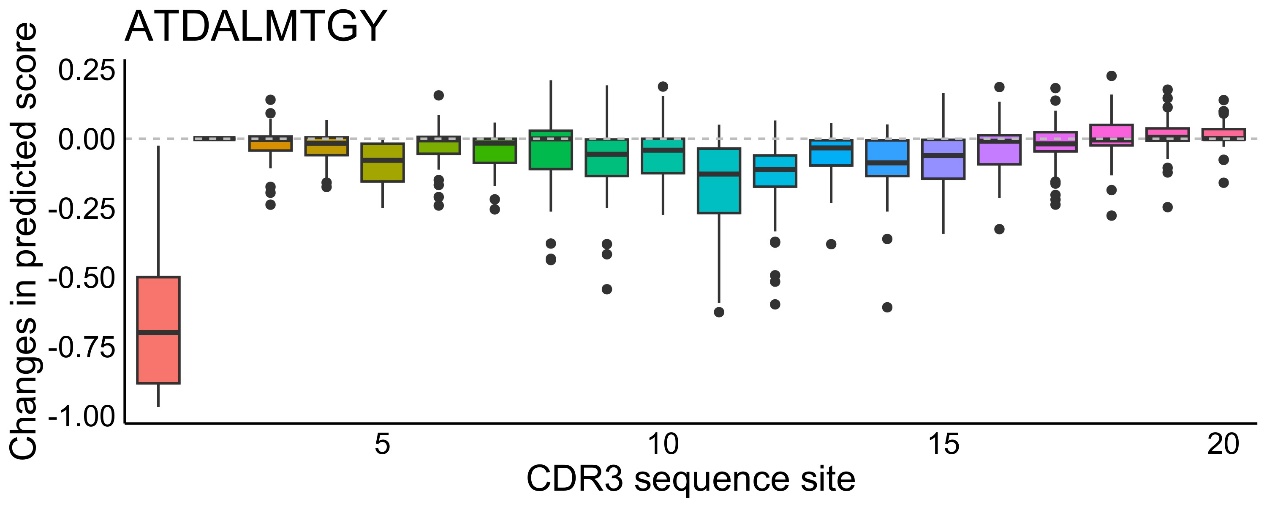

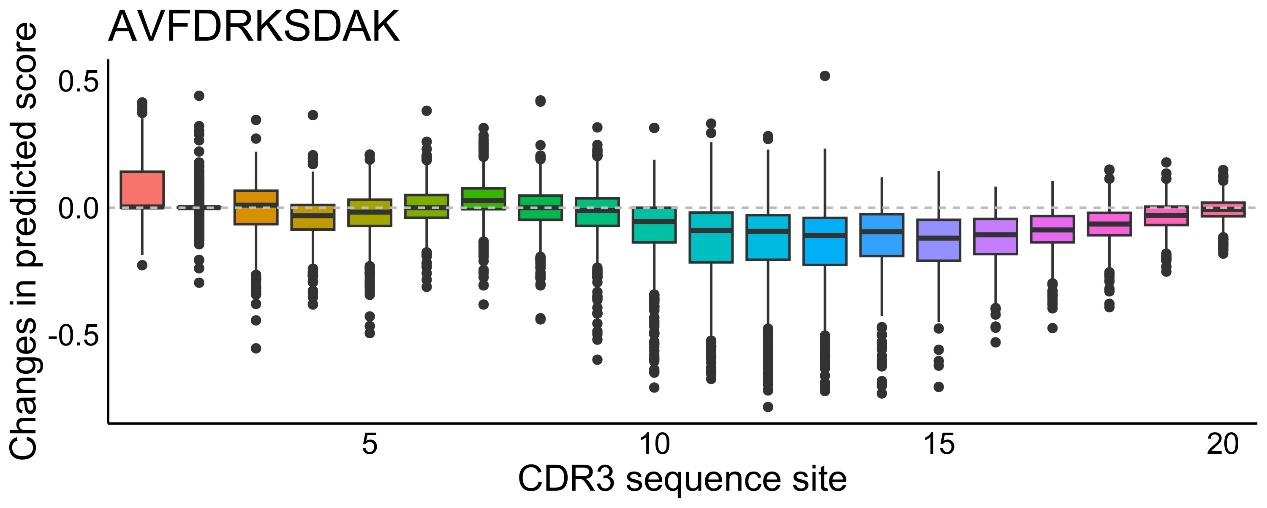

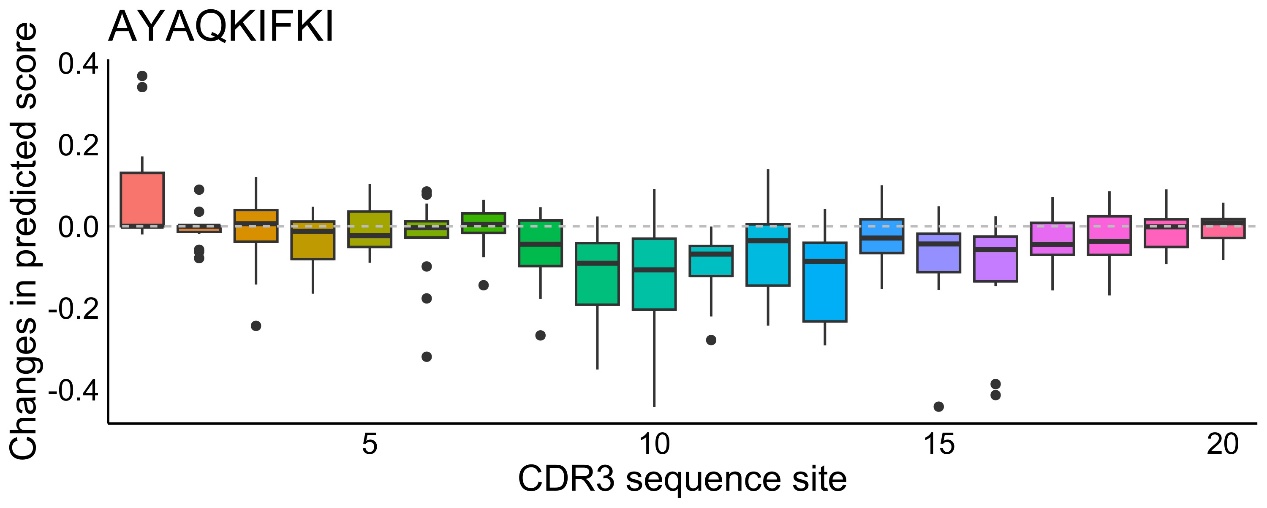

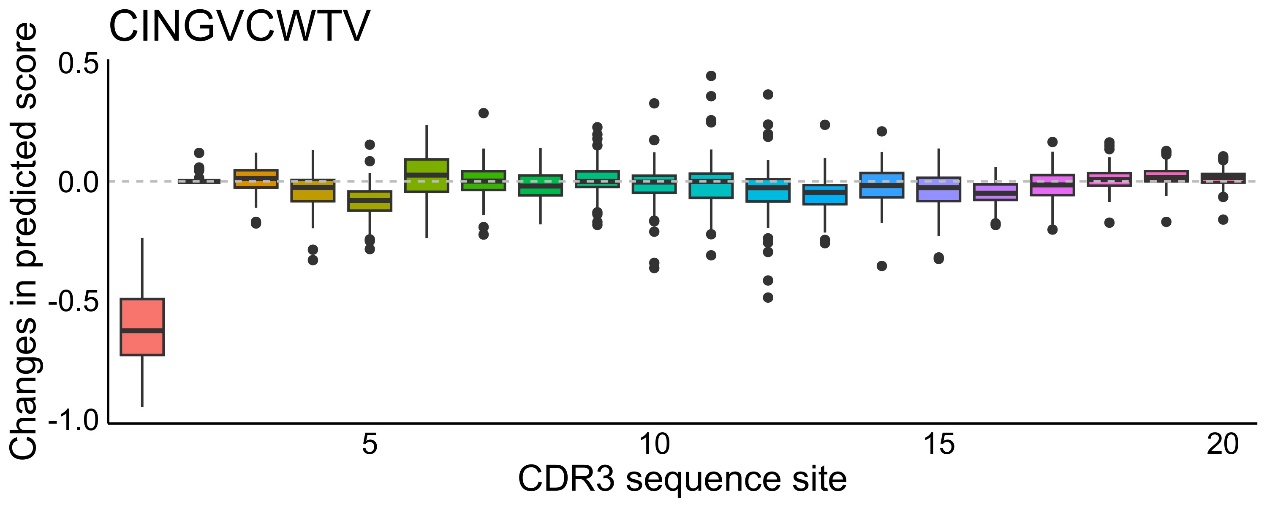

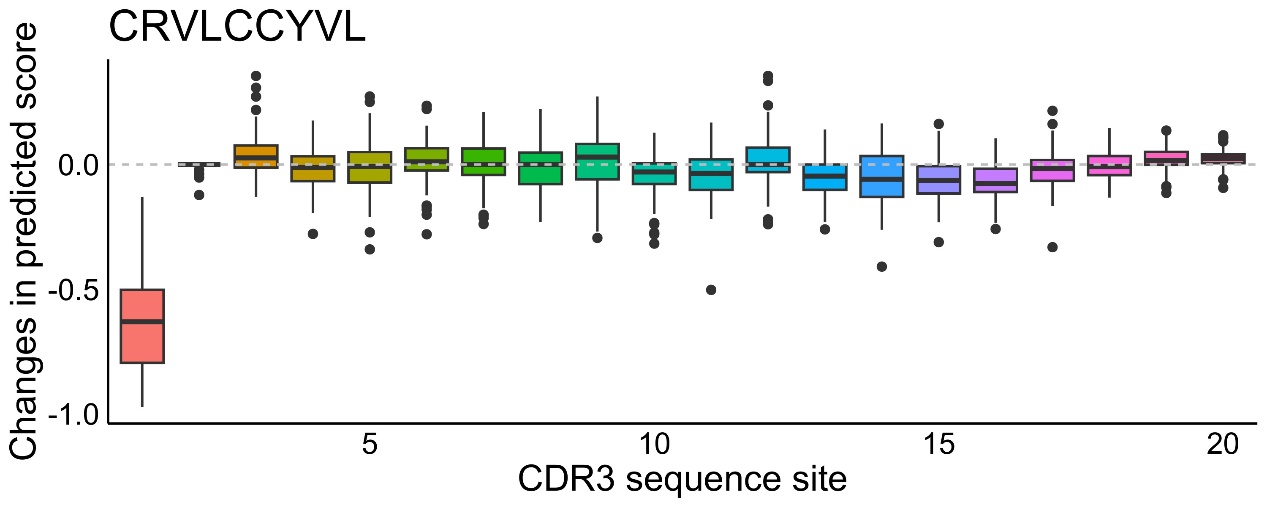

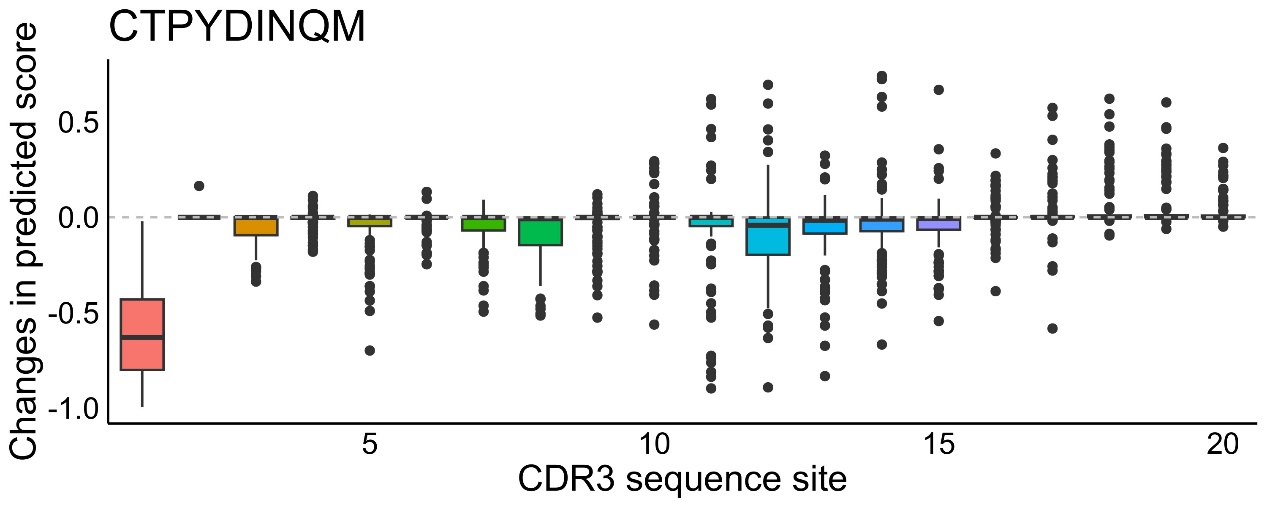

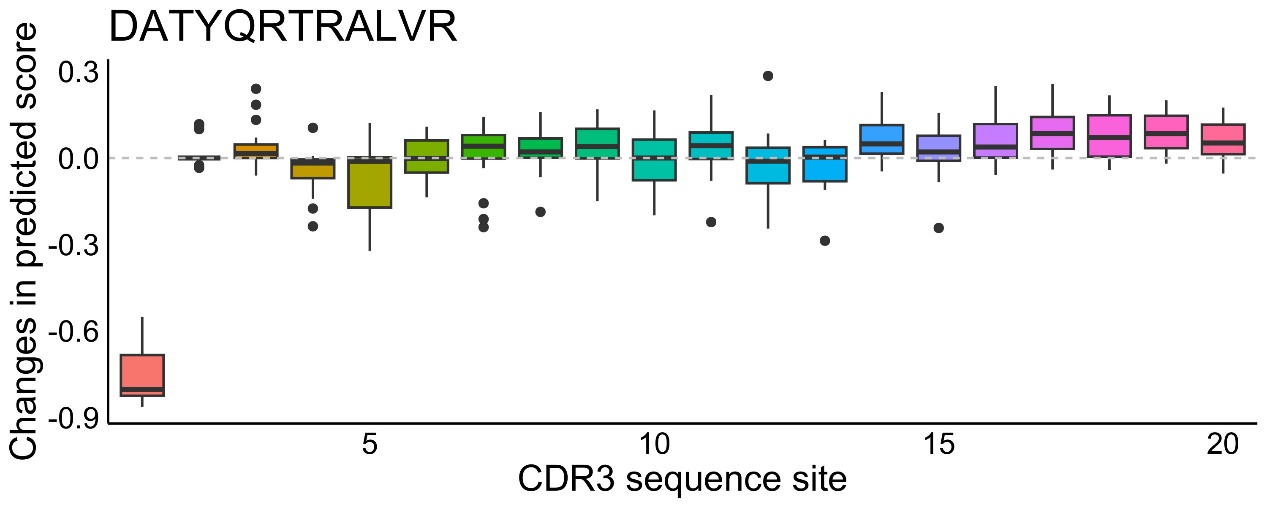

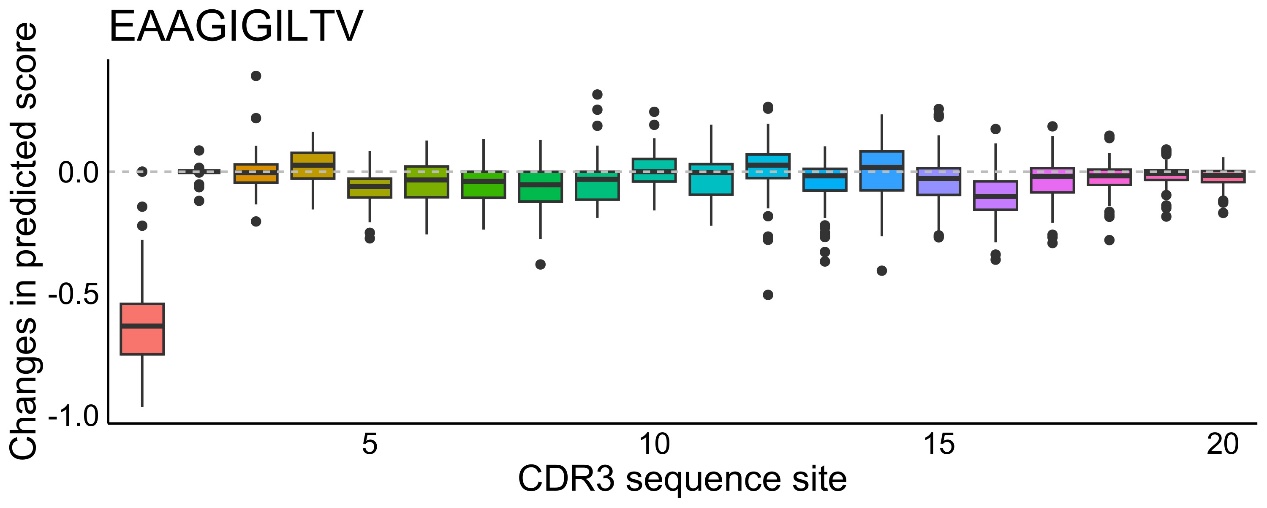

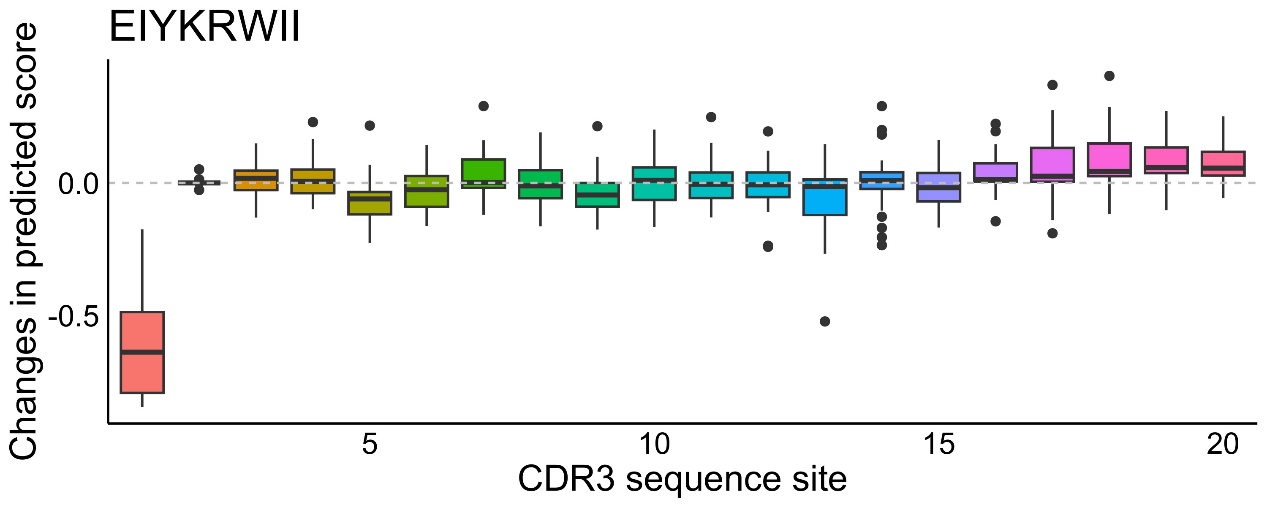

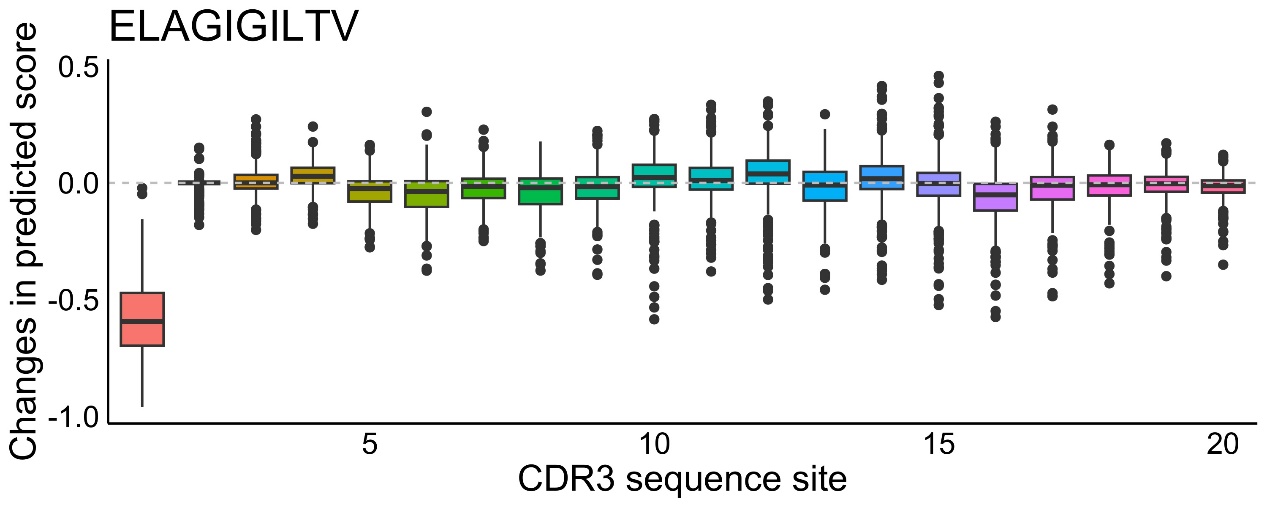

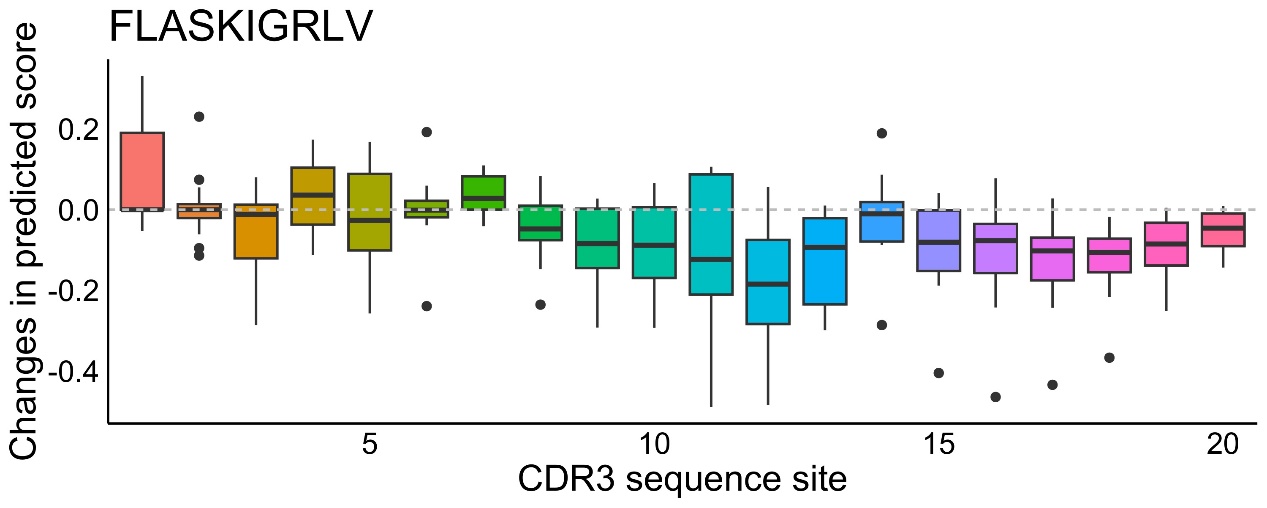

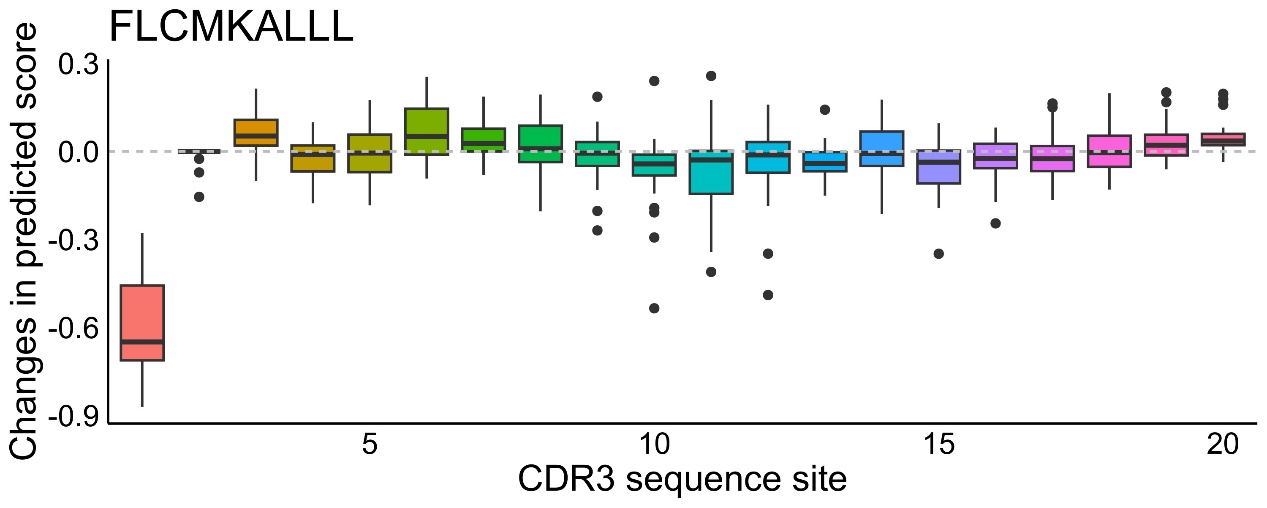

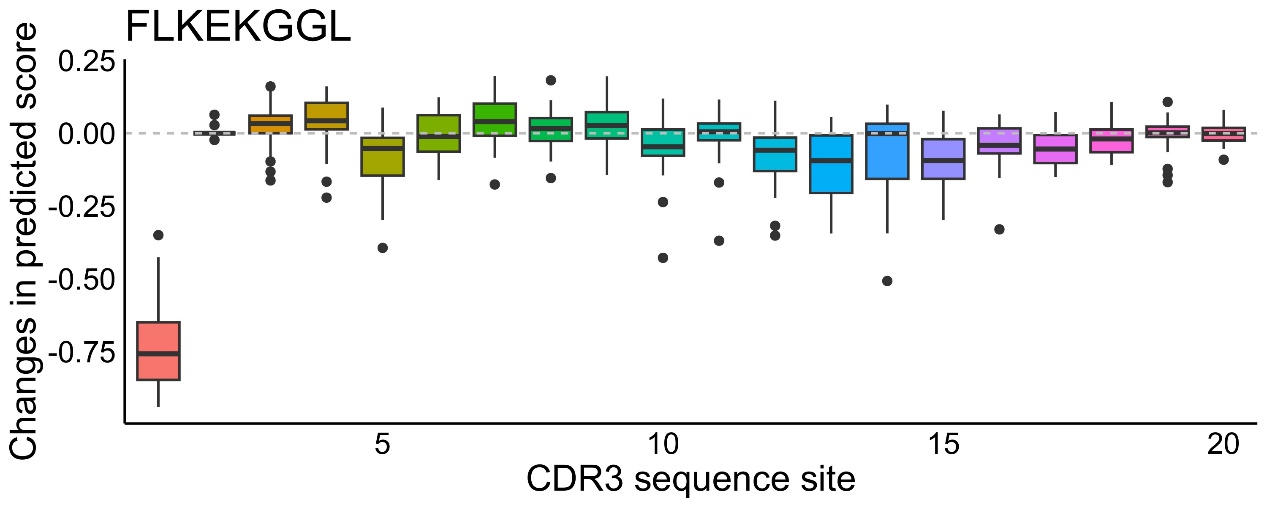

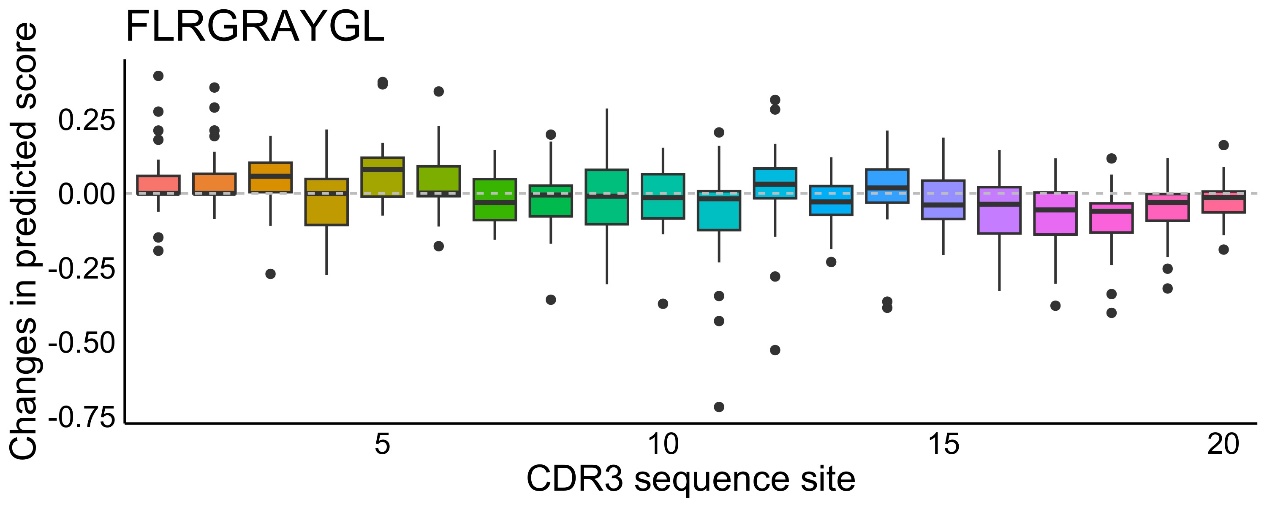

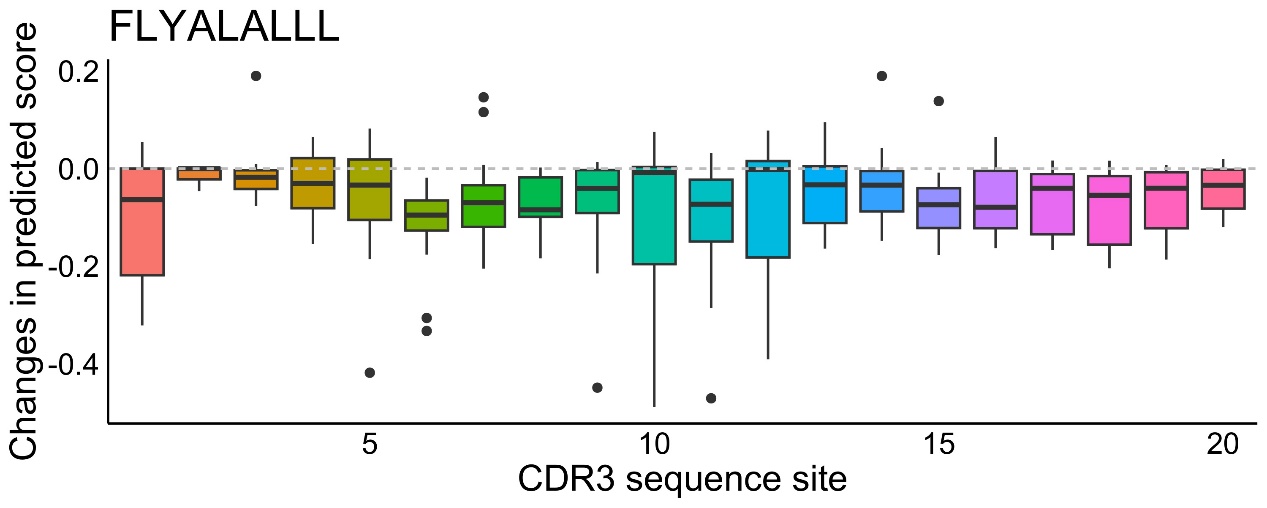

Supplement: btaf022_Supplementary_Data [file btaf022_supplementary_data.zip › ac95e_Supplement Material_TPepRet.docx]
